# Identification of an Arylnaphthalene Lignan Derivative as an Inhibitor against Dengue Virus Serotypes 1 to 4 (DENV-1 to -4) Using a Newly Developed DENV-3 Infectious Clone and Replicon

**DOI:** 10.1128/spectrum.00423-23

**Published:** 2023-06-28

**Authors:** Mingyue Hu, Wan-Fei Li, Tiantian Wu, Yang Yang, Guoquan Chen, Tongling Chen, Yongchen Liu, Yaqing Mei, De Wu, Youchuan Wei, Tingrong Luo, Hong-Jie Zhang, Yi-Ping Li

**Affiliations:** a Institute of Human Virology, Zhongshan School of Medicine, Sun Yat-sen University, Guangzhou, China; b Department of Pathogen Biology and Biosecurity, Zhongshan School of Medicine, Sun Yat-sen University, Guangzhou, China; c Key Laboratory of Tropical Disease Control of Ministry of Education, Zhongshan School of Medicine, Sun Yat-sen University, Guangzhou, China; d College of Animal Science and Veterinary Medicine, Guangxi University, Nanning, China; e School of Chinese Medicine, Hong Kong Baptist University, Kowloon, Hong Kong, China; f Department of Infectious Diseases, The Third Affiliated Hospital of Sun Yat-Sen University, Guangzhou, China; g Institute of Pathogenic Microbiology, Center for Disease Control and Prevention of Guangdong, Guangzhou, China; Shandong First Medical University

**Keywords:** DENV, adaptive mutation, antiviral compound, arylnaphthalene lignans, infectious clone, replicon

## Abstract

Dengue virus (DENV) is the most widespread arbovirus, causing symptoms ranging from dengue fever to severe dengue, including hemorrhagic fever and shock syndrome. Four serotypes of DENV (DENV-1 to -4) can infect humans; however, no anti-DENV drug is available. To facilitate the study of antivirals and viral pathogenesis, here we developed an infectious clone and a subgenomic replicon of DENV-3 strains for anti-DENV drug discovery by screening a synthetic compound library. The viral cDNA was amplified from a serum sample from a DENV-3-infected individual during the 2019 epidemic; however, fragments containing the prM-E-partial NS1 region could not be cloned until a DENV-3 consensus sequence with 19 synonymous substitutions was introduced to reduce putative Escherichia coli promoter activity. Transfection of the resulting cDNA clone, plasmid DV3syn, released an infectious virus titer of 2.2 × 10^2^ focus-forming units (FFU)/mL. Through serial passages, four adaptive mutations (4M) were identified, and addition of 4M generated recombinant DV3syn_4M, which produced viral titers ranging from 1.5 × 10^4^ to 6.7 × 10^4^ FFU/mL and remained genetically stable in transformant bacteria. Additionally, we constructed a DENV-3 subgenomic replicon and screened an arylnaphthalene lignan library, from which C169-P1 was identified as exhibiting inhibitory effects on viral replicon. A time-of-drug addition assay revealed that C169-P1 also impeded the internalization process of cell entry. Furthermore, we demonstrated that C169-P1 inhibited the infectivity of DV3syn_4M, as well as DENV-1, DENV-2, and DENV-4, in a dose-dependent manner. This study provides an infectious clone and a replicon for the study of DENV-3 and a candidate compound for future development against DENV-1 to -4 infections.

**IMPORTANCE** Dengue virus (DENV) is the most prevalent mosquito-transmitted virus, and there is no an anti-dengue drug. Reverse genetic systems representative of different serotype viruses are invaluable tools for the study of viral pathogenesis and antiviral drugs. Here, we developed an efficient infectious clone of a clinical DENV-3 genotype III isolate. We successfully overcame the instability of flavivirus genome-length cDNA in transformant bacteria, an unsolved issue for construction of cDNA clones of flaviviruses, and adapted this clone to efficiently produce infectious viruses following plasmid transfection of cell culture. Moreover, we constructed a DENV-3 subgenomic replicon and screened a compound library. An arylnaphthalene lignan, C169-P1, was identified as an inhibitor of virus replication and cell entry. Finally, we demonstrated that C169-P1 exhibited a broad-spectrum antiviral effect against the infections with DENV-1 to -4. The reverse genetic systems and the compound candidate described here facilitate the study of DENV and related RNA viruses.

## INTRODUCTION

Dengue virus (DENV) is the most widespread arbovirus mainly transmitted by *Aedes* mosquitoes, and its infection causes a spectrum of diseases varying from mild dengue fever to severe dengue, such as dengue hemorrhagic fever (DHF) and dengue shock syndrome (DSS) (1). More than 120 countries have reported DENV infections, and an estimated 3.9 billion people are living with the risk of DENV infection (2). To date, there is no antiviral drug approved for the treatment of DENV infection. It is widely believed that the threat of DENV will continue, and the World Health Organization (WHO) announced dengue as one of the top 10 threats to global health in 2019 (3).

DENV belongs to the genus *Flavivirus* in the family *Flaviviridae*, with a positive single-stranded RNA genome of 10.7 kb consisting of 5′ and 3′ untranslated regions (UTRs) and an open reading frame (ORF). The ORF is translated into a polyprotein precursor, which is further cleaved to generate three structural proteins (capsid [C], precursor membrane [prM], and envelope [E]) and seven nonstructural proteins (NS1, NS2A, NS2B, NS3, NS4A, NS4B, and NS5) (4). The structural proteins compose the morphology of virus particles and play important roles in cell entry and virion morphogenesis. The E protein is on the surface of the dengue virion involved in cell fusion, virus assembly, and antibody neutralization, and the E sequence may evolve faster than other parts of the viral genome (5). The prM protein is cleaved to M by the cellular protease furin during egress through the trans-Golgi network, thus allowing the rearrangement of the M-E complex on the mature particle (6–8). Virion assembly requires C protein binding to progeny viral RNA to form the nucleocapsid, which buds into the endoplasmic reticulum (ER) lumen as an immature virion consisting of icosahedral prM/E heterotrimers (9). The nonstructural proteins are mostly responsible for the replication and translation of viral genome and interaction with host factors important for the completion of viral life cycle (10).

Although five serotypes of DENV have been described, only four serotypes (DENV-1, -2, -3, and -4) are known to be transmitted in humans. Each of these four DENV serotypes can be subdivided into distinct genotypes (11–13). DENV-3 has five genotypes (I to V) (14, 15), and the genotypes generally differ from each other by ~6 to 8% at the nucleotide level and 3% at the amino acid level (16). Sequence divergence between serotypes is ~30 to 35% at the amino acid level (17), accounting for (at least in part) the virological and immunological outcomes and disease manifestations, which may differ among patients. Therefore, infection models representative of various serotypes and genotypes are required.

Reverse genetic systems represent an invaluable tool for the study of molecular virology, replication and translation, virulence determinants, vaccines, antiviral drugs, etc., with great convenience for genetic manipulations (18, 19). In recent decades, different approaches have been applied to develop genome-length infectious clones for DENV (20–24), including DENV-3 (19, 25–27), and other flaviviruses (28, 29). However, the instability of genomic cDNA clones of flaviviruses in transformed bacteria remains an unresolved issue (23, 30, 31), resulting in unclear underlying mechanisms, which greatly hampers the development of infectious culture systems for clinical isolates and the utility of an infectious clone. Thus, a genome-length infectious clone with genetic stability in transformant bacteria would be invaluable to the field.

Plant extracts represent promising resources for drug development, and an estimated 15.6% of drugs are derived from plants (32). DENV infectious clones have contributed greatly to antiviral studies, such as screening of compound libraries and determining antiviral mechanisms of candidate compounds. Studies have discovered a number of natural compounds with activity against DENV (33, 34), which could constitute candidates for future anti-dengue drug discovery. Previously, we found that patentiflorin A and justiprocumin B, arylnaphthalene lignans extracted from Justicia gendarussa, exhibited activity against HIV (35, 36). In this study, we constructed a genome-length infectious cDNA clone of a DENV-3 genotype III clinical isolate and a DENV-3 subgenomic replicon. We identified C169-P1 as an inhibitor against DENV-1 to -4 through screening a library of arylnaphthalene lignan derivatives.

## RESULTS

### Genome sequence of DENV-3 genotype III isolate D191267.

**FIG 1:**
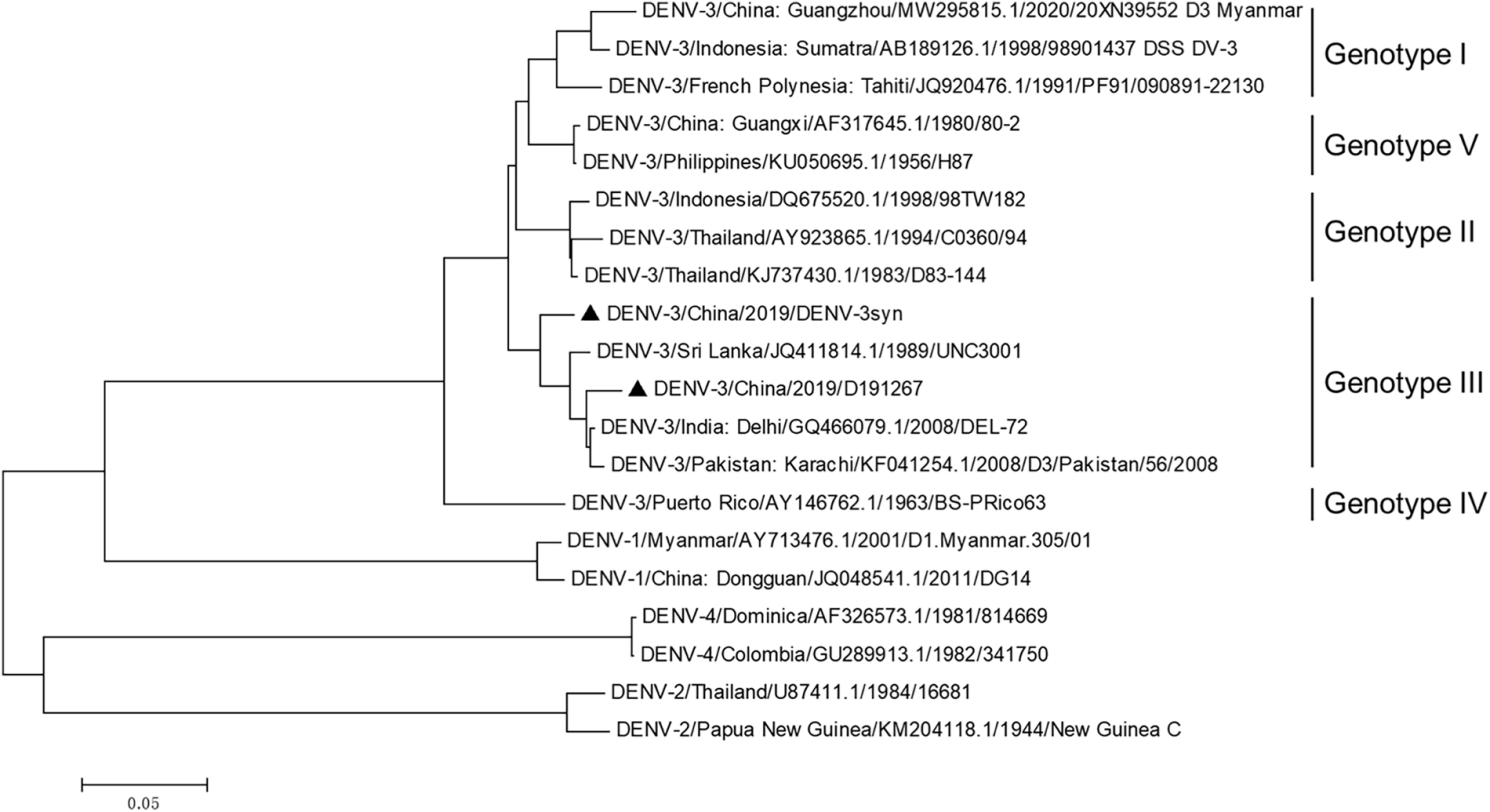
Phylogenetic analysis of DENV-3 isolate D191267 and other serotypes of DENV isolates. The phylogenetic tree was generated using the neighboring-joining method in MEGA6 software. The sequences of DENV-3 genotypes I to V with complete ORF sequences available in GenBank were selected for this phylogenetic analysis. In addition, two sequences each were selected from DENV-1, DENV-2, and DENV-4. Solid triangles highlight the original D191267 and recombinant DV3syn (DENV-3/China/2019/DENV-3syn), which was the wild-type sequence of the infectious clone DV3syn_4M.

A serum sample, D191267, was collected from a DENV-3-infected patient in the 2019 epidemic in Qingyuan, Guangdong Province, China. To make origin virus stock, patient serum was inoculated into C6/36 cells, and the culture supernatants were collected at 6 days postinfection (dpi). Total RNA was extracted and amplified by four overlapping DENV-3-specific reverse transcription-PCRs (RT-PCRs) (see Table S1 in the supplemental material). Sequencing analysis of four PCR products yielded the sequence covering nucleotides (nt) 54 to 10623 of the virus genome (GenBank no. OQ948473). Phylogenetic analysis revealed that D191267 belonged to genotype III of DENV-3 (Fig. 1) and had the highest sequence homology to isolate GZ8H/2019149/2019/III (MW720883.1), confirming that D191267 was a genotype III DENV-3 isolate. To further characterize the D191267 isolate, we aligned the D191267 sequence with the consensus ORF sequences of DENV-3 isolates from Africa, Asia, Europe, South America, North America, and Oceania, retrieved from GenBank (Fig. S2). D191267 had high homology to the Africa DENV-3 consensus sequence (Fig. S3 and Table S3). DENV-3 re-emerged in the Canton region of China in 2009 and 2010, after the early epidemic in 1980 (13, 37), and it was introduced from different geographical regions, including Southeast Asia, South America, Africa, and the Middle East (37). A reverse genetic system for a DENV-3 genotype III isolate would be a valuable tool for the study of DENV-3, which is currently prevalent in south China and other regions.

### Synonymous mutations reducing putative ECP enabled assembly of the genome-length DENV-3 clone.

To assemble a genome-length cDNA clone for D191267, we chose to subclone four overlapping PCR products individually into the vector plasmid pMD18-T and transform them into *Turbo* competent cells. However, the fragment spanning nt 54 to 3055 could not be cloned after tremendous efforts; the transformant colonies were hardly recovered, or the recovered sequences contained mutations, deletions, or insertions. We speculated that this region may contain Escherichia coli promoters (ECPs) that potentially transcribed proteins toxic to the transformant bacteria, thus resulting in instability of the viral cDNA sequence (23, 29–31). The ECPs were predicted and the score for the predicted ECPs was calculated by a website (http://www.fruitfly.org/seq_tools/promoter.html) search program (Neural Network promoter prediction) from the Berkeley Drosophila Genome Project. Indeed, 16 ECPs could be predicted in the sequence (nt 437 to 2933) spanning the entire prM and E and the 500 nt of NS1 (referred to here as prM-E-NS1 for simplicity). Ten of these ECPs showed scores of ≥0.9, higher than those predicted in the corresponding region of previously developed infectious clones, DENV-2 (29), Japanese encephalitis virus (JEV) (29), and Zika virus (ZIKV) (31, 38). This fragment could not be cloned after introducing substitutions to reduce the ECP scores; thus, we chose to replace the prM-E-NS1 region of D191267 with a consensus sequence that was deduced from the DENV-3 isolates.

We analyzed a total of 180 DENV-3 sequences for the prM-E-NS1 sequence (nt 437 to 2933), from which a consensus sequence was deduced (Fig. S4). Further, we analyzed the putative ECP in the consensus prM-E-NS1 sequence and identified 19 ECP sites with scores of ≥0.8 (Table S2), indicating the existence of potential ECP activity. We replaced these 19 ECP sites with synonymous mutations to reduce ECP activity (Tables S2 and S4; Fig. S4). With these changes, we finally succeeded in cloning the ECP-reduced prM-E-NS1 fragment covering nt 437 to 2933 into the vector plasmid.

**FIG 2:**
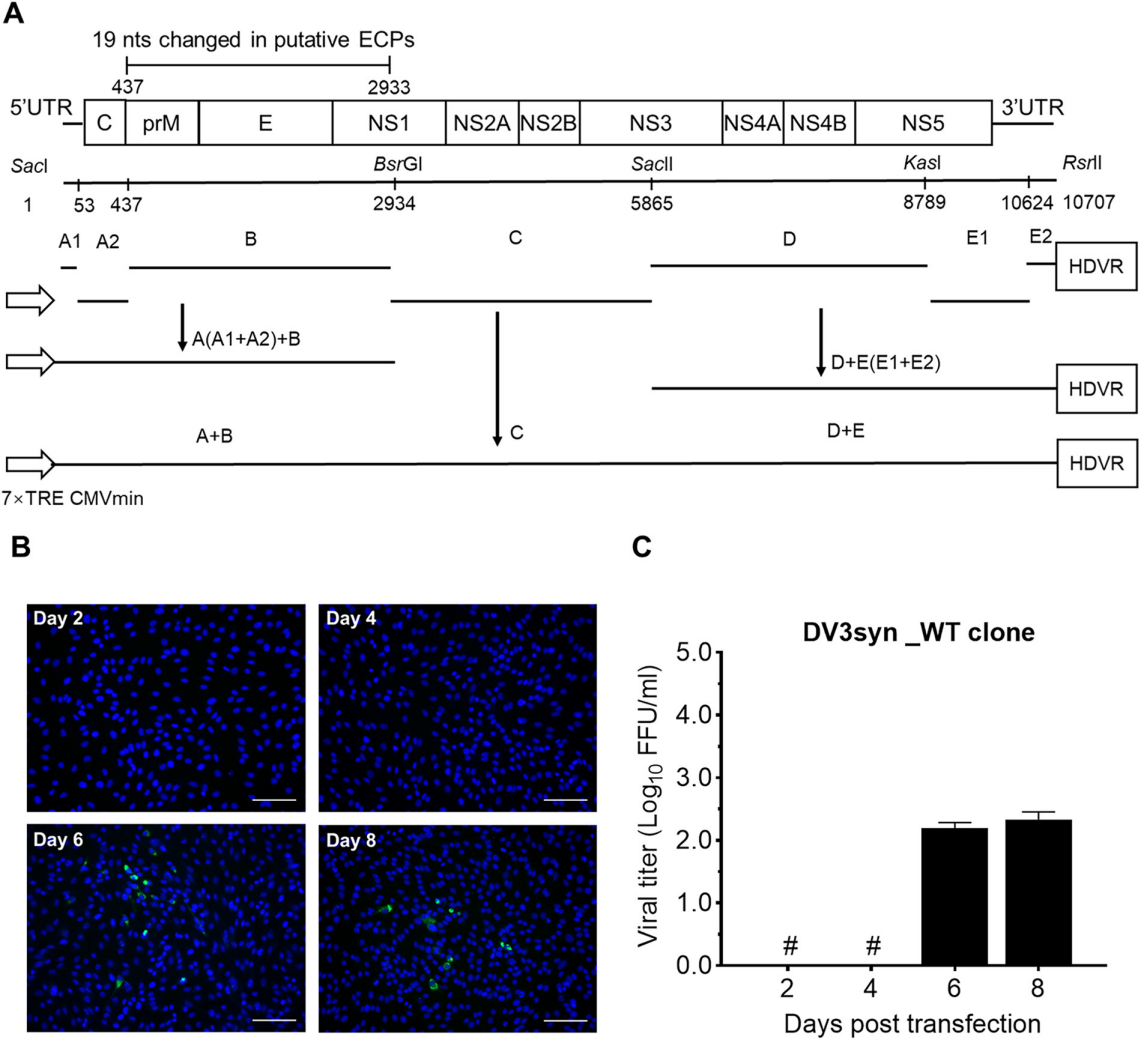
Diagram of genome-length DV3syn clone and production of infectious virus. (A) Schematic diagram of the construction of the genome-length pTight-DV3syn_WT clone. The consensus prM-E-NS1 fragment (nt 437 to 2399) was deduced from alignment of 180 DENV-3 sequences (Fig. S1 and S4), and 19 synonymous mutations were introduced to reduce the ECP activity (Fig. S4 and Table S3). Five overlapping fragments (A to E) were generated, of which fragment A was subdivided into A1 and A2 and fragment E was subdivided into E1 and E2. Restriction sites and nucleotide positions are indicated for fragments A (nt 1 to 455; SacI), B (ECP reduced; nt 418 to 3055; BsrGI), C (nt 2914 to 5952; BsrGI-SacII), D (nt 5812 to 8854; SacII-KasI), and E (nt 8740 to 10707; KasI-RsrII). The modified pTight vector contains 7×TRE and the CMVmin promoter sequence, as well as hepatitis D virus ribosome (HDVR). The order of cloning fragments was as follows: B was fused to A2 to make A2+B by fusion PCR, and then A1 (nt 1 to 53; identical to isolate GZ2D3) was fused with A2+B to make the (A1+A2)+B fragment. The fragment A-B was assembled into pTight vector to make pTight_A-B by in-fusion cloning. Similarly, fragment D was fused to E1 (nt 8740 to 10623) and E2 (nt 10624 to 10707; identical to isolate GZ2D3) using fusion PCR strategies to obtain D+E(E1+E2). The D+E(E1+E2) was cloned into pTight_A-B to make pTight_A-B-D-E by in-fusion cloning, to which fragment C was added to finally obtain pTight_DV3syn. (B) IFA for BHK-21 cells after transfection of DV3syn_WT plasmid. The DV3syn_WT clone plasmid was transfected into BHK-21 cells, together with plasmid pTet-off, and DENV-positive cells were monitored by IFA using anti-DENV NS3 antibody (green) and cell nuclei (blue). The images were captured using a fluorescence microscope. Bar, 100 μm. (C) Viral titers of DV3syn_WT released from transfected BHK-21 cells. The culture supernatant was collected at different days posttransfection and subjected to an FFU assay. The means and standard errors of the means (SEM) from three independent experiments are shown. #, titer below the limit of detection (<0.69 log_10_ FFU/mL).

For convenience of cloning the genome-length cDNA, we divided the viral genome cDNA into five fragments, A, B, C, D, and E, and constructed them into the pTight plasmid backbone (Fig. 2A). pTight vector is a low-copy-number plasmid modified from pBR322 to contain the seven repeated tetracycline response elements (7×TRE) upstream of the cytomegalovirus (CMV) promoter sequence (7×TRE-CMV) upstream and the hepatitis D virus ribozyme (HDVr) downstream of the cloned viral cDNA; this vector has been used for constructing infectious clones of DENV, ZIKV, and JEV (23, 31, 39). We first fused fragments A and B into A-B and fragments D and E into D-E, and then constructed them into the pTight vector (Fig. 2A). Then, fragment C was constructed by in-fusion cloning to obtain the genome-length cDNA clone. We designated this clone pTight-DV3syn_WT, for DENV-3 with wild-type (WT) and synthetic prM-E-NS1 regions (syn) (Fig. 2A and Table S3); the WT sequence defined here was used in order to distinguish it from the sequences with adaptive mutations identified subsequently.

### Efficient production of infectious DV3syn virus carrying adaptive mutations.

To test the ability of DV3syn_WT to produce infectious virus, we transfected BHK-21 cells with plasmid pTight-DV3syn_WT, together with plasmid pTet-off, which expressed the Tet-responsive transcriptional activator to activate cytomegalovirus promoter-controlled transcription (23, 31, 39). The culture supernatants were collected at 2, 4, 6, and 8 days posttransfection (dpt), and viral titers were determined by a focus-forming-unit (FFU) assay using BHK-21 cells. The results showed that DENV-positive cells were undetectable until 6 and 8 dpt (Fig. 2B); however, the viral titers were relatively low (2.2 × 10^2^ FFU/mL) (Fig. 2C). These results demonstrate that recombinant DV3syn_WT was able to produce infectious DENV after plasmid transfection of BHK-21 cells.

To increase the viral titer, we passaged the supernatant virus in BHK-21 cells, harvested supernatant 72 h postinfection, and inoculated it into fresh BHK-21 cells to make the next passage of the virus. The viral titer was examined when accelerated cell death or a cytopathic effect was observed, as previously described (23). The viral titer was increased to 1 × 10^4^ FFU/mL at passage 16. While virus passage was being performed, we sequenced the virus genome from passage 16 and identified an amino acid change, N1007D (nucleotide change, A3118G), in the NS1sequence. We engineered N1007D into DV3syn_WT to make DV3syn_1M. The titer of passaged DV3syn_WT virus was further increased to 6.7 × 10^4^ FFU/mL at passage 36, from which three mutations were identified, N131S (A486G) in prM, N1541S (A4716G) in NS3, and V2353A (T7152C) in NS4B (Table 1).

**TABLE 1:** Mutations identified in the recovered DV3syn_WT viruses after serial passage

Passage no.	Gene region	Nucleotide		Amino acid	
		Position	Change	Position	Change
16	NS1	3113	A-G	1007	Asn-Asp
36	prM	486	A-G	131	Asn-Ser
36	NS3	4716	A-G	1541	Asn-Ser
36	NS4B	7152	T-C	2353	Val-Ala

**FIG 3:**
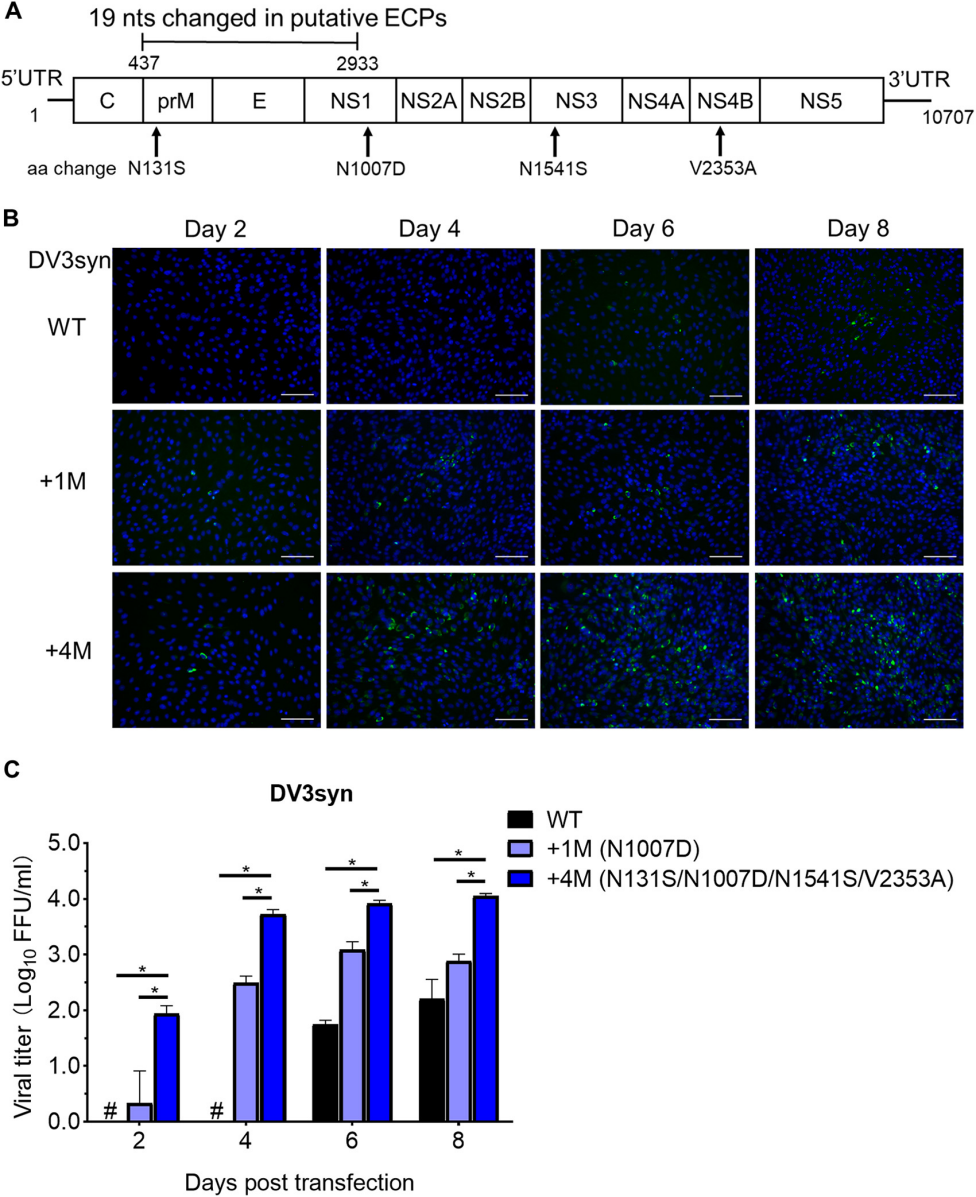
Adaptive mutations improved the virus production of DV3syn clone. (A) Diagram of DV3syn genome with four mutations (4M; N131S, N1007D, N1541S, and V2353A) identified from passage-recovered viruses (Table 1). (B) Immunostaining of BHK-21 cells transfected with DV3syn recombinant plasmids. DV3syn plasmids (DV3syn_WT, DV3syn_1M, and DV3syn_4M) were transfected, together with pTet-off plasmid, into BHK-21 cells, and DENV infection was detected by IFA. Bar, 100 μm. (C) Viral titers in culture supernatants. The supernatants were harvested from BHK-21 cells transfected with DV3syn_WT, DV3syn_1M, and DV3syn_4M and subjected to an FFU assay. The means and SEM from three independent experiments are shown. #, viral titer below the limit of detection (<0.69 log_10_ FFU/mL). Statistics analyses were performed using the multiple-comparison function in the two-way ANOVA of GraphPad (v 8.0). *, *P < *0.0001.

Next, we engineered these three mutations into DV3syn_1M to obtain DV3syn_N131S/N1007D/N1541S/V2353A, which we designated DV3syn_4M (Fig. 3A). DNA transfection of BHK-21 cells showed that DV3syn_1M (N1007D) had increased virus production compared to DV3syn_WT virus; DV3syn_WT started to be detectable from 6 dpt (similar to results shown in Fig. 2C), while DV3syn_1M was detectable at 2 dpt. The addition of four mutations (i.e., DV3syn_4M) further enhanced the viral titer, which was 27-, 17-, 6.5-, and 146-fold higher than the titer of DV3syn_1M at 2, 4, 6, and 8 dpt, respectively. The peak titer of DV3syn_4M was 1.1 × 10^4^ FFU/mL at 8 dpt (Fig. 3C). Sequence analysis of the recovered viruses revealed that the engineered mutations were maintained and no additional mutation was identified. Hence, we have developed an efficient infectious clone for the DENV-3 genotype III isolate.

### DV3syn_4M was genetically stable in transformed bacteria.

**FIG 4:**
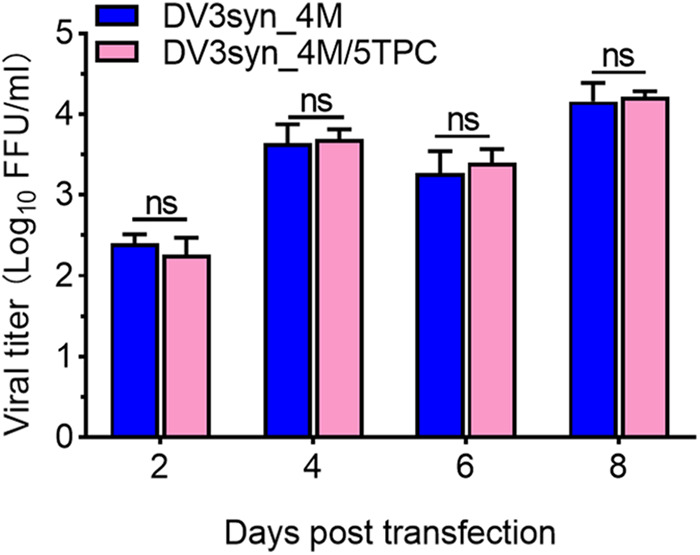
DV3syn_4M remained genetically stable in the bacteria after five transformation-purification cycles. DV3syn_4M plasmid was recovered from the fifth transformation-purification cycle (5TPC) and transfected into BHK-21 cells. The supernatant virus was collected, and the viral titer was determined by FFU assay. The means and SEM from three independent experiments are shown. Statistical analyses were performed using the multiple-comparison function in the two-way ANOVA of GraphPad (v 8.0). A *P* value of <0.05 was considered statistically significant. ns, no statistical significance.

During the assembly of the genome-length DENV-3 cDNA clone, we encountered difficulty with the instability of the prM-E-NS1 fragment in the bacteria. Previous studies also showed that *Flavivirus* cDNA exhibits instability in transformant recipient bacteria (39–41). To test the stability of the DV3syn_4M clone in bacteria, we repeated transformation-purification cycles (TPCs) in *Turbo* bacteria, as previously described (31). After 5 TPCs, the recovered plasmid, designated DV3syn_4M/5TPC, did not acquire additional mutations, insertions, or deletions. Transfection of DV3syn_4M/5TPC confirmed that DV3syn _4M/5TPC was as efficient as DV3syn_4M in virus production at 2, 4, 6, and 8 dpt, with a peak titer of 1.5 × 10^4^ FFU/mL at 8 dpt (Fig. 4). These results suggest that DV3syn_4M was genetically stable in recipient bacteria.

### The arylnaphthalene lignan C169-P1 inhibited DENV RNA replication, as determined by DENV-3 replicon screening.

**FIG 5:**
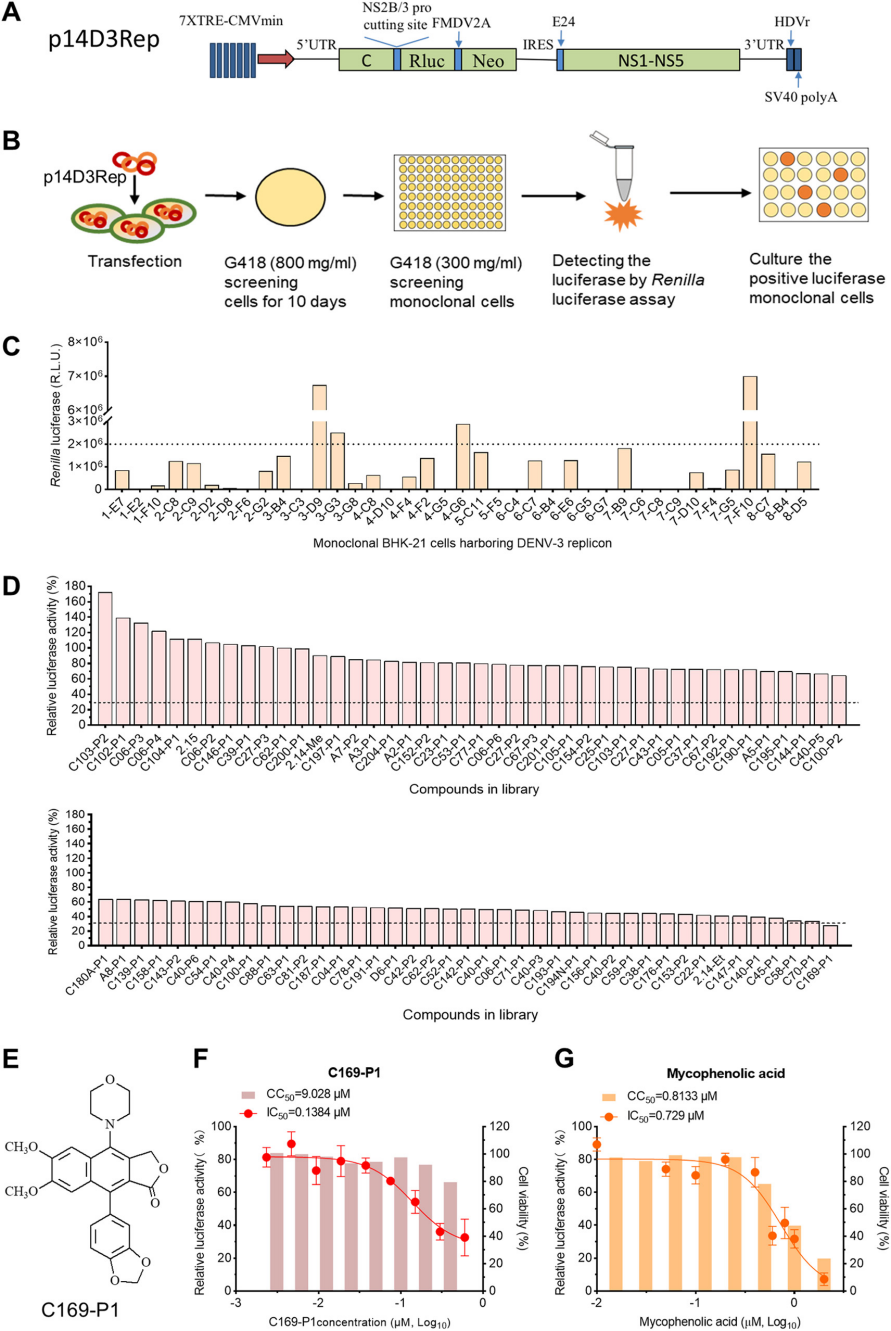
Screening of the compound library using BHK-21 cells harboring the DENV-3 subgenomic replicon, p14D3Rep. (A) Schematic diagram of the GZ14D3 subgenomic replicon construct, p14D3Rep. The construct was based on the DENV-2 replicon backbone, pCMV-DV2Rep (42), by placing the NS1-to-3'UTR region into the sequence of isolate GZ14D3. (B) Establishment of BHK-21 cell lines expressing p14D3Rep. (C) Selection of monoclonal BHK-21 cell lines expressing p14D3Rep with high *Renilla* luciferase activity, the signal reflecting the DENV RNA replication level. The p14D3Rep replicon cell line 3-D9 was selected for subsequent experiments. (D) Screening of antiviral compounds using p14D3Rep-replicating BHK-21 3-D9 cells. The 3-D9 cell line was used to screen 83 antiviral compounds, among which C169-P1 exhibited an inhibitory effect greater than 70%. (E) Chemical structure of C169-P1. (F) C169-P1 inhibited the RNA replication of the DENV-3 replicon. C169-P1 was administered at different concentrations to 3-D9 cells, and *Renilla* luciferase activity was determined. (G) Mycophenolic acid was used as an anti-DENV positive control.

To facilitate the high-throughput screening of the compound library, we also developed a DENV-3 subgenomic replicon containing the NS1-to-3' untranslated region (UTR) sequence of isolate GZ14D3, designated p14D3Rep (GenBank no. OR150112) (Fig. 5A), using the backbone of the DENV-2 replicon pCMV-DV2Rep reported previously (42). We transfected BHK-21 cells with the p14D3Rep replicon plasmid and selected 39 monoclonal cell lines in the presence of G418. Of these cell lines, 3-G3, 4-G6, 3-D9, and 7-F10 showed *Renilla* luciferase activity higher than that of other cell clones (≥2 × 10^6^ relative luciferase units [RLU]) (Fig. 5B and C).

3-D9 cells were used to screen a library containing 83 chemically synthesized derivatives of arylnaphthalene lignans, of which some were proved to inhibit HIV infection (35, 36). First, the 3-D9 cells seeded in 96-well plates were treated with two doses of 10-fold-diluted compounds starting in the range of 0.25 to 2.0 μM using 2% Dulbecco’s modified Eagle’s medium (DMEM) (Table S5); the starting concentrations of compounds were determined by a pilot study. The treatment lasted for 48 h, and the antiviral effect was determined by reduction of *Renilla* luciferase activity in the culture. Of 83 compounds, C169-P1 was considered a hit, as the *Renilla* luciferase activity dropped by 70% (Fig. 5D and E). We further characterized the inhibitory effect of C169-P1 on DENV-3 replication by serial dilutions and obtained a half-maximal inhibitory concentration (IC_50_) of 0.1384 μM for the compound, which had a 50% cytotoxic concentration (CC_50_) of 9.028 μM. The selectivity index (SI [CC_50_/IC_50_]) was 65.42 (Fig. 5F), higher than that of the positive control mycophenolic acid (SI = 1.12) (Fig. 5G). These results demonstrate that C169-P1 inhibited the RNA replication of DENV.

### C169-P1 inhibited the infections of the DENV-3 clone and other DENV serotypes.

**FIG 6:**
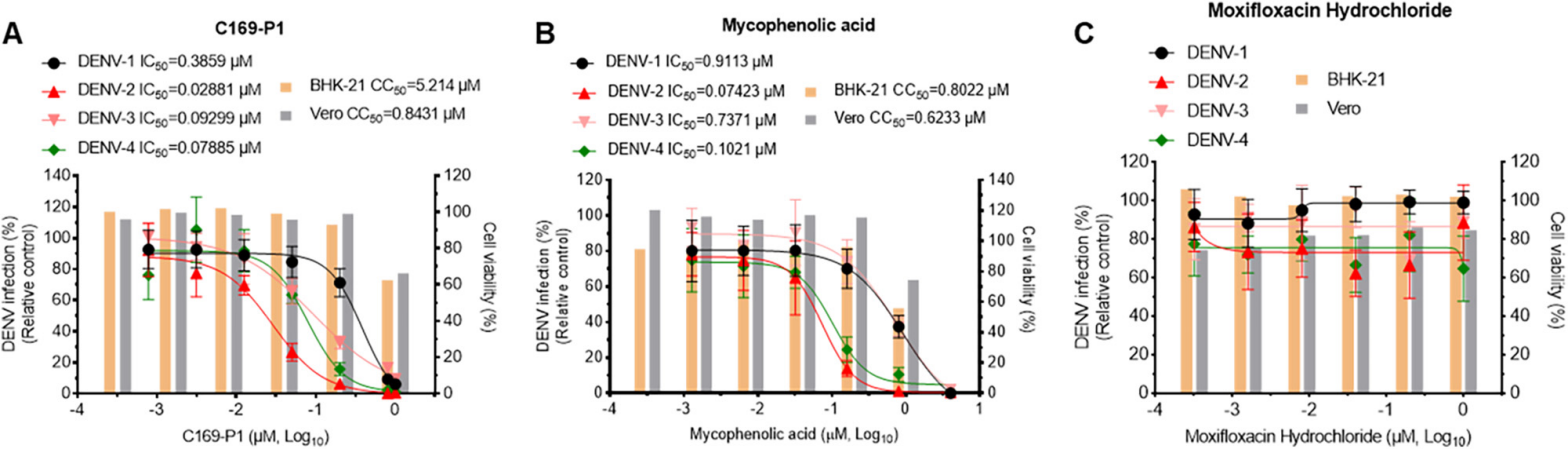
C169-P1 inhibited DENV-1 to -4 in a dose-dependent manner. BHK-21 cells were used for treatment of DENV-1 and DENV-3, while Vero cells were used for DENV-2 and DENV-4. A serial dilution of the respective drug was added to the culture infected with DENV and incubated for 48 h. Then, the cells were fixed and subjected to IFA to examine the reduction of DENV infection. (A) C169-P1; (B) mycophenolic acid; (C) moxifloxacin hydrochloride. The data are means and SEM from three independent experiments.

To explore whether C169-P1 had antiviral activity against DENV, we tested C169-P1 against infections with DENV-1 to -4, including the DENV-1 clone D19044_7M, which we developed recently (23), the DENV-2 clone 16681 (39), the DENV-3 clone DV3syn_4M (Fig. 3), and DENV-4 (43). Since DENV-1 and DENV-3 replicated better in BHK-2 cells, while DENV-2 and DENV-4 were more suitable for propagation in Vero cells, we performed antiviral treatment using two cell lines. Antiviral treatments were performed below the CC_50_s for C169-P1, with mycophenolic acid as the positive control and moxifloxacin hydrochloride as the negative control (Fig. 6). The results of antiviral treatment showed that C169-P1 inhibited DENV-1 to -4 in a dose-dependent manner, with IC_50_s and SIs of 0.3859 μM and 13.51 for DENV-1, 0.02881 μM and 29.26 for DENV-2, 0.09299 μM and 56.07 for DENV-3, and 0.07885 μM and 10.69 for DENV-4 (Fig. 6A). Mycophenolic acid inhibited the viruses of all serotypes; however, the IC_50_s and SIs varied significantly for testing in BHK-21 and Vero cells (Fig. 6B), while moxifloxacin hydrochloride did not exhibit any inhibitory activity for DENV and was well tolerated in both cell lines (Fig. 6C). Thus, we identified C169-P1 as a compound with antiviral activity against the four serotypes of DENV.

### C169-P1 also inhibited the internalization of the DENV life cycle.

**FIG 7:**
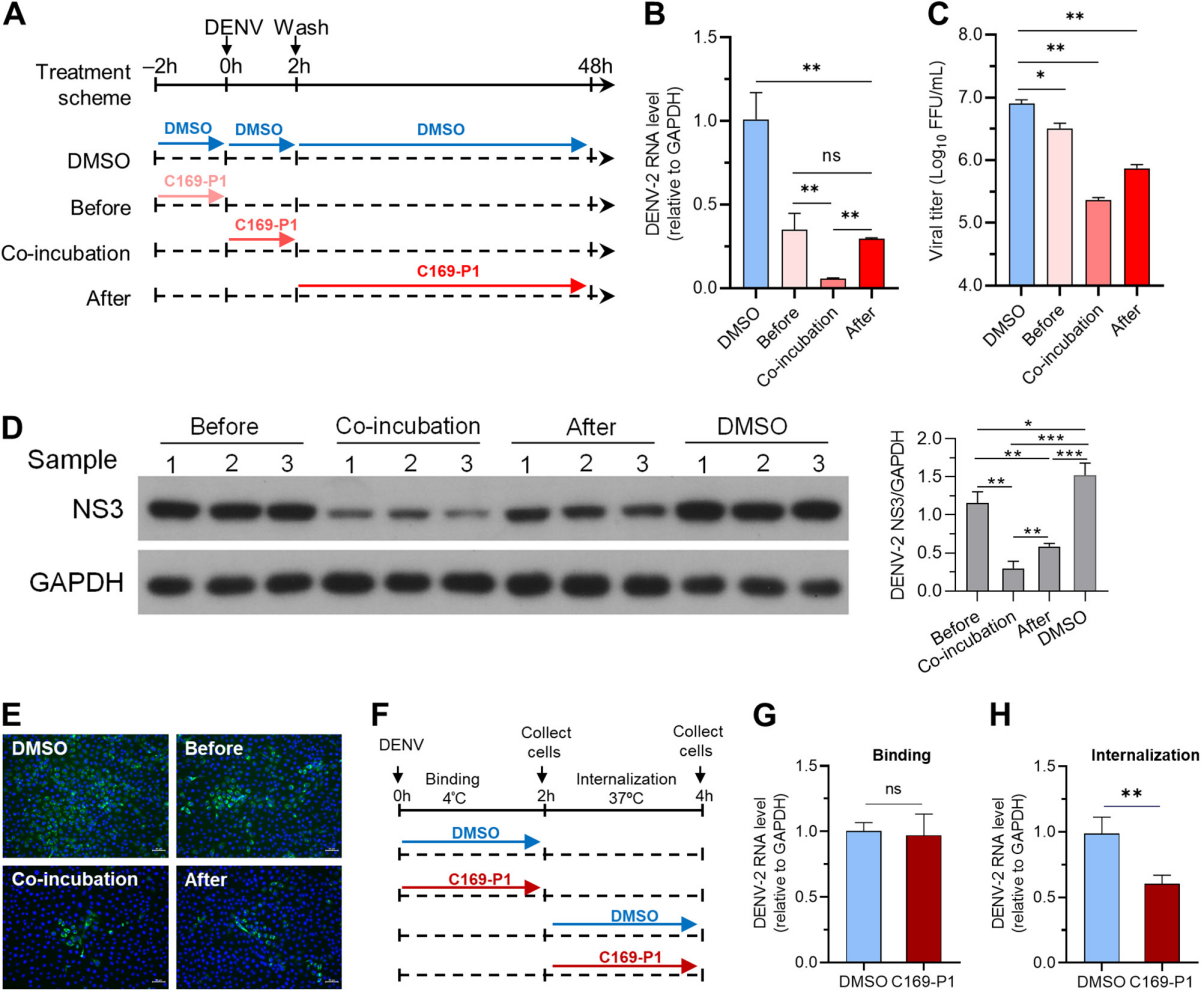
C169-P1 inhibited the internalization process of DENV cell entry. Vero cells were infected with DENV-2 clone 16681 (MOI = 1) at time point 0 h. C169-P1 treatment was administered at three different times: –2 h to 0 h (“before” group), 0 h to 2 h (“co-incubation” group), and 2 h to 48 h (“after” group). The control group received the solvent DMSO. The supernatant and cells were collected at 48 h for analysis. (A) Schematic view of time-of-drug addition experiment. (B) The DENV-2 RNA levels were determined at the 48 h time point, relative to GAPDH. (C) The viral titers of culture supernatant at 48 h were measured. (D) The intracellular viral NS3 protein at 48 h was detected by Western blotting, and results for three samples from one representative experiment out of three independent experiments are presented (left). Band density analysis was performed (right). (E) Vero cells were infected by the supernatant virus collected at 48 h from the treatment experiment that were shown in panel A. The viruses were diluted 100-fold, and DENV-infected cells were visualized by IFA for viral NS3 protein (green) and cell nuclei (blue). Images were captured using a fluorescence microscope. Bar, 50 μm. (F) Schematic diagram of the binding and internalization assay. (G and H) The RNA levels of DENV-2 binding and internalization were determined relative to the internal control GAPDH. Three independent experiments with three replicates per experiment were conducted, and the means and SEM are shown. ns, not significant; *, *P* < 0.05; **, *P* < 0.01; ***, *P* < 0.001.

To further investigate the mechanisms by which C169-P1 inhibited DENV infection, in addition to RNA replication, we determined which steps of the DENV life cycle were affected by performing time-of-drug addition experiments. As shown in Fig. 7A, Vero cells were infected with DENV-2 clone 16681 (multiplicity of infection [MOI] = 1; set as the 0 h time point) and left for 48 h. Treatment with C169-P1 (0.5 μM) was carried out at three time points: before incubation (−2 h to 0 h), during incubation (co-incubation) (0 h to 2 h), and after incubation (2 h to 48 h). A control group containing the solvent dimethyl sulfoxide (DMSO) was included in parallel (Fig. 7A). The cells and supernatant of each group were collected at 48 h for subsequent analysis. The results showed that the before-, during-, and after-incubation groups exhibited significantly lower levels of supernatant viral RNA (Fig. 7B), infectious virus titers (Fig. 7C), and intracellular viral NS3 proteins than the DMSO control group (Fig. 7D and E). Of these treatment groups, the before-incubation group exhibited reduced virus infection, while the co-incubation group showed the lowest level of virus infection; the after-incubation group had slightly higher levels than those observed in the co-incubation group. These data indicate that C169-P1 exerted a host cell-dependent antiviral effect, primarily targeting the viral entry step.

Viral entry could be divided into two stages, binding and internalization. To investigate the specific process targeted by C169-P1, we conducted a binding and internalization assay (44). The results demonstrated that C169-P1 inhibits the internalization process of DENV cell entry but not the binding stage (Fig. 7F to H).

## DISCUSSION

In this study, we constructed a DNA-launched genome-length infectious clone and a subgenomic replicon for DENV-3 and identified an arylnaphthalene lignan derivative with antiviral activity against DENV-1 to -4. The genome-length DV3syn cDNA was assembled after introduction of synonymous mutations to reduce the putative ECP activity predicted in the prM-E-NS region. The viral titer was increased by the introduction of four adaptive mutations (4M) identified from serially passaged viruses. The final infections clone, DVsyn_4M, was able to release infectious virus at 1.5 × 10^4^ to 6.7 × 10^4^ FFU/mL. Using the DENV-3 replicon-harboring cell line 3-D9, compound C169-P1 was selected and demonstrated to have an antiviral effect against DENV-1 to -4. Finally, we demonstrated that C169-P1 inhibited DENV RNA replication and cell entry. The reverse genetic systems of DENV-3 and the anti-DENV compound candidate will facilitate the study of this important human pathogen.

Although reverse genetics represents a valuable tool for the study of molecular virology, construction of a cDNA clone for flaviviruses has been difficult. The instability of cDNA in transformed bacteria remains an unresolved issue. Mutations, deletions, or insertions were frequently seen in the genome cDNA, mainly in the prM-E sequence. In most cases, transformant bacterial colonies could not be recovered. It is believed that viral cDNA may contain ECPs that initiate transcription of some proteins that cause toxicity to the transformed bacteria. However, the mechanisms underlying this phenomenon are unclear. Researchers have tried different approaches to construct infectious clones for flaviviruses, such as optimization of the vector plasmid (39), selection of special recipient bacteria (22, 45), inserting introns (40, 46), and reducing ECP activity by point mutations (29). For DENV-3 clones, several methods have been used, including fragment recombination in Saccharomyces cerevisiae (19), *in vitro* ligation of two fragments (26), and circular polymerase extension reaction (CPER) without bacterial transformation (27). Here, we adopted the approach of introducing synonymous mutations to reduce the ECP activity, as previous described (23, 29, 31). Beyond reducing ECP activity, we used a synthetic consensus prM-E-NS1 sequence of DENV-3, as both wild-type D191267 cDNA and the other DENV-3 isolate could not be cloned (data not shown). In addition, up to 16 ECPs were predicted, and 10 of them had scores of ≥0.9, higher than those predicted for the infectious clones, DENV-2 (29), JEV (29), and ZIKV (31, 38). Therefore, a consensus sequence of DENV-3 prM-E-NS1, carrying 19 synonymous mutations to reduce the ECP activity, was used for the cloning in this study. Finally, the genome-length cDNA clone, DV3syn, was assembled in the modified pTight (Fig. 2A). This optimization strategy could be prove advantageous in the construction of cDNA clones for other flaviviruses or other RNA viruses.

A great number of compounds with anti-flavivirus activity have been identified, but only a few have been further evaluated in preclinical trials, mainly due to the issues of efficacy, specificity, toxicity, or stability (47). To date, there is no drug approval for DENV infection. Cells harboring viral replicons represent a valuable approach for antiviral drug discovery in high-throughput library screening (48–51). To facilitate anti-DENV drug discovery, we also constructed a DENV-3 subgenomic replicon, p14D3Rep, using the sequence of isolate GZ14D3. The p14D3Rep was replication competent in BHK-21 cells. Using the p14D3Rep-harboring BHK-21 cell clone 3-D9, we screened out C169-P1 from a library of 83 aryl naphthalene lignans.

Plant extracts have demonstrated advantages in drug discovery due to the unique structures of certain natural compounds. Narasin was found to inhibit DENV replication through noncytotoxic mechanisms (49). Citrus flavanone naringenin and phthalazinone derivatives were found to have anti-DENV replication activity by using DENV replicon cells (50, 51). The flavonoid derivative ST081006 interferes with viral protein and/or RNA synthesis pathway (52). Arylnaphthalene lignan extracts were originally prepared from plants. Here, we identified an arylnaphthalene lignan derivative, C169-P1, as an inhibitor of the replication of DENV-3 p14D3Rep replicon (Fig. 5) as well as the internalization process of cell entry (Fig. 7). The functions of C169-P1 had enabled it to exhibit a potent antiviral effect against infection with DENV-1, -2, -3, and -4 (Fig. 6). The SI values of C169-P1 were in the range of 10.69 to 56.07, showing its potential for further development. We previously demonstrated that the arylnaphthalene lignans inhibited HIV replication (35, 36). The anti-DENV effect of C169-P1 described here expands the antiviral spectrum of arylnaphthalene lignans to include the family *Flaviviridae*. Thus, C169-P1 could be a candidate compound for future drug development to treat DENV infection.

In summary, we have constructed reverse genetic systems for DENV-3 isolates that have recently become prevalent in south China and demonstrated their utility for anti-DENV inhibitor discovery. The arylnaphthalene lignan compound candidate C169-P1 was identified as an inhibitor against infections with DENV-1, -2, -3, and -4 by impeding replication and cell entry. The reverse genetic systems and C169-P1 compound will permit future study of DENV-3 and anti-DENV drug development.

## MATERIALS AND METHODS

### Cell culture and DENV-infected serum.

C6/36 cells were provided by Ping Zhang (Sun Yat-sen University, China) and cultured in DMEM (Gibco/Thermo Fisher Scientific; 248988) with 10% fetal bovine serum (FBS; ExCell Bio; FSP500), 1% penicillin-streptomycin (HyClone; SV30010), and 1× nonessential amino acid solution (Gibco/Thermo Fisher Scientific; 11140050). BHK-21 and Vero cells were maintained in our laboratory and cultured in high-glucose DMEM with 10% FBS and 1% penicillin-streptomycin. The cells were incubated with 5% CO_2_ at 37°C. Cells were monitored routinely to exclude mycoplasma contamination (23). Two serum samples from DENV-3-infected individuals were collected; serum D191267 was collected during the 2019 epidemic in Qingyuan, Guangdong Province, while serum GZ14D3 was from the 2014 epidemic in Guangzhou, Guangdong Province. These sera were collected for the purpose of disease surveillance by Guangdong Provincial Center for Disease Prevention and Control, China.

### Virus stock and determination of viral genome sequence.

To generate virus stock, patient sera (D191267 and GZ14D3) were inoculated into C6/36 cells, and the supernatant was collected at 6 dpi. To determine the genome sequence of the virus stock or the recombinant viruses constructed in this study, total RNA was extracted from culture supernatant using the MagZol reagent (Magen; R4801), and viral cDNA was synthesized by reverse transcription using SuperScript III reverse transcriptase (Invitrogen; 18080044) (primers R5952* and R10623* [Table S1]). Four overlapping PCRs covering the ORF and partial 5′ UTR and 3′ UTR sequences were amplified (primers F54/R3055, F2836/R5952, F5812/R8854, and F8740/R10623 [Table S1]). These four PCR fragments were sequenced by Sanger sequencing and assembled to obtain the genome sequence spanning nt 54 to 10623 (GenBank no. OQ948473). The genomic cDNA sequence of virus stock was aligned to seven DENV-3 sequences (GenBank no. MN227697.1, MN227698.1, MN227699.1, MN227701.1, MN227702.1, MN227703.1, and MN964274.1), which were determined previously by Guangdong Provincial Center for Disease Prevention and Control, China.

### Phylogenetics analysis.

The ORF sequence of D191267 was phylogenetically analyzed with the selected sequences of DENV-1 to -4, including 19 strains of DENV-3 genotypes I to V and two sequences each from DENV-1, DENV-2, and DENV-4. Sequence alignment was performed with the neighbor-joining method in MEGA (Molecular Evolutionary Genetics Analysis, version 6; Kimura 2 parameter). The sequence homology between D191267 and the selected sequences was analyzed with BLAST (Basic Local Alignment Search Tool, version +2.13.0).

### The deduced consensus prM-E-NS1 sequence of DENV-3 and prediction of ECPs.

To construct a consensus sequence for the prM-E-NS1 region, 180 DENV-3 sequences containing complete ORFs were retrieved from GenBank. These sequences were collected from different continents: 5 strains were from Africa, 112 from Asia, 2 from Europe, 30 from North America, 27 from South America, and 4 from Oceania. A consensus sequence containing prM-E with a partial NS1 region (nt 437 to 2933; referred to here as prM-E-NS1) was deduced by the sequences from the same continent using Vector NTI software (alignments 1 to 6 in Fig. S1). Then, a DENV-3 consensus sequence was obtained by alignment of continental consensus sequences (alignment 7 in Fig. S1).

To predict the ECP existing in the prM-E-NS1 region, the DENV-3 consensus sequence, deduced from 180 DENV-3 sequences (see above), was analyzed by the online Berkeley Drosophila Genome Project (https://www.fruitfly.org/), as previously described (29, 31). A total of 19 sequences containing putative ECPs with transcription start sites were hits, with a score of ≥0.8. Then, the predicted ECP sites were mutated by synonymous substitutions to reduce the score to the level at which promoter activity is theoretically lost (normally ≤0.8) (Table S2). Since the prM-E-NS1 region (nt 437 to 2933) was a deduced consensus sequence and a synthetic sequence with ECP substitutions, we designated the assembled genome-length clone DV3syn (GenBank no. OQ948474).

### Construction of the genome-length DENV-3 cDNA clone DV3syn.

To assemble a genome-length DENV-3 cDNA clone, partial 5′ UTR (nt 1 to 53) and 3′ UTR (nt 10,624 to 10,707) sequences were synthesized and were identical to those of the other DENV-3 isolate, GZ2D3 (GenBank no. JN662391.1) (53); the synthetic terminal sequences are in the conserved regions of the DENV genome (54). Nucleotides 53 to 94 of the 5′ UTR are identical between GZ2D3 and D191267, while the other part of the 3′ UTR (nt 10268 to 10623) differs by 8 nt. For the convenience of cloning, we divided the viral genome cDNA into five fragments: A (nt 1 to 455; SacI), subdivided into A1 (nt 1 to 53) and A2 (nt 54–455), B (ECP-reduced fragment; nt 418 to 3055; BsrGI), C (nt 2914 to 5952; BsrGI-SacII), D (nt 5812 to 8854; SacII-KasI), and E (nt 8740 to 10707; KasI-RsrII), subdivided into E1 (nt 8740 to 10623) and E2 (nt 10624 to 10707). Plasmid vector pMD18-T was used for subcloning of PCR products (TaKaRa; 6011). A pTight vector modified from pBR322 was used as a vector plasmid for DENV cDNA cloning, as previously described (pTight-DENV-2 was originally provided by Andrew Yueh, National Health Research Institutes, Taiwan, China) (39). This vector has been used for cloning of flavivirus cDNA as previously described (23, 31, 39). The modified pTight vector contains seven repeated tetracycline response elements (7×TRE) upstream of the minimal cytomegalovirus (CMVmin) promoter sequence (7×TRE-CMVmin) and the antigenomic sequences of the HDVr (39, 42). The genome-length DENV-3 cDNA was cloned between CMVmin and HDVr; HDVr sequence ensures the generation of the correct cDNA clone 3′ end. Competent *Turbo* cells were prepared in our laboratory (originally from Hongying Chen, Northwest A&F University, China). All plasmids were confirmed by Sanger sequencing.

### Construction of a subgenomic DENV-3 replicon and selection of replicon-harboring BHK-21 cells.

The C6/36-derived GZ14D3 virus was passaged in Vero cells, and the supernatant was collected and subjected to viral RNA extraction. The nearly genome-length sequence was determined by Sanger sequencing or RT-PCR products (see above). To construct a subgenomic DENV-3 replicon, the NS1-to-3'UTR region of GZ14D3 (genome nt 2414 to 10707, including the last 82 nt from isolate GZ2D3 [JN662391.1]; GenBank no. OR150112) was cloned into the pCMV-DV2Rep by replacing the corresponding sequence of DENV-2 strain 16681, which was previously constructed based on the pTight vector (pCMV-DV2Rep was provided by Andrew Yueh, National Health Research Institutes, Taiwan, China) (42). To select BHK-21 cells harboring the GZ14D3 replicon, the replicon plasmid was transfected into BHK-21 cells using Lipofectamine 2000 (Invitrogen; 11668019). The transfected cells were selected with G418 (800 μg/mL) (Sigma; A1720) for 10 days. The selected monoclonal cells, which stably expressed DENV-3 replicons, were maintained in DMEM with 10% FBS, 1% penicillin-streptomycin, and 300 μg/mL of G418. The replication of the DENV-3 replicon was examined using a *Renilla* luciferase assay system (Promega; E2820) following the manufacturer’s instructions.

### Transformation and purification of plasmids.

Plasmid transformation and purification were performed as described in our previous study (23). In brief, plasmids were transformed into Turbo competent bacteria, streaked on agar plates, and cultured at 28°C for 24 h. Single colonies were picked randomly and cultured in 2× yeast extract tryptone broth (25 μg/mL) for 20 h, with 220 rotations per min, and plasmid DNA was extracted and purified with a HiPure plasmid minikit (Magen; P1003). The plasmids were confirmed by Sanger sequencing.

### Plasmid transfection, virus infection, and virus passage.

To generate DV3syn recombinant virus, the genome-length cDNA clone pTight-DV3syn (2 μg) and plasmid pTet-off (1 μg) were mixed and incubated in Opti-MEM containing the transfection reagent ExFect (Vazyme Biotech; T101) for 20 min. Then, the transfection mixture was transfected into BHK-21 cells grown in 6-well plates at 37°C for 6 h, the transfection medium was replaced with DMEM supplemented with 2% FBS, and the culture was cultured for 2 days. The culture supernatant was collected at days 2, 4, 6, and 8 or as otherwise stated, filtered (0.2 μm), and stored at −80°C for later use.

For virus infection, BHK-21 cells were incubated with transfection- or passage-recovered supernatant DV3syn virus for 2 h, and then the incubation medium was replaced with fresh medium. The infected culture supernatant was harvested at days 2, 4, 6, and 8 for later analysis.

To passage the virus, BHK-21 cells were inoculated with transfection-derived supernatant virus in DMEM with 2% FBS for 72 h. Culture supernatant was collected, filtered (0.2 μm), and designated the first-passage virus. To serially passage the virus, the first-passage virus (1 mL) was inoculated into BHK-21 cells grown in 6-well plates for 72 h, and the culture supernatant was collected and referred to as the second-passage virus; subsequently, more passages of virus were generated.

### IFA for virus titration.

The viral infectivity titer was determined by indirect immunofluorescence focus assay (IFA) using BHK-21 or Vero cells, as previously described (55, 56), and results are expressed as FFU per milliliter (FFU/mL). Briefly, BHK-21 cells, or Vero cells when appropriate, were inoculated with a 10-fold serial dilutions (from 10° to 10^6^ dilutions) for 2 h; then, the virus inoculum was removed, and the cells were washed once with DMEM and cultured with DMEM containing 2% FBS for 48 h. The cells were fixed with methanol (−20°C) for 15 min and subsequently incubated with anti-DENV NS3 antibody (1:2,000 dilution; GeneTex; GTX124252) at 4°C overnight. To visualize DENV-infected cells, the secondary antibody Alexa Fluor 488 goat anti-mouse IgG (H+L) (1:1,000 dilution; Invitrogen; A32731) and dye Hoechst 33258 (1:1,000 dilution; Invitrogen; H3569) were added at room temperature and incubated for 1 h. The DENV-positive cells were enumerated manually under a fluorescence microscope (Leica Microsystems; DMI 4000B).

### Cell viability.

Cell viability was measured as previously described (23). Briefly, Vero cells or BHK-21 cells were seeded into 96-well plates (4 × 10^3^ cells/well) 16 to 20 h before compound treatment. The cells were incubated with different concentrations of drugs for 48 h, and 10 μL of Cell Counting Kit-8 solution (CCK8; Dojindo; CK04) was added to each well with gentle shaking. The plate was incubated at 37°C with 5% CO_2_ for 90 min. The optical density at 450 nm (OD_450_) was measured with a microplate reader (BioTek ELx800).

### Antiviral treatment of the DENV-3 replicon and DENV-1 to -4.

For treatment of the DENV-3 replicon, BHK-21 cells harboring the DENV-3 replicon were seeded in 96-well plate at 4 × 10^3^ cells/well and incubated for 16 to 20 h, and then the cells were incubated with different concentration of drugs for 48 h, as appropriate. The cells were harvested and lysed for 15 min at room temperature using *Renilla* luciferase lysis buffer (Promega; E2820). The relative luminescence (in RLU) was measured with a *Renilla* luciferase assay system (Promega; E2820).

For testing antivirals against DENV infection, we obtained an arylnaphthalene lignan compound library from Gihon Biotech Limited (Hong Kong, China) and dissolved each compound to 10 mM using DMSO; compounds were then aliquoted and stored at −80°C. Mycophenolic acid (MPA; TargetMol; T1335) was treated in parallel as a positive drug control. Moxifloxacin hydrochloride (Tianjin Chasesun Pharmaceutical; H20203057) was prepared with DMSO as negative control. Compounds were diluted to different concentrations (below the cell cytotoxicity concentration) using DMEM with 2% FBS. BHK-21 cells (4 × 10^3^ cells/well) were used for DENV-1 and DENV-3 infections, whereas Vero cells (5 × 10^3^ cells/well) were used for DENV-2 and DENV-4 infections. The cells were seeded into 96-well plates, cultured for 16 to 20 h, and then infected with DENV (MOI = 0.5) using DMEM containing different concentrations of drugs. At 2 h postinfection, the medium was removed, and the cells were washed once with DMEM and incubated with fresh DMEM with 2% FBS containing drug. After 48 h, the cells were fixed, and the percentage of DENV-positive cells was determined by IFA.

### Time-of-drug addition assay.

To evaluate the effect of candidate compound C169-P1 on the steps of DENV life cycle, a time-of-drug addition experiment was performed. Vero cells at 70 to 80% confluence were infected with DENV-2 clone 16681 (set as 0 h) for 2 h (MOI = 1), washed with phosphate-buffered saline (PBS) 3 times, and then incubated with DMEM with 2% FBS for 48 h. Four drug addition experiments were performed: (i) C169-P1 (0.5 μM) was added to the cells 2 h before virus infection and incubated for 2 h, i.e., −2 h to 0 h (before-incubation group); (ii) C169-P1 (0.5 μM) was added at 0 h, simultaneously with virus addition, and incubated for 2 h, i.e., 0 h to 2 h (during-incubation [co-incubation] group); (iii) C169-P1 (0.5 μM) was added at 2 h after virus infection and maintained to 48 h (after-incubation group); (iv) cells were treated with 0.2% DMSO in parallel from −2 h to 48 h. The cells and supernatant collected from groups 1 to 4 were analyzed for viral RNA by quantitative RT-PCR (qRT-PCR), NS3 protein was analyzed by Western blotting, and supernatant virus titers were determined by an FFU assay.

### Viral binding and internalization assays.

The viral binding and internalization assays were performed as previously described (44). Vero cells were incubated with DENV-2 (MOI = 1) for 2 h at 4°C and then washed three times with cold PBS to remove unbound viral particles. For the binding assay, the cells were directly subjected to RNA extraction, and the relative level of DENV-2 RNA was determined by qRT-PCR. For internalization assays, the cells were shifted to 37°C for 2 h. Then, the cells were treated with proteinase K (1 mg/mL) for 3 min at room temperature, and the cells were collected by two rounds of centrifugation to remove the extracellular virus particles. The cell pellets were resuspended in PBS and subjected to RNA extraction and determination of DENV-2 RNA levels.

### Western blotting.

Cells were lysed with lysis buffer and subjected to SDS-PAGE. The proteins were transferred to 0.2 μm polyvinylidene difluoride (PVDF) membranes (Bio-Rad; 1620177), blocked with 5% skim milk, and incubated at 4°C overnight with anti-DENV NS3 antibody (1:2,000; GeneTex; GTX124252) and horseradish peroxidase (HRP)-conjugated glyceraldehyde-3-phosphate dehydrogenase (GAPDH) monoclonal antibody (1:5,000; Proteintech; HRP-60004). After three washes with Tris-buffered saline supplemented with 0.1% Tween (TBST) (10 min per wash), goat anti-rabbit IgG (H+L) HRP-conjugated secondary antibody (1:5,000; Beijing Ray Antibody Biotech; RM-3002) was added for 1 h at room temperature. After three washes with TBST (10 min per wash), the NS3 and GAPDH bands were visualized with the Immobilon Western chemiluminescent HRP substrate (Proteintech; PK10001).

### Relative quantitative analysis of DENV-2 RNA.

Total RNA was isolated with MagZol reagent (Magen; R4801) following the manufacturer’s instructions. Viral cDNA was generated with HiScript II Q RT SuperMix (Vazyme Biotech; R222). Quantitative RT-qPCR for DENV-2 RNA was performed using the MagicSYBR mixture (CWBio, CW3008) and primers (5′ to 3′; DENV2-F, TTGAGTAAACTATGCTGCCTGTAGCTC, and DENV2-R, GAGACAGCAGGATCTCTGGTCTTTC) at 95°C for 3 min, 37 cycles of 95°C for 10 s, and 60°C for 30 s. The levels of viral RNA were normalized to the internal control GAPDH (primers GAPDH-F [TCAATGGAAGCCCCATCAC] and GAPDH-R [CATGACAAACATAGGGGCGTC]) and presented as fold change relative to the control cells.

### Data processing.

The data acquired were processed and plotted by GraphPad Prism (version 8.0). Statistical analysis was performed using the multiple-comparison function of two-way analysis of variance (ANOVA) in GraphPad Prism (version 8.0).

### Data availability.

The sequence covering nt 54 to 10623 of the virus genome has been deposited in GenBank under accession no. OQ948473. The assembled genome-length clone DV3syn has been deposited in GenBank under accession no. OQ948474. The NS1-to-NS5 region of p14D3Rep has been deposited in GenBank under accession no. OR150112.
