# Supplementary material for: Identification of an Arylnaphthalene Lignan Derivative as an Inhibitor against Dengue Virus Serotypes 1 to 4 (DENV-1 to -4) Using a Newly Developed DENV-3 Infectious Clone and Replicon
Source: Microbiol Spectr. 2023 Jun 28;11(4):e00423-23. doi: 10.1128/spectrum.00423-23 (PMC10434217; doi:10.1128/spectrum.00423-23)
Supplement: Supplemental file 1 — Supplemental material. Download spectrum.00423-23-s0001.pdf, PDF file, 11.5 MB [file spectrum.00423-23-s0001.pdf]

Supplementary Figure 1. Alignments of 180 DENV-3 sequences retrieved from GenBank.

1. DENV-3 sequences from Africa

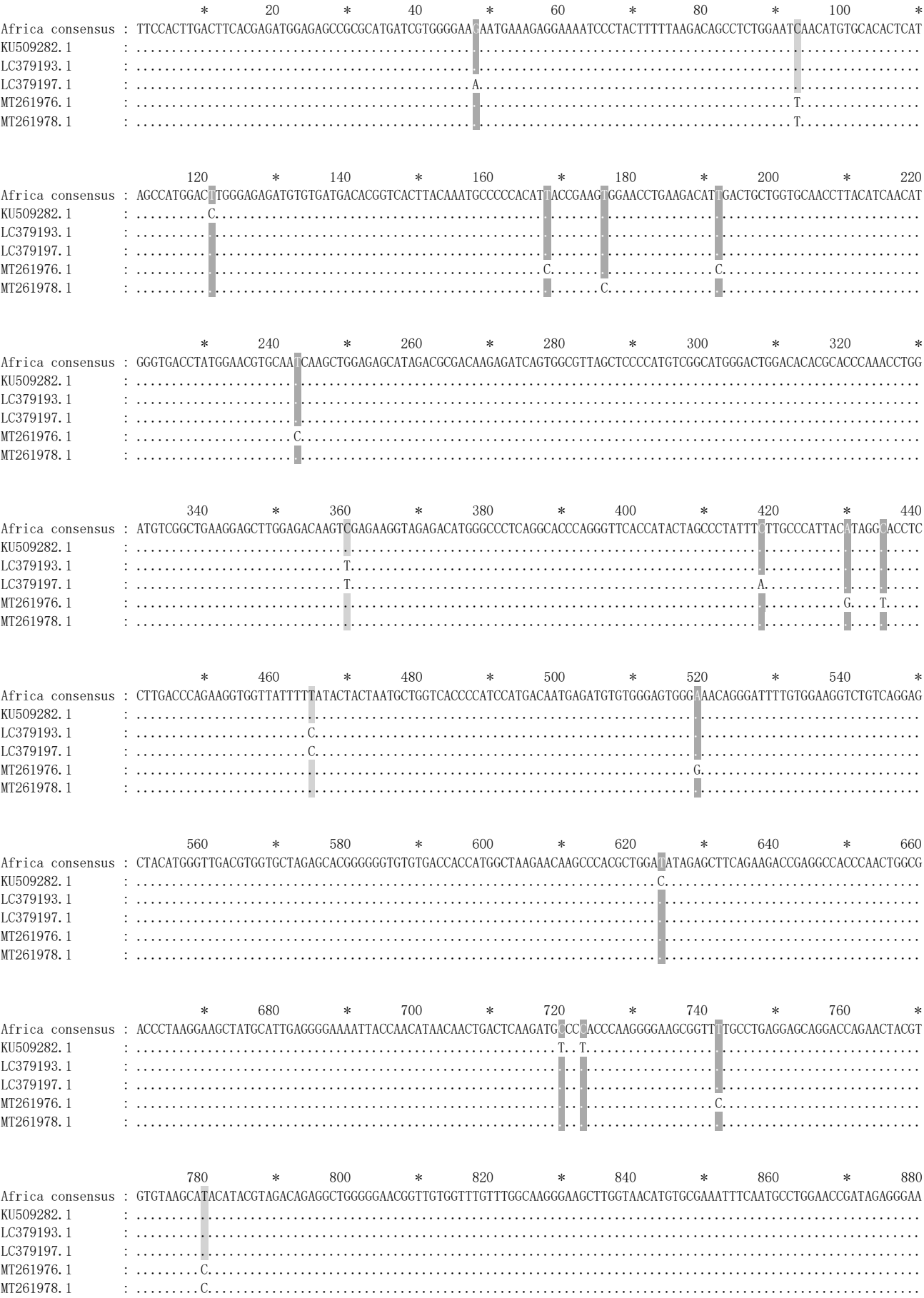

LC379197.1 : .....
MT261976.1 : .....T.....C.....
MT261978.1 : .....C.....

1880 * 1900 * 1920 * 1940 * 1960 * 1980
Africa consensus : GTGTTCTCTTGACTTGGATAGGGTTGAATTCAAAAACACATCCATGTCATTTTCATGCATTGCGATAGGAATCATCACACTCTATCTGGGAGCAGTGGTACAAGCTGAC
KU509282.1 : .....
LC379193.1 : .....T.....
LC379197.1 : .....T.....
MT261976.1 : .....A.....
MT261978.1 : .....A.....

* 2000 * 2020 * 2040 * 2060 * 2080 *
Africa consensus : ATGGGGTGTGTCATAAACTGGAAAGGCAAAGAACTTAAATGCGGAAGTGAATTTTCGTACCAACGAGGTCCATACCTGGACAGAGCAGTACAAATCCAAGCGGATTC
KU509282.1 : .....
LC379193.1 : .....
LC379197.1 : .....
MT261976.1 : .....C.....
MT261978.1 : .....T.....C.....

2100 * 2120 * 2140 * 2160 * 2180 * 2200
Africa consensus : CCCAAAGATTGGCGACAGCTATTGCAGGCGCTTGGGAAAAATGGAGTGTGCGGAATTAGGTCAACAACCAGAATGGAGAATCTTTGTGGAAGCAGATAGCCAATGAAC
KU509282.1 : .....G.....
LC379193.1 : .....T.....
LC379197.1 : .....
MT261976.1 : .....
MT261978.1 : .....

* 2220 * 2240 * 2260 * 2280 * 2300 *
Africa consensus : TGAACATACATATTATGGGAAAAATAATTAATAAACGGTAGTTGTGGGCGACACAATTGGGGTCTTGAGCAAGGAAAAAGAACACTAACACCACAACCCATGGAGCTA
KU509282.1 : .....
LC379193.1 : .....
LC379197.1 : .....
MT261976.1 : .....T.....G.....
MT261978.1 : .....T.....

2320 * 2340 * 2360 * 2380 * 2400 * 2420
Africa consensus : AAATACTCATGAAAAACGTGGGGAAAGGCAAAAATAGTGACAGCTGAAACACAAAATTCCTCTTTCATATAGACGGGCCAAACACACCGGAGTGTCCAAAGTGCCTCAAG
KU509282.1 : .....T.....
LC379193.1 : .....
LC379197.1 : .....
MT261976.1 : .....T.....G.....
MT261978.1 : .....

* 2440 * 2460 * 2480 *
Africa consensus : AGCATGGAATGTGTGGGAGGTGGAAGATTACGGGTTCGGAGTCTTCACAACCAACATATGGCTGAAACTCCGAGAGG
KU509282.1 : .....
LC379193.1 : .....
LC379197.1 : .....
MT261976.1 : .....
MT261978.1 : .....

2. DENV-3 sequences from Asia

[illegible]

[illegible]

[illegible]

[illegible]

MN922041.1	:	.....G.....A.....TT.....A.....
MN964274.1	:	.....T.....C.....
NC_001475.2	:	.....C.....

		*	460	*	480	*	500	*	520	*	540	*		
Asia consensus	:	CTTGA	CCCAAAAGGGT	ATTTTATACTACT	ATGCTGT	ACCCCATCCATG	CATGAG	TGTGT	GGATAGG	AACAG	GATTT	GTGGAA	GTGTGTC	GGAG
AB189127.1	:	.....	.....C.....	.....C.....	.....	.....T.....	.....	.....	.....	.....	.....	.....	.....C.....	.....A.....
AB214882.1	:	.....	.....C.....	.....C.....	.....G.....	.....T.....	.....	.....	.....	.....	.....	.....	.....C.....	.....A.....
AF317645.1	:	.....	.....	.....C.....	.....T.....	.....T.....	.....	.....	.....	.....	.....	.....	.....C.....	.....A.....
AY099336.1	:	.....C.....	.....G.....	.....C.....	.....T.....	.....T.....	.....	.....	.....	.....	.....	.....	.....G.....	.....A.....
AY496873.2	:	.....C.....	.....	.....G.....	.....	.....	.....T.....	.....G.....	.....T.....	.....C.....	.....	.....	.....	.....G.....
AY496877.2	:	.....C.....	.....	.....G.....	.....	.....	.....T.....	.....G.....	.....T.....	.....C.....	.....	.....	.....	.....G.....
AY496879.2	:	.....	.....	.....A.....	.....C.....	.....T.....	.....	.....G.....	.....	.....G.....	.....	.....	.....	.....C.....
AY648961.1	:	.....	.....	.....C.....	.....	.....	.....	.....	.....	.....C.....	.....	.....	.....	.....C.....
AY676351.1	:	.....C.....	.....	.....	.....	.....	.....T.....	.....	.....C.....	.....	.....G.....	.....	.....	.....G.....
AY766104.1	:	TC.....	A.....	.....	.....	.....	.....	.....	.....C.....	.....	.....	.....	.....	.....G.....
AY770511.2	:	.....	.....	.....G.....	.....	.....	.....	.....	.....	.....G.....	.....	.....	.....	.....
AY776329.1	:	.....	.....	.....C.....	.....	.....	.....	.....	.....	.....	.....	.....	.....	.....C.....
AY858038.2	:	.....	.....A.....	.....	.....C.....	.....G.....	.....	.....T.....	.....	.....	.....	.....	.....	.....C.....
AY858046.2	:	.....	.....	.....C.....	.....C.....	.....	.....	.....	.....	.....	.....	.....	.....	.....C.....
AY923865.1	:	.....C.....	.....	.....	.....	.....	.....	.....T.....	.....	.....C.....	.....	.....	.....	.....
DQ401689.1	:	.....C.....	.....	.....	.....G.....	.....	.....	.....T.....	.....G.....	.....T.....	.....C.....	.....	.....	.....G.....
DQ401694.1	:	.....	.....	.....C.....	.....	.....	.....	.....	.....	.....	.....	.....	.....	.....C.....
DQ401695.1	:	.....	.....	.....C.....	.....	.....	.....	.....	.....	.....	.....	.....	.....	.....C.....
DQ675522.1	:	.....C.....	.....	.....	.....T.....	.....	.....	.....	.....	.....C.....	.....	.....	.....	.....C.....
DQ675531.1	:	.....C.....	.....	.....	.....T.....	.....	.....	.....	.....	.....C.....	.....	.....	.....	.....C.....
DQ863638.1	:	.....	.....	.....	.....	.....A.....	.....	.....T.....	.....G.....	.....	.....C.....	.....	.....	.....A.....
EU081186.1	:	.....C.....	.....	.....G.....	.....	.....C.....	.....	.....T.....	.....	.....	.....	.....	.....	.....G.....
EU081203.1	:	.....C.....	.....	.....G.....	.....	.....C.....	.....	.....T.....	.....	.....	.....	.....	.....	.....G.....
EU081215.1	:	.....C.....	.....	.....G.....	.....	.....C.....	.....	.....T.....	.....	.....	.....	.....	.....	.....G.....
EU367962.1	:	.....C.....	.....	.....	.....	.....	.....	.....T.....	.....	.....C.....	.....	.....	.....	.....
FJ461329.1	:	.....C.....	.....	.....	.....	.....	.....	.....T.....	.....	.....C.....	.....	.....	.....	.....
FJ461338.1	:	.....C.....	.....	.....	.....	.....	.....	.....T.....	.....	.....C.....	.....	.....	.....	.....
FJ644564.1	:	.....	.....G.....	.....	.....	.....	.....	.....	.....	.....	.....	.....G.....	.....	.....
FJ744738.1	:	.....C.....	.....	.....	.....	.....	.....	.....T.....	.....	.....	.....	.....G.....	.....	.....G.....
FJ810414.1	:	.....C.....	.....	.....	.....	.....	.....	.....T.....	.....	.....	.....	.....G.....	.....	.....G.....
GQ199889.1	:	.....	.....	.....	.....	.....A.....	.....	.....T.....	.....G.....	.....	.....C.....	.....	.....	.....A.....
GQ466079.1	:	.....	.....	.....G.....	.....	.....	.....	.....	.....	.....	.....	.....G.....	.....	.....
GQ868571.1	:	.....	.....	.....G.....	.....C.....	.....	.....	.....	.....	.....	.....	.....	.....	.....A.....
GQ868575.1	:	.....	.....	.....G.....	.....C.....	.....	.....	.....	.....	.....	.....	.....	.....	.....A.....
GU131909.1	:	.....C.....	.....	.....	.....	.....	.....	.....T.....	.....	.....	.....C.....	.....	.....	.....

QQ466079.1	:	.	.	.	.	A.	.	G.	.	.	.	.	.	.	.	.	T.	.	.	.
QO868571.1	:	.	.	.	.	A.	.	G.	.	.	.	.	.	.	.	.	T.	T.	.	.
QO868575.1	:	.	.	.	.	.	.	G.	.	.	.	.	.	.	.	.	T.	T.	.	.
GU131909.1	:	.	.	.	T.	.	.	.	.	.	.	.	G.	T.	.	.	.	.	.	
GU131915.1	:	C.	.	.	T.	.	.	.	.	.	.	.	G.	T.	.	.	.	.	.	
GU131936.1	:	.	.	.	T.	.	.	.	C.	.	.	.	G.	T.	.	.	.	.	.	
GU131944.1	:	.	.	.	T.	.	.	.	.	.	.	.	G.	T.	.	.	.	.	.	
GU131954.1	:	.	.	.	.	.	.	G.	.	.	.	.	.	T.	T.	.	.	.	.	
GU363549.1	:	.	A.	.	.	A.	.	G.	.	.	.	.	.	.	.	.	T.	.	.	
HG316484.1	:	.	.	.	T.	.	.	.	.	.	.	.	.	T.	.	.	.	.	.	
JF295012.1	:	.	.	.	T.	.	.	.	.	.	.	.	G.	T.	.	.	.	.	.	
JN368477.1	:	.	.	.	T.	.	.	.	.	.	.	.	G.	T.	.	.	.	.	.	
JN662391.1	:	.	A.	.	.	A.	.	G.	.	.	.	.	.	.	.	T.	.	.	.	
JQ045687.1	:	.	.	.	T.	T.	.	.	.	T.	.	.	.	T.	.	.	.	A.	.	
JQ045688.1	:	.	.	.	T.	T.	.	.	.	T.	.	.	.	T.	.	.	.	A.	.	
JQ045689.1	:	.	.	.	T.	T.	.	.	.	T.	.	.	.	T.	T.	.	.	.	.	
JQ045690.1	:	.	.	.	T.	T.	.	.	.	T.	.	.	.	T.	T.	.	.	.	.	
JQ045691.1	:	.	.	.	T.	T.	.	.	.	T.	.	.	.	T.	.	.	.	A.	.	
JQ045692.1	:	.	.	.	T.	T.	.	.	.	T.	.	.	.	T.	.	.	.	A.	.	
JQ045693.1	:	.	.	.	T.	T.	.	.	.	T.	.	.	.	T.	.	.	.	A.	.	
JQ045694.1	:	.	.	.	T.	T.	.	.	.	T.	.	.	.	T.	.	.	.	.	.	
JQ045695.1	:	.	.	.	T.	T.	.	.	.	T.	.	.	.	T.	.	.	.	.	.	
JQ411814.1	:	.	.	.	.	.	.	G.	.	T.	.	.	.	.	.	.	T.	.	.	
JQ922555.1	:	.	.	.	.	.	.	.	.	T.	.	.	.	.	.	.	T.	.	.	
JQ922557.1	:	.	.	.	.	A.	.	G.	.	.	.	.	.	.	.	.	T.	.	.	
KC261634.1	:	.	.	.	T.	.	.	.	.	.	.	.	G.	T.	.	.	.	.	.	
KC762681.1	:	.	.	.	T.	.	.	.	.	T.	.	.	.	.	A.	T.	C.	.	.	
KC762691.1	:	.	A.	.	.	A.	.	.	.	T.	.	.	.	.	.	C.	.	.	A.	
KF041257.1	:	.	.	.	.	A.	.	G.	.	.	.	.	.	.	T.	.	.	.	.	
KF954949.1	:	.	.	.	.	A.	.	G.	.	.	.	.	.	.	T.	.	.	.	.	
KF955332.1	:	.	.	.	T.	A.	.	.	.	.	.	.	.	T.	.	.	.	.	.	
KF955333.1	:	.	.	.	T.	.	.	.	.	.	.	G.	T.	.	.	.	.	.	.	
KF955474.1	:	.	.	.	.	.	.	G.	.	T.	.	.	.	.	T.	.	.	.	.	
KF955476.1	:	.	.	.	.	.	.	.	.	.	.	.	.	.	.	.	.	.	.	
KF955507.1	:	.	.	.	T.	.	.	.	.	.	G.	T.	.	.	.	.	.	.	.	
KJ622191.1	:	.	A.	.	T.	.	.	.	.	.	.	.	.	.	.	.	T.	.	.	
KJ622195.1	:	.	A.	.	T.	.	.	.	.	.	.	.	.	.	.	.	T.	.	.	
KJ737430.1	:	.	.	.	T.	.	.	.	.	.	.	.	.	T.	.	.	.	.	.	
KP406805.1	:	.	.	.	.	.	.	.	.	T.	.	.	.	.	.	.	.	.	.	
KR296744.1	:	.	A.	.	T.	.	.	.	.	.	.	.	.	.	.	.	T.	.	.	
KT424097.1	:	.	.	.	T.	.	.	.	.	.	.	.	.	.	.	.	T.	.	.	
KU050695.1	:	C.	.	.	.	.	.	.	.	T.	.	.	.	.	.	.	V.			

MN922036.1 : .....A.....G.....T.....
MN922041.1 : .....A.....A.....T.....C.....A
MN964274.1 : .....A.....G.....A.....T.....
NC_001475.2 : .....G.....T.....T.....

Asia consensus : ACCCTAAGCA*GCTATG*ATGAGGGGAAA*ATTACCAACATTACACTGACTCAA*GTGTCTTACCAAGGGGAAGC*ATT*TTACCTGA*GA*CA*GA*CA*AA*CTACGT
AB189127.1 : .....A.....C.....G.G.....C.....C.C.....C.....
AB214882.1 : .....A.....C.....G.G.....C.....C.C.....
AF317645.1 : .....G.....C.....A.....C.....
AY099336.1 : .....G.....A.....G.....G.....
AY496873.2 : .....T.....T.....G.....C.....
AY496877.2 : .....T.....T.....G.....C.....
AY496879.2 : .....A.A.....G.....C.....C.C.....A.....
AY648961.1 : .....A.....T.....G.....C.....C.C.....C.....
AY676351.1 : .....T.....G.....
AY766104.1 : .....T.G.....T.....G.....C.....
AY770511.2 : .....G.....G.....A.....G.....G.....
AY776329.1 : .....A.....G.....C.....C.....C.....
AY858038.2 : .....A.....G.....C.....C.C.....
AY858046.2 : .....A.....G.....C.....G.G.....C.C.....T.....C.....
AY923865.1 : .....T.....G.....G.....
DQ401689.1 : .....C.....T.....G.....C.....
DQ401694.1 : .....A.....G.....C.....C.C.....A.....
DQ401695.1 : .....A.....G.....C.....C.C.....A.....
DQ675522.1 : .....T.....C.....
DQ675531.1 : .....T.....C.....
DQ863638.1 : .....T.....G.....
EU081186.1 : .....G.....A.....AG.....G.....
EU081203.1 : .....G.....A.....AG.....G.....
EU081215.1 : .....G.....A.....AG.....G.....
EU367962.1 : .....T.....C.....C.....
FJ461329.1 : .....T.....G.....C.....
FJ461338.1 : .....T.....C.....A.....
FJ644564.1 : .....G.....A.....G.....G.....
FJ744738.1 : .....T.....G.....
FJ810414.1 : .....T.....G.....
GQ199889.1 : .....T.....G.....A.....C.....G.....
GQ466079.1 : .....G.....A.....G.....G.....
GQ868571.1 : .....G.....C.....A.....G.....G.....
GQ868575.1 : .....G.....C.....A.....G.....G.....
GU131909.1 : .....T.....C.....C.....C.....
GU131915.1 : .....T.....C.....C.....
GU131936.1 : .....T.....C.....C.....
GU131944.1 : .....T.....C.....C.....
GU131954.1 : .....G.....C.....A.....G.....G.....
GU363549.1 : .....G.....A.....C.....G.....G.....
HG316484.1 : .....C.....C.....G.....C.....
JF295012.1 : .....T.....C.....C.....
JN368477.1 : .....T.....C.....C.....
JN662391.1 : .....G.....A.....C.....C.....G.....G.....
JQ045687.1 : .....T.....C.....A.....
JQ045688.1 : .....T.....C.....A.....
JQ045689.1 : .....T.....G.....C.....A.....
JQ045690.1 : .....T.....G.....C.....A.....
JQ045691.1 : .....T.....G.....C.....
JQ045692.1 : .....T.....G.....C.....
JQ045693.1 : .....T.....G.....C.....
JQ045694.1 : .....T.....C.....T.....
JQ045695.1 : .....T.....C.....T.....
JQ411814.1 : .....G.....A.....G.....G.....
JQ922555.1 : .....G.....G.....G.....
JQ922557.1 : .....G.....A.....AG.....G.....
KC261634.1 : .....T.....G.....C.....G.....G.....C.....
KC762681.1 : .....A.....T.....G.....C.....G.....C.....C.C.....A.....
KC762691.1 : .....A.....C.....G.G.....C.....C.C.....C.....
KF041257.1 : .....G.....C.....A.....G.....G.....
KF954949.1 : .....T.....G.....A.....C.....G.....G.....
KF955332.1 : .....T.....G.....
KF955333.1 : .....T.....C.....C.....
KF955474.1 : .....G.....A.....G.....G.....
KF955476.1 : .....T.....G.....
KF955507.1 : .....T.....C.....C.....
KJ622191.1 : .....AT.....G.....A.....G.....
KJ622195.1 : .....AT.....G.....A.....G.....
KJ737430.1 : .....T.....G.....

KP406805.1 :
KR296744.1 :
KT424097.1 :
KU050695.1 :
KU216209.1 :
KU509279.1 :
KU509280.1 :
KU509281.1 :
KU509283.1 :
KU509284.1 :
KU509285.1 :
KU509286.1 :
KU725666.1 :
KX380841.1 :
KX855927.1 :
KY670634.1 :
KY863456.1 :
KY921907.1 :
LC436676.1 :
LT898451.1 :
LT898452.1 :
MF370226.1 :
MF682968.1 :
MF682975.1 :
MG721061.1 :
MH544651.1 :
MH822957.1 :
MH823209.1 :
MH888332.1 :
MH891766.1 :
MK005258.1 :
MK506265.1 :
MN018383.1 :
MN018388.1 :
MN083245.1 :
MN083246.1 :
MN227699.1 :
MN227702.1 :
MN227703.1 :
MN922036.1 :
MN922041.1 :
MN964274.1 :
NC_001475.2 :

780 * 800 * 820 * 840 * 860 * 880
Asia consensus : GTGTAAGCACACATA GTGGACAGAGGCTGGGGAAA GGTGTGGTTT TTGGCAAGGGAAGCTTGGTAAC TGGCGCAA TTTCATGCTCTGGAACCAATAGAGGGAA
AB189127.1 :
AB214882.1 :
AF317645.1 :
AY099336.1 :
AY496873.2 :
AY496877.2 :
AY496879.2 :
AY648961.1 :
AY676351.1 :
AY766104.1 :
AY770511.2 :
AY776329.1 :
AY858038.2 :
AY858046.2 :
AY923865.1 :
DQ401689.1 :
DQ401694.1 :
DQ401695.1 :
DQ675522.1 :
DQ675531.1 :
DQ863638.1 :
EU081186.1 :
EU081203.1 :
EU081215.1 :
EU367962.1 :
FJ461329.1 :
FJ461338.1 :
FJ644564.1 :
FJ744738.1 :
FJ810414.1 :

QQ199889.1	:		T	A.	G.							T	C	T
GQ466079.1	:		T	A.	G.							T	C	G
GQ868571.1	:		T	A.T	G.	A.						T	C	
GQ868575.1	:		T	A.	G.	A.						T	C	
GU131909.1	:	A.		T.	T.			A.A.		G.		T	A.	
GU131915.1	:	A.		T.	T.			A.A.		G.			A.	
GU131936.1	:			T.	T.			A.A.		G.			G.	A.T
GU131944.1	:	A.		T.	T.			A.A.		G.			A.	
GU131954.1	:		T	A.	G.	A.					T	A.	C.	
GU363549.1	:		T	A.	G.								C.	G.
HG316484.1	:	A.		T.	T.		C	A.A.		G.			A.	
JF295012.1	:	A.		T.	T.			A.A.		G.			A.	
JN368477.1	:	A.		T.	T.			A.A.		G.			G.	A.
JN662391.1	:		T	A.	G.						T		C.	G.
JQ045687.1	:	A.		T.	T.			A.A.		G.			A.	
JQ045688.1	:	A.		T.	T.			A.A.		G.			A.	
JQ045689.1	:	A.		T.	T.			A.A.		G.			A.	
JQ045690.1	:	A.		T.	T.			A.A.		G.			A.	
JQ045691.1	:			T.	T.			A.A.		G.		A.	A.	
JQ045692.1	:			T.	T.			A.A.		G.			A.	
JQ045693.1	:			T.	T.			A.A.		G.			A.	
JQ045694.1	:			T.	T.			A.A.		G.			A.	
JQ045695.1	:			T.	T.			A.A.		G.			A.	
JQ411814.1	:		T	A.	G.		C				G	T.	C.	
JQ922555.1	:		T	A.	G.							T	A.	T
JQ922557.1	:		T	A.	G.							T	C.	G.
KC261634.1	:	A.C		T.	T.		C	A.A.		G.			A.	
KC762681.1	:				G.							G.	T.	TTT
KC762691.1	:				T.									TTTT
KFO41257.1	:		T	A.	G.						T		C.	G.
KF954949.1	:			A.T		G.	C		C			T	C.	G.
KF955332.1	:	A.		T.	T.			A.A.		G.			A.	
KF955333.1	:	A.		T.	T.			A.A.		G.			A.	
KF955474.1	:		T	A.	G.		C				G	T	C.	
KF955476.1	:	A.		T.				A.A.		G.			CTA	T
KF955507.1	:	A.		T.	T.			A.A.		G.			G.	A.
KJ622191.1	:	A.		T.	T.	T.		A.A.	T	G.		A.		A.T
KJ622195.1	:	A.		T.	T.	T.		A.A.	T	G.		A.		A.T
KJ737430.1	:	A.		T.	T.			A.A.		G.				A.T
KP406805.1	:		T											TAT
KR296744.1	:	A.		T.	T.	T.		A.A.	T	G.		A.		A.T
KT424097.1	:	A.		T.	T.			A.A.	T	G.		A.		A.T
KU050695.1	:		T								G.			TAT
KU216209.1	:		TG.	A.	G.		C					T	C.	G.
KU509279.1	:	C	T.											T
KU509280.1	:	A.		T.	T.		C	A.A.		G.		A.		A.
KU509281.1	:		T		G.						T		C.	G.
KU509283.1	:		T	T.	G.			A.			T		C.	
KU509284.1	:	A.		T.	T.		C	A.A.		G.				A.
KU509285.1	:								C			A.		TT
KU509286.1	:		T	A.	G.									

[illegible]

MN227702.1 : .....TAC.....C.....C.....C.A.....
MN227703.1 : .....TAC.....C.....C.....C.A.....
MN922036.1 : .....TAC.....G.....C.....C.....C.A.....
MN922041.1 : .....C.G.....A.....C.....G.....C.A.....
MN964274.1 : .....TAC.....C.....C.....C.A.....
NC_001475.2 : .....AC.....C.....C.....C.A.....

Asia consensus : CAAAGCATGGATGTACATAGACAATGGTTTTTTCACCTTCCTCTACCTGGACATCAGGAGCTACACAGAAACACCAACTTGGAAACAGGAGAGCTCTCTGTGACAT
* 1120 * 1140 * 1160 * 1180 * 1200 *
AB189127.1 : .....G.....C.....T.A.....
AB214882.1 : .....G.....C.....T.A.....
AF317645.1 : .....C.....T.....C.....A.....
AY099336.1 : .....C.....G.....C.....G.....
AY496873.2 : .....G.....C.....C.....C.....
AY496877.2 : .....G.....C.....C.....C.....
AY496879.2 : .....T.....T.....G.....C.....A.....
AY648961.1 : .....C.....T.....C.....T.A.....
AY676351.1 : .....C.....G.....C.....C.....G.....A.....
AY766104.1 : .....G.....C.....C.....GG.....
AY770511.2 : .....G.....C.....G.....C.....
AY776329.1 : .....A.G.....C.....T.A.....
AY858038.2 : .....G.....C.....C.....T.A.....
AY858046.2 : .....C.....G.....C.....T.A.....
AY923865.1 : .....C.....G.....C.....G.....A.....
DQ401689.1 : .....G.....C.....C.....C.....
DQ401694.1 : .....A.....C.....A.....
DQ401695.1 : .....A.....C.....A.....
DQ675522.1 : .....C.....G.....C.....G.....A.....
DQ675531.1 : .....C.....G.....C.....G.....A.....
DQ863638.1 : .....C.....C.....G.....
EU081186.1 : .....C.....G.....C.....G.....
EU081203.1 : .....C.....G.....C.....G.....
EU081215.1 : .....G.....C.....C.....G.....
EU367962.1 : .....C.....G.....C.....T.....G.....A.....
FJ461329.1 : .....C.....G.....C.....G.....A.....
FJ461338.1 : .....C.....G.....C.....G.....A.....
FJ644564.1 : .....C.....C.....G.....C.....
FJ744738.1 : .....C.....G.....C.....G.....A.....
FJ810414.1 : .....C.....G.....C.....G.....A.....
GQ199889.1 : .....C.....T.....C.....G.....C.....
GQ466079.1 : .....C.....C.....G.....C.....
GQ868571.1 : .....G.....C.....G.....
GQ868575.1 : .....G.....C.....G.....
GU131909.1 : .....C.....G.....C.....G.....A.....
GU131915.1 : .....C.....G.....C.....G.....A.....
GU131936.1 : .....C.....G.....C.....G.....C.A.....
GU131944.1 : .....C.....C.....G.....T.....A.....
GU131954.1 : .....G.....C.....C.....A.....T.....
GU363549.1 : .....T.....C.....G.....G.....C.....
HG316484.1 : .....C.....G.....C.....C.....G.....A.....
JF295012.1 : .....C.....G.....C.....G.....A.....
JN368477.1 : .....C.....G.....C.....G.....A.....
JN662391.1 : .....T.....C.....G.....G.....C.....
JQ045687.1 : .....C.....G.....G.....C.....A.....
JQ045688.1 : .....C.....G.....C.....G.....A.....
JQ045689.1 : .....C.....G.....C.....G.....A.....
JQ045690.1 : .....C.....G.....C.....G.....A.....
JQ045691.1 : .....C.....G.....C.....G.....A.....
JQ045692.1 : .....C.....G.....C.....G.....A.....
JQ045693.1 : .....C.....G.....C.....G.....A.....
JQ045694.1 : .....C.....G.....C.....G.....A.....
JQ045695.1 : .....C.....G.....C.....G.....A.....
JQ411814.1 : .....G.....AT.C.C.C.....C.CAC.....A.....T.G.C.....G.....A.C.T.....
JQ922555.1 : .....G.....C.....C.....C.....C.....A.....T.G.C.....G.....A.C.T.....
JQ922557.1 : .....C.....G.....C.....C.....C.....C.....G.....C.....
KC261634.1 : .....C.....G.....C.....CT.....C.....G.....C.....G.....A.....A.....
KC762681.1 : .....G.....G.....G.....C.....C.....A.....
KC762691.1 : .....G.....G.....C.....T.A.....
KF041257.1 : .....C.....C.....G.....C.....C.....
KF954949.1 : .....C.....C.....C.....G.....C.....
KF955332.1 : .....C.....G.....C.....C.....A.....G.....A.....
KF955333.1 : .....C.....G.....C.....C.....G.....A.....
KF955474.1 : .....G.....C.....G.....
KF955476.1 : .....C.....C.....G.....
KF955507.1 : .....C.....G.....C.....A.....
KJ622191.1 : .....T.....C.....C.....T.....G.....

FJ744738.1	:			G.								G.		G.		T.		T.
FJ810414.1	:			G.								G.		G.		T.		T.
GQ199889.1	:	C.		G.								T.		C.	A.			
GQ466079.1	:			G.								T.C.			A.			
GQ868571.1	:	T.	C.	G.								T.C.	T.		C.	A.		
GQ868575.1	:	T.	C.	G.								T.C.	T.		C.	A.		
GU131909.1	:			G.	G.							G.				T.		T.
GU131915.1	:			G.	G.							G.				T.		T.
GU131936.1	:			G.	G.							G.				T.		T.
GU131944.1	:			G.	G.							G.				T.		T.
GU131954.1	:	T.	C.	G.								T.C.	T.		C.	A.		
GU363549.1	:		C.	G.				A.				T.C.			C.	A.		
HG316484.1	:											G.				T.	T.	G.
JF295012.1	:			G.	G.							G.				T.		T.
JN368477.1	:		T.	G.	G.							G.				T.		T.
JN662391.1	:		C.	G.				A.				T.C.			C.	A.		
JQ045687.1	:			G.	G.							G.				T.		T.
JQ045688.1	:			G.	G.							G.				T.		T.
JQ045689.1	:			G.	G.							G.				T.		T.
JQ045690.1	:			G.	G.							G.				T.		T.
JQ045691.1	:			G.	G.							G.				T.	T.	T.
JQ045692.1	:			G.	G.							G.				T.	T.	T.
JQ045693.1	:			G.	G.							G.				T.	T.	T.
JQ045694.1	:			G.	G.							G.				T.		T.
JQ045695.1	:			G.	G.							G.				T.		T.
JQ411814.1	:		C.	G.		C.					T.T.C.			C.	A.			
JQ922555.1	:		C.	A.		G.				T.	T.			C.	A.			
JQ922557.1	:			G.							T.T.C.			C.	A.			
KC261634.1	:			G.							G.G.					T.		T.G.
KC762681.1	:	T.		C.G.		A.T.								C.		C.		
KC762691.1	:		C.	C.		A.										C.		
KF041257.1	:			G.	G.						T.C.			C.	A.			
KF954949.1	:			G.							T.C.			C.	A.			
KF955332.1	:			G.								G.		G.		T.		T.
KF955333.1	:			G.	G.	G.						G.				T.		T.
KF955474.1	:		C.	G.		C.					T.C.			C.	A.			
KF955476.1	:			G.								G.				T.		T.
KF955507.1	:			G.	G.							G.				T.		T.
KJ622191.1	:			G.		C.						G.				T.		T.
KJ622195.1	:			G.		C.						G.				T.		T.
KJ737430.1	:			G.								G.				T.		T.
KP406805.1	:	T.		G.				A.			T.					C.T.		
KR296744.1	:			G.		C.						G.						

[illegible]

[illegible]

[illegible]

[illegible]

[illegible]

[illegible]

Europe consensus : ATGTCGGCTGAAGGAGCTTGGAGACAGGTCGAGAAGGTAGAGACATGGGCCTTTAGGCACCCAGGGTTCACAATACTAGCCCTATTTCCTTGCCCATACATAGGCACTTG
AY744679.1 : .....
AY744684.1 : .....

*   460   *   480   *   500   *   520   *   540   *

Europe consensus : CTTGACCCAGAAAGTGGTTATTTTCATACTACTAATGCTGGTCACCCATCCATGACAATGAGATGTGTGGGAGTAGGAAACAGAGATTTTGTGGAAGCCCTATCAGGAG
AY744679.1 : .....
AY744684.1 : .....

560   *   580   *   600   *   620   *   640   *   660

Europe consensus : CTACGTGGGTTGACGTGGTGTCTCGAGCACGGTGGGTGTGTGACTACTATGGCTAAGAACAAGCCACGTTGGATATAGAGCTCCAGAAGACCGAGGCCACCAACTGGCG
AY744679.1 : .....
AY744684.1 : .....

*   680   *   700   *   720   *   740   *   760   *

Europe consensus : ACCCTAAGGAACTATGTATTGAGGAAAAATTACCAAGTAACAACCGACTCAAGGTGCCCCACCCAAGGGGAAGCGATTTTACCTGAGGAGCAGGACCAGAACTACGT
AY744679.1 : .....
AY744684.1 : .....

780   *   800   *   820   *   840   *   860   *   880

Europe consensus : GTGTAAGCACACATACGTGGACAGAGGCTGGGGAACGGTTGTGGTTTGTGTTGGCAAGGGAAGCCTGGTAACATGCGCGAAATTCAATGTTTGAATCAATAGAGGGAA
AY744679.1 : .....
AY744684.1 : .....

*   900   *   920   *   940   *   960   *   980   *

Europe consensus : AAGTGGTGACATGAGAACCTCAAATACACCGTCATCATCACATGCACACAGGAGATCAACACCAGGTGGGAAATGAAACGCAGGGAGTCACGCTGAGATAACACCC
AY744679.1 : .....
AY744684.1 : .....

1000   *   1020   *   1040   *   1060   *   1080   *   1100

Europe consensus : CAGGCATCAACCGTTGAAGCCATCTTGCCTGAATATGGAACCCCTGGGCTAGAATGTTACCACGGACAGGCTAGATTTCAATGAAATGATTTTGTGACAATGAAGAA
AY744679.1 : .....
AY744684.1 : .....

*   1120   *   1140   *   1160   *   1180   *   1200   *

Europe consensus : CAAAGCATGGATGGTACATAGACAATGGTTTTTTGACCTGCCTTTACCATGGACATCAGGAGCTACAACAGAAACACCAACCTGGAATAAGAAAGAGCTTCTTGTGACAT
AY744679.1 : .....
AY744684.1 : .....

1220   *   1240   *   1260   *   1280   *   1300   *   1320

Europe consensus : TCAAAAACGCACATGCAAAAAAGCAAGAAGTAGTAGTCCTTGGATCGCAAGAGGGAGCAATGCACACAGCACTGACAGGAGCTACAGAGATCCAAACCTCAGGAGGCACA
AY744679.1 : .....
AY744684.1 : .....

*   1340   *   1360   *   1380   *   1400   *   1420   *

Europe consensus : AGTATTTTGCGGGGCACTTAAAAATGTAGACTCAAGATGGACAAATTGGAAGTCAAGGGGATGAGCTATGCAATGTGCTTGAATGCCTTCGTGTTGAAGAAAGAGTCTC
AY744679.1 : .....
AY744684.1 : .....

1440   *   1460   *   1480   *   1500   *   1520   *   1540

Europe consensus : TGAACGCAACATGGGACGATACTCATTAAAGGTTGAGTACAAAGGGGAAGATGCACCTTGCAAGATTCTTCTCCAGGAGGTGGACAAGGGAAAGCCCAATGGCA
AY744679.1 : .....
AY744684.1 : .....

*   1560   *   1580   *   1600   *   1620   *   1640   *

Europe consensus : GACTGATCACAGCTAACCCAATAGTGACCAAGAAGGAGGAGCCTGTCAACATCGAGGCAGAACCTCCTTTTGGGAAAAGTAATATAGTAATTGGAATTGGAGACAAAGCC
AY744679.1 : .....
AY744684.1 : .....

1660   *   1680   *   1700   *   1720   *   1740   *   1760

Europe consensus : TTGAAAATCAACTGGTACAAGAAGGGAAGCTCGATTGGGAAGATGTTGAGGCCTACTGCCAGAGGTGCAAGGCCGATGGCCATCTTGGGAGACACAGCCTGGGACTTTGG
AY744679.1 : .....
AY744684.1 : .....

	120	*	140	*	160	*	180	*	200	*	220
North America consensus :	AGCCATGGACTT	GGGAGAGATGTGTGAT	ACACGGT	ACTTACAAATG	CC	CACAT	CCGAAGTGAACCT	GATGAC	ATTGACTGCTGGTG	TAACT	ACATCAACAT
EU854298.1	:	.	.	.	T.	T.	.	.	.	C.	.
FJ182007.1	:	A.	.	.	.	TG.	.	.	.	C.	T.
FJ390375.1	:	.	.	.	.	T.	.	.	.	C.	.
FJ547084.1	:	.	.	.	.	T.	.	.	.	C.	.
FJ562107.1	:	.	.	.	.	T.	.	.	.	C.	.
FJ898441.1	:	A.	.	.	.	T.	.	C.	T.	C.	T. G.
FJ898459.1	:	.	.	.	.	T.	.	.	.	C.	T.
HM181975.1	:	.	.	.	.	.	.	.	.	C.	.
HM631864.1	:	.	.	.	.	.	.	.	.	.	.
HM756282.1	:	.	.	.	.	.	.	.	.	.	.
HQ166034.1	:	.	.	.	.	.	.	.	.	.	.
HQ541789.1	:	.	.	.	.	.	.	.	.	.	.
HQ671177.1	:	.	.	.	.	.	.	.	.	.	.
HQ705619.1	:	.	.	.	.	.	.	.	.	.	.
HQ891025.1	:	.	.	.	.	.	.	.	.	.	.
JF920407.1	:	.	.	.	.	.	.	.	.	.	.
JF937627.1	:	.	.	.	.	.	.	.	.	.	.
JF937643.1	:	.	.	.	.	.	.	.	.	.	.
JF937652.1	:	.	A.	.	T.	.	.	.	.	.	.
JN000938.1	:	A.	.	.	.	.	.	.	.	.	.
JN093514.1	:	.	.	.	.	.	.	.	.	.	.
JN183884.1	:	.	.	.	.	.	.	.	.	.	.
KF921916.1	:	.	.	.	.	.	.	.	.	.	.
KF955468.1	:	.	.	.	.	T.	.	.	.	C.	.
KF973476.1	:	.	.	.	.	.	.	.	.	.	.
KF973478.1	:	.	.	.	.	.	.	G.	.	.	.
KF973483.1	:	.	.	.	.	.	.	.	.	.	.
KT726346.1	:	.	.	.	.	T.	.	.	.	C.	T.
KT726358.1	:	.	.	.	.	T.	.	.	.	C.	T.
KU509278.1	:	.	.	T.	.	T.	.	.	.	C.	T.

	*	240	*	260	*	280	*	300	*	320	*
North America consensus :	GGGTGACTTATGGAAC	TGCAATCAAGC	GGAGAGCA	TAGACGCGA	AAGAGATCAGTGGCGT	TAGCTCCCCATGTCGGCATGGGACT	TGACAC	CGCAC	CAAACCTGG		
EU854298.1	:	.	.	.	.	.	.	.	G.	.	.
FJ182007.1	:	.	.	C.	.	.	.	.	G.	.	T.
FJ390375.1	:	.	.	.	.	.	.	.	G.	.	.
FJ547084.1	:	.	.	.	.	.	.	.	.	.	.
FJ562107.1	:	.	.	.	.	.	.	.	G.	.	.
FJ898441.1	:	.	.	T.	.	.	.	.	G.	.	.
FJ898459.1	:	.	.	.	.	.	.	.	.	.	.
HM181975.1	:	.	.	.	.	.	.	.	.	.	.
HM631864.1	:	.	.	.	.	.	.	.	.	.	.
HM756282.1	:	.	.	.	.	.	.	.	.	.	.
HQ166034.1	:	.	.	.	.	.	.	.	.	.	.
HQ541789.1	:	.	.	.	.	.	.	.	.	.	.
HQ671177.1	:	.	.	.	.	.	.	.	.	.	.
HQ705619.1	:	.	T.	.	.	.	.	.	.	.	.
HQ891025.1	:	.	T.	.	.	.	.	.	.	.	.
JF920407.1	:	.	.	.	.	.	.	.	.	.	.
JF937627.1	:	.	.	.	.	.	.	.	.	.	.
JF937643.1	:	.	.	.	.	.	.	.	.	.	.
JF937652.1	:	.	.	.	.	.	.	.	.	.	.
JN000938.1	:	.	.	.	.	.	.	.	G.	.	.
JN093514.1	:	.	.	.	.	.	.	.	.	.	.
JN183884.1	:	.	.	.	.	.	.	.	.	.	.
KF921916.1	:	.	T.	.	.	.	.	.	.	.	.
KF955468.1	:	.	.	.	.	.	.	.	G.	.	.
KF973476.1	:	.	.	.	.	.	.	.	.	.	.
KF973478.1	:	.	.	.	.	.	.	.	.	.	.
KF973483.1	:	.	.	.	T.	.	.	.	.	.	.
KT726346.1	:	T.	.	.	.	.	.	.	.	.	.
KT726358.1	:	.	.	.	.	.	.	.	.	.	.
KU509278.1	:	.	.	.	.	.	.	.	.	.	.

	340	*	360	*	380	*	400	*	420	*	440
North America consensus :	ATGTC	GCTGAAGGAGCTTGGAGGCAAGT	GAGAA	GTAGA	TACATGGGCC	CTTAGGCACCCAGGGTT	TACCATACTAGC	CTATTCTTGCCCA	TTA	TATAGGCACATC	
EU854298.1	:	.	.	.	.	.	.	.	.	.	T.
FJ182007.1	:	A.	.	.	.	.	.	.	.	.	T.
FJ390375.1	:	.	.	.	.	.	.	.	.	.	T.
FJ547084.1	:	.	.	.	.	.	.	.	.	.	T.
FJ562107.1	:	.	.	A.	.	.	.	.	.	.	T.

[illegible]

	*	1120	*	1140	*	1160	*	1180	*	1200	*
North America consensus :	CAAAGCATGGATGGTACA	TAGACAATGGTTTTTTTGACCTACCT		TACCATGGACATCAGGAGC		ACAACGGAAACAC	AAC	TGGAACAG		AAAGGAGCTTCTTGTGACAT	
EU854298.1	:									G.	
FJ182007.1	:					C.				G.	
FJ390375.1	:									G.	
FJ547084.1	:									G.	
FJ562107.1	:									G.	
FJ898441.1	:	C.								G.	
FJ898459.1	:									G.	
HM181975.1	:								T.		
HM631864.1	:										
HM756282.1	:										
HQ166034.1	:								T.		
HQ541789.1	:										
HQ671177.1	:										
HQ705619.1	:				T.			T.			
HQ891025.1	:				T.						
JF920407.1	:								T.		
JF937627.1	:								T.		
JF937643.1	:										
JF937652.1	:										
JN000938.1	:										
JN093514.1	:										
JN183884.1	:				T.						
KF921916.1	:				T.						
KF955468.1	:									G.	
KF973476.1	:				T.		C.				
KF973478.1	:										
KF973483.1	:								T.		
KT726346.1	:									G.	
KT726358.1	:									G.	
KU509278.1	:									G.	

		1220	*	1240	*	1260	*	1280	*	1300	*	1320
North America consensus :	T	CAAAAA	GCACATGCGAAG	AAAACAAGTAGTTGTCCTTGGATCGCAAGAGGGAGCAATGCATACCGCAT		TGACAGGAGCCACAGAAATCCAAAAC		TCAGGAGGCACA				
EU854298.1	:	T.		A.								
FJ182007.1	:	T.		A.								
FJ390375.1	:	T.		A.								
FJ547084.1	:	T.		A.								
FJ562107.1	:	T.		A.								
FJ898441.1	:	T.		A.								
FJ898459.1	:	T.										
HM181975.1	:											
HM631864.1	:											
HM756282.1	:							C.				
HQ166034.1	:											
HQ541789.1	:											
HQ671177.1	:											
HQ705619.1	:							C.				
HQ891025.1	:							C.				
JF920407.1	:											
JF937627.1	:											
JF937643.1	:											
JF937652.1	:											
JN000938.1	:											
JN093514.1	:											
JN183884.1	:											
KF921916.1	:							C.				
KF955468.1	:	T.		A.								
KF973476.1	:											
KF973478.1	:											
KF973483.1	:							T.				
KT726346.1	:											
KT726358.1	:											
KU509278.1	:	T.	T.									

	*	1340	*	1360	*	1380	*	1400	*	1420	*
North America consensus :	A	TATTTTTC	GGGCACCTTAAAAATGC	CAGACTTAA	ATGGACAA	TTGGAAC	CTAAGGGGATGAGT	TATGCAATGTGCACGAATACCTTTGTGTTGAAGAAAGAAGTCTC			
EU854298.1	:	C.		T.				C.			
FJ182007.1	:	C.		T.				C.			
FJ390375.1	:	C.		T.				C.			
FJ547084.1	:	C.		T.				C.			
FJ562107.1	:	C.		T.				C.			
FJ898441.1	:	CC.		T.	A.			C.			
FJ898459.1	:			T.				C.			

[illegible]

FJ898455.1 : ..... C ..... C ..... A .....
 FJ898456.1 : ..... C ..... C ..... A .....
 JN406515.1 : ..... C ..... G .....
 KY794788.1 : ..... C ..... T .....

340 * 360 * 380 * 400 * 420 * 440
 Oceania consensus : ATGTCGGCTGAAGGACGTTGGAGACAGGTCGAGAAGGTAGATACATGGGCCTTTAGCACCCAGGGTTCACAATACTAGCCCTATTTCTTGCCCAATACATAGGCACTTC
 FJ898455.1 : .....
 FJ898456.1 : ..... C .....
 JN406515.1 : ..... A ..... A ..... T ..... A ..... C .....
 KY794788.1 : ..... A ..... C .....

460 * 480 * 500 * 520 * 540 *
 Oceania consensus : TTGACCCAGAAAGTGGTTATTTTCATACTACTAATGCTGGTCACCCCATCCATGACAATAGATGTGTGGGAGTAGGAAACAGAGATTGTGGAAGGCTATCAGGAG
 FJ898455.1 : .....
 FJ898456.1 : .....
 JN406515.1 : ..... C ..... C ..... T .....
 KY794788.1 : T ..... C ..... T .....

560 * 580 * 600 * 620 * 640 * 660
 Oceania consensus : CTACCTGGGTTGACGTGGTGTCTCGAGCAAGTGGGTGTGTGACTACTATGGCTTAAGAACAAGCCACGTTGGATATAGAGCTCCAGAAGACCGAGGCCACCCAACTGGCG
 FJ898455.1 : .....
 FJ898456.1 : ..... C .....
 JN406515.1 : ..... A ..... A ..... T ..... C ..... C ..... A .....
 KY794788.1 : ..... A ..... C ..... C ..... A .....

680 * 700 * 720 * 740 * 760 *
 Oceania consensus : ACCCTAAGGAACTATGTATTGAGGGAAAAATTACCAATGTGACAACCGACTCAAGGTGCCCCACCCAAGGGGAAGCGATTTCCTGAGGAGCAAGACCAGAACTACGTT
 FJ898455.1 : ..... T ..... A .....
 FJ898456.1 : ..... A .....
 JN406515.1 : ..... C ..... C ..... A .....
 KY794788.1 : ..... C ..... C ..... T ..... C .....

780 * 800 * 820 * 840 * 860 * 880
 Oceania consensus : GTGTAAGCACACATACGTGGACAGAGGCTGGGGAACGGTTGTGGTTTGTTTGGCAAGGGAAGTTGGTAACATGCGCGAAATTTCAATGTTGGAATTAATGAGGGAA
 FJ898455.1 : ..... C .....
 FJ898456.1 : ..... C ..... G ..... T .....
 JN406515.1 : ..... T ..... C ..... T .....
 KY794788.1 : ..... C .....

900 * 920 * 940 * 960 * 980 *
 Oceania consensus : AAGTGGTCAAGCATGAGAACCTCAAATACACCGTCATCATACAGTGCAACAGGAGATCAACACCAGGTGGGAAATGAAACGCAGGGGTGTCACGGCTGAGATAACACCC
 FJ898455.1 : ..... C .....
 FJ898456.1 : ..... C .....
 JN406515.1 : ..... T ..... T ..... C ..... A .....
 KY794788.1 : ..... T ..... C ..... T .....

1000 * 1020 * 1040 * 1060 * 1080 * 1100
 Oceania consensus : CAGGCATCAACCGTCGAAGCCATCTTGCTGAATATGGAACCCCTGGGCTAGAATGTCACCACGGACAGGTTTGGATTCAATGAAATGATTTGTTGACAATGAAGAA
 FJ898455.1 : ..... A ..... C ..... A .....
 FJ898456.1 : ..... T ..... C ..... A .....
 JN406515.1 : ..... A ..... C ..... C .....
 KY794788.1 : ..... A ..... C .....

1120 * 1140 * 1160 * 1180 * 1200 *
 Oceania consensus : CAAAGCATGGATGGTCAAGACAATGGTTTTTTGACCTGCCTTTACCATGGACATCAGGGGCTACAACAGAAACACCAACCTGGAATAAGAAAGAGCTTCTGTGACAT
 FJ898455.1 : ..... A .....
 FJ898456.1 : ..... A .....
 JN406515.1 : ..... G ..... A ..... C .....
 KY794788.1 : ..... C ..... A ..... C ..... C .....

1220 * 1240 * 1260 * 1280 * 1300 * 1320
 Oceania consensus : TCAAAAACGCACATGCAAAAAACAAGAAGTAGTAGTCCTTGGATCGCAAGAGGGAGCAATGCACACAGCACTGACAGGAGCTACAGAGATCCAAACCTCAGGAGGCACA
 FJ898455.1 : ..... G .....
 FJ898456.1 : ..... C .....
 JN406515.1 : ..... C ..... G .....
 KY794788.1 : ..... C .....

JN406515.1 : ..... A ..... A ..... C ..... A .....
 KY794788.1 : ..... A ..... G ..... C ..... A .....

Oceania consensus : AGCATGGAATGTGTGGGAGGTGGAAGATTACGGGTTCGGAGTTTCACAACATAATATATGGCTGAACTCCGAGAGA
 FJ898455.1 : ..... C .....
 FJ898456.1 : ..... C .....
 JN406515.1 : ..... C ..... G .....
 KY794788.1 : ..... C ..... G .....

6. DENV-3 sequences from South America.

South America consensus : TTCCACCTTACTTCACGATGGGAGAGCCGCGCATGATGTGGGAAATGAAAGAGGAAATCCCTACTTTTAAACAGCCTCTGGAATCAACATGTGCACACTCAT
 AY679147.1 : .....
 EF629369.1 : .....
 EF643017.1 : .....
 FJ177308.1 : .....
 FJ639776.1 : ..... C ..... T .....
 FJ639804.1 : ..... T .....
 FJ639827.1 : ..... T .....
 GU131868.1 : .....
 GU131873.1 : ..... A .....
 GU131877.1 : ..... C .....
 HQ235027.1 : ..... A ..... T .....
 HQ332171.1 : ..... A .....
 JF808120.1 : ..... G .....
 JF808128.1 : .....
 JN697379.1 : ..... A .....
 JX669490.1 : .....
 JX669501.1 : .....
 KC425219.1 : .....
 KF955473.1 : ..... C ..... T .....
 KF955477.1 : ..... C ..... G ..... T .....
 KF955486.1 : .....
 KF955487.1 : ..... T .....
 KJ189270.1 : ..... T .....
 KJ189292.1 : ..... A .....
 KJ189297.1 : ..... T .....
 KJ189301.1 : ..... CT .....
 KJ643590.1 : ..... T .....

South America consensus : AGCCATGGATTGGGAGAGATGTGTGATGACACGGTCACTTACAAATGCCCCCATACCGAAGGGAACCTGAAGACATTGATGCTGGTGTAACCTTACATCAACAT
 AY679147.1 : .....
 EF629369.1 : ..... C .....
 EF643017.1 : .....
 FJ177308.1 : .....
 FJ639776.1 : ..... A .....
 FJ639804.1 : ..... T ..... C .....
 FJ639827.1 : ..... T ..... C .....
 GU131868.1 : ..... T .....
 GU131873.1 : .....
 GU131877.1 : .....
 HQ235027.1 : .....
 HQ332171.1 : ..... T ..... T ..... C .....
 JF808120.1 : .....
 JF808128.1 : ..... G ..... C .....
 JN697379.1 : ..... T ..... G ..... G .....
 JX669490.1 : .....
 JX669501.1 : .....
 KC425219.1 : .....
 KF955473.1 : .....
 KF955477.1 : ..... G ..... G .....
 KF955486.1 : ..... T ..... C .....
 KF955487.1 : ..... T ..... C .....
 KJ189270.1 : ..... C ..... C .....
 KJ189292.1 : ..... T ..... C .....
 KJ189297.1 : ..... C ..... C .....
 KJ189301.1 : ..... C .....
 KJ643590.1 : ..... C .....

		*	240	*	260	*	280	*	300	*	320	*
South America consensus :	GGGTGACTTATGGAAC	TGCAATCAAGC	GG	GAGCA	TAGACGCGA	AAGAGATC	GTGGCGTT	GCTCCCATGTCGGCATGGGACTAGA	TACACG	TAC	CAAAC	TGG
AY679147.1	:	.....C.....		.....G.....		.....		.....G.....		.....		
EF629369.1	:	.....C.....		.....G.....		.....		.....		.....		
EF643017.1	:	.....C.....		.....G.....		.....		.....		.....		
FJ177308.1	:	.....C.....		.....G.....		.....		.....		.....		
FJ639776.1	:	.....		.....		.....		.....		.....		
FJ639804.1	:	.....		.....		.....		.....		G.....		
FJ639827.1	:	.....		.....		T.....		.....		G.....T.....		T.....
GU131868.1	:	.....C.....		.....G.....		T.....		.....		.....		
GU131873.1	:	.....C.....		.....G.....		.....G.....		.....		.....		
GU131877.1	:	.....C.....		.....G.....		.....		.....		.....		
HQ235027.1	:	.....C.....		.....		.....		.....		.....		
HQ332171.1	:	.....		.....		.....		.....		G.....		
JF808120.1	:	.....C.....		.....G.....		.....		G.....		.....		
JF808128.1	:	.....C.....		.....		T.....		.....		.....		
JN697379.1	:	.....A.....		T.....		T.....		.....		G.....		
JX669490.1	:	.....C.....		.....G.....		.....		.....		.....		
JX669501.1	:	.....C.....		.....G.....		.....		.....		.....		
KC425219.1	:	.....C.....		.....G.....		.....		.....		.....		
KF955473.1	:	.....		.....		.....		.....		.....		
KF955477.1	:	.....		T.....G.....		C.....		.....		G.....		
KF955486.1	:	.....		.....		.....		.....		G.....		
KF955487.1	:	.....		.....		.....		.....		G.....		
KJ189270.1	:	.....		.....		.....		.....		.....		
KJ189292.1	:	.....		.....		.....		.....		.....		
KJ189297.1	:	.....		.....		.....		.....		.....		
KJ189301.1	:	.....		.....		.....		.....		.....		
KJ643590.1	:	.....		.....		.....		.....		.....		

		340	*	360	*	380	*	400	*	420	*	440
South America consensus :	ATGTCGGCTGAAGGAGC	TGGAG	CAAGTCGAGAAGGTAGAGAC	TGGGCCCTTAGGCA	CCAGGGTT	ACCATACTAGGC	CTATTCTTGC	CATTACATAGGCACATC				
AY679147.1	:	.....		.....		.....		.....		.....		
EF629369.1	:	.....		.....		.....		.....		.....		
EF643017.1	:	.....		.....		.....		.....		.....		
FJ177308.1	:	.....		.....		.....		.....		.....		
FJ639776.1	:	.....		.....		G.....		.....		.....		T.....
FJ639804.1	:	.....		.....		.....		.....		.....		T.....
FJ639827.1	:	.....		.....		.....		.....		.....		T.....
GU131868.1	:	.....		.....		.....		.....		.....		.....
GU131873.1	:	.....		.....		.....		.....		T.....		.....
GU131877.1	:	.....		.....		.....		.....		.....		.....
HQ235027.1	:	.....		.....		T.....		.....		.....		.....
HQ332171.1	:	.....		.....		.....		.....		.....		T.....
JF808120.1	:	.....C.....		.....		.....		.....		.....		.....
JF808128.1	:	.....		A.....		.....		.....		.....		.....
JN697379.1	:	.....		A.....		.....		T.....		.....		T.....
JX669490.1	:	.....		.....		.....		.....		.....		.....
JX669501.1	:	.....		.....		.....		.....		.....		.....
KC425219.1	:	.....		.....		.....		.....		.....		.....
KF955473.1	:	.....		.....		.....		.....		.....		.....
KF955477.1	:	.....		A.....		.....		T.....		.....		T.....
KF955486.1	:	.....		.....		.....		.....		.....		T.....
KF955487.1	:	.....		.....		.....		.....		G.....		T.....
KJ189270.1	:	.....		.....		.....		.....		.....		.....
KJ189292.1	:	.....		.....		.....		.....		.....		.....
KJ189297.1	:	.....		.....		.....		.....		.....		.....
KJ189301.1	:	.....		.....		.....		.....		.....		.....
KJ643590.1	:	.....		.....		.....		.....		.....		.....

		*	460	*	480	*	500	*	520	*	540	*
South America consensus :	TTGACCCAGAA	GTGGTTATTTTAT	ACTACTAAT	CTGGTCA	CCATCCATG	CAATGAGATG	GTGGGAGT	AGGAAACAG	GATTTTGTGGAAGG	CTATCAGGAG		
AY679147.1	:	.....		.....		.....		.....		.....		
EF629369.1	:	.....		.....		.....		.....		.....		
EF643017.1	:	.....		.....		.....		.....		.....		
FJ177308.1	:	.....		.....		.....		.....		.....		
FJ639776.1	:	.....		.....		.....		.....		.....		
FJ639804.1	:	.....		.....		.....		.....		.....		
FJ639827.1	:	.....		G.....		.....		.....		.....		A.....
GU131868.1	:	.....		.....		.....		.....		.....		.....
GU131873.1	:	.....		.....		.....		.....		.....		.....
GU131877.1	:	.....		.....		.....		.....		.....		.....
HQ235027.1	:	T.....		.....		.....		.....		G.....		.....
HQ332171.1	:	.....		.....		.....		.....		.....		.....
JF808120.1	:	.....		.....		.....		G.....		.....		.....

[illegible][illegible]

	* 1120 *	* 1140 *	* 1160 *	* 1180 *	* 1200 *
South America consensus :	CAAAGCATGGATGGTACATAGACAATGGTTT	TTGACCTACCTG	TACCATGGACATCAGGAGCTACAAC	GGAACACCAAC	TGGAACAGGAAGAACTTCTTGTCAT
AY679147.1	:	:	:	:	:
EF629369.1	:	:	:	:	:
EF643017.1	:	:	:	:	:
FJ177308.1	:	:	:	:	:
FJ639776.1	:	:	:	:	:
FJ639804.1	:	T.	:	:	:
FJ639827.1	:	:	:	:	:
GU131868.1	:	:	:	:	:
GU131873.1	:	:	:	:	:
GU131877.1	:	:	:	:	:
HQ235027.1	:	:	:	:	:
HQ332171.1	:	:	:	:	A.
JF808120.1	:	:	:	:	:
JF808128.1	C.	:	:	:	:
JN697379.1	C.	T.	C.	A.	T.
JX669490.1	:	:	:	:	A.
JX669501.1	:	:	:	:	:
KC425219.1	:	:	:	:	:
KF955473.1	:	:	:	:	:
KF955477.1	C.	:	C.	A.	G.
KF955486.1	:	:	:	T.	A.
KF955487.1	:	:	:	:	:
KJ189270.1	:	:	T.	:	:
KJ189292.1	:	:	:	:	:
KJ189297.1	:	:	:	:	G.
KJ189301.1	:	:	:	:	:
KJ643590.1	:	:	:	:	:

[illegible]

KJ643590.1 : .....T.....

[illegible]

	*<br>2000	*<br>2020	*<br>2040	*<br>2060	*<br>2080	*
South America consensus :	A TGGGATGTCTTATAAACTGGAAAGC	CAAGAACTCAA	TGTGGAAGTGGAATCTTCGTCACAAA	GAGGTCCA	ACCTGGACAGAGCAATA	AAATTCAAGCAGACTC
AY679147.1	:	:	:	:	:	:
EF629369.1	:	:	:	:	:	:
EF643017.1	:	:	:	:	:	:
FJ177308.1	:	:	:	:	:	:
FJ639776.1	:	G.	:	:	:	:
FJ639804.1	:	G.	:	:	:	:
FJ639827.1	: C.	G.	G.	:	:	:
GU131868.1	:	:	:	:	:	:
GU131873.1	:	:	:	:	:	:
GU131877.1	:	:	:	:	:	:
HQ235027.1	:	:	:	:	:	:
HQ332171.1	: C.	G.	:	:	:	:
JF808120.1	:	:	:	:	:	:
JF808128.1	:	:	:	:	:	:
JN697379.1	: G.	C.	T.	T.	C.	T.
JX669490.1	:	:	:	:	:	:
JX669501.1	:	G.	:	:	:	:
KC425219.1	:	:	TCC.	:	:	:
KF955473.1	:	:	:	:	:	:
KF955477.1	: G.	C.	A.	T.	T.	T.
KF955486.1	: G.	:	:	C.	:	:
KF955487.1	: G.	:	:	:	:	:
KJ189270.1	: G.	:	:	:	:	:
KJ189292.1	: G.	A.	:	:	:	:
KJ189297.1	: G.	:	:	:	:	:
KJ189301.1	: G.	:	:	:	:	T.
KJ643590.1	: G.	:	:	:	:	:

[illegible]

		*		2220	*		2240	*		2260	*		2280	*		2300	*
South America consensus :	TGAACTAATAATCTGTGGGAAAACAAATAT	A	A	A	A	A	A	A	A	A	A	A	A	A	A	A	A
AY679147.1	:	.	.	.	.	.	.	.	.	.	.	.	.	.	.	.	.
EF629369.1	:	C.	.	.	.	.	.	.	.	.	.	.	.	.	.	.	.
EF643017.1	:	.	.	.	.	.	.	.	.	.	.	.	.	.	.	.	.
FJ177308.1	:	.	.	A.	.	C.	T.	.	A.	C.	C.	.	G.	.	.	.	.
FJ639776.1	:	.	C.	.	.	.	.	.	.	.	.	.	.	.	.	.	.
FJ639804.1	:	.	.	.	.	.	.	.	.	.	.	.	.	.	.	.	.
FJ639827.1	:	.	.	.	.	.	T.	.	.	.	.	.	G.	.	.	.	.
GU131868.1	:	.	.	.	.	.	A.	.	.	.	.	.	.	.	.	.	.
GU131873.1	:	.	C.	.	.	.	.	.	.	.	.	.	.	.	.	.	.
GU131877.1	:	.	.	.	.	.	.	.	.	.	T.	.	.	.	.	.	.
HQ235027.1	:	.	.	.	.	.	.	.	C.	.	.	.	.	.	.	.	.
HQ332171.1	:	.	.	.	.	.	.	.	.	.	.	.	.	.	.	.	.
JF808120.1	:	.	.	.	.	.	.	.	.	.	.	A.	.	.	.	.	G.
JF808128.1	:	.	T.	.	.	.	.	.	.	.	.	.	.	.	.	.	.
JN697379.1	:	.	.	A.	.	C.	T.	.	A.	C.	C.	.	G.	.	.	.	.
JX669490.1	:	.	.	.	.	.	.	.	.	.	.	.	.	.	.	.	.
JX669501.1	:	T.	.	.	.	.	.	.	.	.	.	.	.	.	.	.	.
KC425219.1	:	.	.	.	.	.	.	.	.	.	.	.	.	.	.	.	.
KF955473.1	:	.	.	.	.	.	.	.	.	.	.	.	.	.	.	.	.
KF955477.1	:	.	A.	.	.	C.	.	.	G.	.	C.	.	G.	.	.	.	.
KF955486.1	:	.	.	.	.	.	.	.	.	.	.	.	.	.	.	.	.
KF955487.1	:	.	.	.	.	.	.	.	.	.	.	.	.	.	.	.	.
KJ189270.1	:	.	.	.	.	.	.	.	.	.	.	.	G.	.	.	.	.
KJ189292.1	:	.	.	.	.	.	.	.	.	.	.	.	G.	.	.	.	.
KJ189297.1	:	.	.	.	.	.	.	.	.	.	.	.	G.	.	.	.	.
KJ189301.1	:	.	.	.	.	G.	.	.	.	.	.	GG.	.	.	.	.	.
KI643590.1	:	.	.	.	.	.	.	.	.	.	.	G.	.	.	.	.	.

[illegible]

		*	460	*	480	*	500	*	520	*	540	*
Consensus	:	CTTGACCCAGAAGGTGGTTATTTTATACTACTAATGCTGGTCACCCCATCCATGACAATGAGATGTGTGGGAGT										
Africa consensus	:								G.	G.		G.
Asia consensus	:		A.									G.
Europe consensus	:		A.	C.								C.
North America consensus	:											
Oceania consensus	:		A.	C.								C.
South America consensus	:											

		560	*	580	*	600	*	620	*	640	*	660
Consensus	:	CTACTGGGTTGACGTGGTGCTGAGCACGGTGGGTGTGTGACTACCATGGCTAAGAACAAGCCCACGTTGGATATAGAGCTTCAGAAGACCGAGGCCACCCAACTGGCG										
Africa consensus	:	A.		A.	G.	C.		C.				
Asia consensus	:					C.		C.	C.			
Europe consensus	:					T.				C.		
North America consensus	:			G.				A.				T.
Oceania consensus	:					T.				C.		T.
South America consensus	:			G.				A.				T.

		*	680	*	700	*	720	*	740	*	760	*
Consensus	:	ACCCTAAGGAACTATGCATTGAGGGGAAAAATTACAACATACAACTGACTCAAGGTGCTCTACCCAAGGGGAAGCGATTTCGCTGAGGAGCAGGACCAAACTACGT										
Africa consensus	:						A.	C.	C.			
Asia consensus	:			A.							A.	
Europe consensus	:		T.	A.	NG.	C.		C.	C.		A.	
North America consensus	:				T.		A.			C.		A.
Oceania consensus	:	A.	T.	A.	G.	G.	C.		C.	C.	A.	
South America consensus	:						A.			G.		

		780	*	800	*	820	*	840	*	860	*	880
Consensus	:	GTGTAAGCATACATACGTGGACAGAGGCTGGGGGAACGGTTGTGTGTTGTTTGGCAAGGGAAGCTTGGTAACATGTGCGAAATTTCAATGTCTTGAACCATATAGAGGGAA										
Africa consensus	:		A.								C.	G.
Asia consensus	:	C.		A.							C.	
Europe consensus	:	C.			A.			C.			T.	T.
North America consensus	:		A.		A.						C.	A.
Oceania consensus	:	C.		A.							T.	T.
South America consensus	:		A.		A.						C.	

		*	900	*	920	*	940	*	960	*	980	*
Consensus	:	AAGTGGTGCAATATGAGAACCTCAATACCGTTCATCATCAGTGCACACAGGAGATCAACACCAGGTGGGAAATGAAACGCAGGGAGTACGGCTGAGATAAACCCT										
Africa consensus	:			G.	T.			C.				G.
Asia consensus	:							C.				C
Europe consensus	:	N.	GC.								N.	C
North America consensus	:					T.						
Oceania consensus	:		GC.									C
South America consensus	:											

		1000	*	1020	*	1040	*	1060	*	1080	*	1100
Consensus	:	CAGGCATCAACGTGAAGCCATCTTGCTGAATATGGAACCTTGGGCTAGATGCTCACCACGGACAGGTTTGAATTCAATGAAATGATTTTGTGACAATGAAGAA										
Africa consensus	:		TAC				G.			C.		AC.A.
Asia consensus	:											A.
Europe consensus	:						T.		C.A.			
North America consensus	:		AC								C.	C.A.
Oceania consensus	:		C.				T.					
South America consensus	:		AC								C.	C.A.

		*	1120	*	1140	*	1160	*	1180	*	1200	*
Consensus	:	CAAAGCATGGATGGTACATAGACAATGGTTTTTGTGACCTACCTCTACCATGGACATCAGGTGCTACAACAGAAACCAACCTGGAACAGTAAGGAGCTTCTGTGACAT										
Africa consensus	:		C.						T.			C.
Asia consensus	:								T.		A.	
Europe consensus	:				G.	T.				T.A.	A.	
North America consensus	:							G.		A.		
Oceania consensus	:				G.	T.		G.		T.A.	A.	
South America consensus	:							G.				

		1220	*	1240	*	1260	*	1280	*	1300	*	1320
Consensus	:	TAAAAACGCACATGCCAAAAACAAGAAAGTAGTTGTCCTTGGATCCCAAGAGGGAGCAATGCATACCGCACTGACAGGAGCTACAGAGATCCAAAACCTCAGGAGGCACACA										
Africa consensus	:		T.			A.				C.		
Asia consensus	:		T.	A.				C.A.				
Europe consensus	:		A.	G.		A.		C.A.				C.
North America consensus	:			G.					T.		A.	

Oceania consensus : .....A.....A.....C.A.....C.....
South America consensus : .....T.....G.....A.....C.....A.....C.....

Consensus : AGTATTTTGCAGGGGCACTTAAATGTGAGACTTAAGATGGACAATTTGGAACCTCAAGGGGATGAGTATGCAATGTGCTTGAATACCTTTGTGTTGAAGAAAGAAAGTCTC
Africa consensus : .....C.....G.....AC.....
Asia consensus : .....C.....G.....C.....
Europe consensus : .....C.....T.....AC.....G.....C.....
North America consensus : .....C.....T.....AC.....G.....
Oceania consensus : .....G.....C.....G.....
South America consensus : .....AC.....G.....

Consensus : AGAAACGCAGCATGGGACATACTCATTAAAGTTGAGTACAAAGGGGAAGATGACCTTGAAGATTCTTTCTCCACAGAGGATGGACAAGGGAAAGCTCACAATGGCA
Africa consensus : .....G.....C.....
Asia consensus : .....N.....G.....C.....
Europe consensus : .....T.....A.....G.....N.....G.....C.....
North America consensus : .....C.....T.....
Oceania consensus : .....T.....A.....G.....C.....
South America consensus : .....C.....

Consensus : GACTGATCAGACCAACCCAATGTGACTAAGAAGGAGAGCCTGTAAATATGAGGCTGAACCTCCTTTTGGGGAAAGTAATATAGTAATTGGAATTGGAGACAAAGCC
Africa consensus : .....T.....C.....T.....
Asia consensus : .....C.....C.....C.....
Europe consensus : .....T.....A.....A.....C.....C.....C.....A.....
North America consensus : .....T.....C.....C.....C.....G.....C.....
Oceania consensus : .....T.....C.....A.....G.....
South America consensus : .....T.....C.....G.....C.....

Consensus : TTGAAATCAATGGGTACAAGAAGGGAAGCTCTATTGGGAAGATGTTTCGAGGCACTGCCAGAGGTGCAAGGCGCATGGCCATCTTGGGAGACACAGCTTGGGACTTTGG
Africa consensus : .....T.....
Asia consensus : .....G.....C.....
Europe consensus : .....G.....N.....N.....
North America consensus : .....T.....T.....
Oceania consensus : .....T.....G.....C.....
South America consensus : .....T.....C.....

Consensus : ATCAGTGGGTGGTGTCTGAATTATTAGGCAAAATGGTGACCAAAATATTGCGAAGTGCTTACACGCCCTATTAGTGGAGTCTCTGGGTATGAAAATTGGAATAG
Africa consensus : .....C.....G.....
Asia consensus : .....A.....G.....N.....A.....
Europe consensus : .....A.....T.....A.....A.....T.....A.....
North America consensus : .....C.....C.....C.....T.....
Oceania consensus : .....A.....T.....A.....G.....T.....G.....T.....A.....
South America consensus : .....C.....C.....T.....C.....

Consensus : GTGTCTCTTGACTTGGATAGGGTTGAAATCAAAAAACACTTCCATGTTCATTTTCATGCATTGTGATAGGAATCATATACACTCTATCTGGGAGCCGTGGTGCAAGCTGAC
Africa consensus : .....A.....C.....C.....A.....A.....
Asia consensus : .....C.....A.....C.....C.....A.....
Europe consensus : .....C.....T.....A.....C.....T.....N.....N.....
North America consensus : .....A.....C.....A.....T.....
Oceania consensus : .....C.....T.....A.....C.....T.....
South America consensus : .....A.....A.....C.....T.....

Consensus : ATGGGTGTGTGCATAAAATGGAAAGGCAAGAACTAAATGGGAAGTGAATTTTCGTACTAATGAGGTCCATACCTGGACAGACCAATACAAATTTCAAGCGGAATC
Africa consensus : .....T.....C.....C.....G.....C.....T.....
Asia consensus : .....C.....C.....C.....A.....
Europe consensus : .....N.....C.....C.....
North America consensus : .....C.....C.....C.....A.....C.....A.....
Oceania consensus : .....T.....C.....C.....C.....
South America consensus : .....A.....T.....C.....C.....C.....C.....A.....

Consensus : CCCAAAAGATTGGCGACAGCAATTGCAGGCGCTGGGAGAAATGGAGTGTGCGGAATTAGGTCAACAACCAGAATGGAGAATCTCTTGTGGAAGCAATATAGCCAATGAAC
Africa consensus : .....T.....A.....G.....

**Supplementary Figure 2. Alignment of D191267 and DV3syn ORFs
with the consensus ORFs of DENV-3 isolates from different continents.**

		*	40	*	80	*
D191267	:	atgaacaaccaacggaaaaagacgggaaaaccgtctatcatatgctgaaacgcgtgagaaacccgtgtgtcaactggatcacagttggcgaagagattct	:	100		
DV3syn ORF	:	.....	:	100		
G28H (MW720883.1)	:	.....	:	100		
Africa DENV-3 ORF	:	.....g.....	:	100		
Asia DENV-3 ORF	:	.....	:	100		
Europe DENV-3 ORF	:	.....	:	100		
North America DENV-3 ORF	:	.....g.....	:	100		
The Oceania DENV-3 ORF	:	.....	:	100		
South America DENV-3 ORF	:	.....g.....	:	100		

		120	*	160	*	200
D191267	:	caaaaggattgctgaacggccagggaacaaatgaaattggtcatggcgttcatagccttccttagatttctggccattccacaaacagcaggagttttggc	:	200		
DV3syn ORF	:	.....	:	200		
G28H (MW720883.1)	:	.....	:	200		
Africa DENV-3 ORF	:	.....t.....	:	200		
Asia DENV-3 ORF	:	..g.....a.....t.....t.....c.....a.....c.....	:	200		
Europe DENV-3 ORF	:	..g.....a.....t.....t.....c.....a.....g.....c.....	:	200		
North America DENV-3 ORF	:	.....t.....t.....c.....a.....c.....	:	200		
The Oceania DENV-3 ORF	:	..g.....t.....a.....t.....t.....c.....a.....g.....g.....c.....	:	200		
South America DENV-3 ORF	:	.....c.....t.....t.....t.....c.....a.....c.....	:	200		

		*	240	*	280	*
D191267	:	cagatggggaaccttcaagaagtcggggccattaaaggttctgaaaggcttcaagaaggagatttcaaacatgctgagcataatcaacaaacggaaaaag	:	300		
DV3syn ORF	:	.....	:	300		
G28H (MW720883.1)	:	.....	:	300		
Africa DENV-3 ORF	:	.....c.....	:	300		
Asia DENV-3 ORF	:	t.....t.....c..a.....t.....a.....	:	300		
Europe DENV-3 ORF	:	t.....t.....c...g.....c.....t.....g.....	:	300		
North America DENV-3 ORF	:	t.....c.....c.....c.....	:	300		
The Oceania DENV-3 ORF	:	t.....t.....c...g.....c.....t.....t.....g.....	:	300		
South America DENV-3 ORF	:	t.....c.....c.....c.....c.....	:	300		

		320	*	360	*	400
D191267	:	acatcgctctgtctcatgatgatattgccagcagcacttgccttccacttgacttcacgagatggagagccgcgcgatgattgtggggaagaatgaaagag	:	400		
DV3syn ORF	:	.....c.....	:	400		
G28H (MW720883.1)	:	.....	:	400		
Africa DENV-3 ORF	:	.....c.....	:	400		
Asia DENV-3 ORF	:	..g..a...a.....	:	400		
Europe DENV-3 ORF	:	..g..a...a.....t.....g.....	:	400		
North America DENV-3 ORF	:	.....	:	400		
The Oceania DENV-3 ORF	:	..g..a...a.....t.....g.....	:	400		
South America DENV-3 ORF	:	.....	:	400		

		*	440	*	480	*
D191267	:	gaaaatccctactttttaagacagcctctggaatcaacatgtgcacactcatagccatggactgggagaaatgtgtgatgatacggtcacttacaaatg	:	500		
DV3syn ORF	:	.g.....	:	500		
G28H (MW720883.1)	:	.....g.....c.....	:	500		
Africa DENV-3 ORF	:	.....g.....c.....	:	500		
Asia DENV-3 ORF	:	.n.....t.....g.....c.....	:	500		
Europe DENV-3 ORF	:	.....c.....t.....g.....c.....	:	500		
North America DENV-3 ORF	:	.....t.....g.....c.....	:	500		
The Oceania DENV-3 ORF	:	.....t.....g.....c.....	:	500		
South America DENV-3 ORF	:	.....t.....g.....c.....	:	500		

		520	*	560	*	600
D191267	:	ccccacattaccgaagtggaaacctgaagacattgactgctggtgcaaccttacatcaacatgggtgacttatggaacgtgcaatcaagctggagagcat	:	600		
DV3syn ORF	:	.....t.....	:	600		
G28H (MW720883.1)	:	.....g.....	:	600		
Africa DENV-3 ORF	:	.....c.....	:	600		
Asia DENV-3 ORF	:	...t...g.....g.....g.....g.....	:	600		
Europe DENV-3 ORF	:	...t...t...g.....g.....c...c.....c.....	:	600		
North America DENV-3 ORF	:	.....c.....c.....	:	600		
The Oceania DENV-3 ORF	:	...t...t...g.....g...g...c...c.....	:	600		
South America DENV-3 ORF	:	.....c.....	:	600		

		*	640	*	680	*
D191267	:	agacgcgacaagagatcagtggttagctcccatgtcgcatgggactggacacacgcacccaaacctggatgtcggtgaaggagcttggagacaag	:	700		
DV3syn ORF	:	.....	:	700		
G28H (MW720883.1)	:	.....	:	700		
Africa DENV-3 ORF	:	.....	:	700		
Asia DENV-3 ORF	:	.....	:	700		
Europe DENV-3 ORF	:	.....t.....g.....a.....g.....	:	700		
North America DENV-3 ORF	:	.....t.....g.....a.....g.....	:	700		
The Oceania DENV-3 ORF	:	.....t.....g.....a.....g.....	:	700		
South America DENV-3 ORF	:	.....a.....g.....	:	700		

		720	*	760	*	800
D191267	:	tcgagaaggtagagacatggggcccttaggtaccagggttcaccatactagccctatttcttggccattacataggcacctccttgaccagaaaggtggt	:	800		
DV3syn ORF	:	.....c.....	:	800		
G28H (MW720883.1)	:	.....	:	800		
Africa DENV-3 ORF	:	.....c.....c.....	:	800		
Asia DENV-3 ORF	:	.....c.....t.....t.....c.....a.....	:	800		
Europe DENV-3 ORF	:	.....t.....c.....a.....t.....t.....a.....	:	800		
North America DENV-3 ORF	:	.....c.....a.....	:	800		
The Oceania DENV-3 ORF	:	.....t.....c.....a.....t.....a.....	:	800		
South America DENV-3 ORF	:	.....c.....a.....	:	800		

		*	840	*	880	*
D191267	:	tatttttatactactaatgctgtgtcaccccatccatgacaatgagatgtgtgggagtgaggaaacagagattttgtggaaaggtctgtcaggagctacgtgg	:	900		
DV3syn ORF	:	.....a.....a.....	:	900		
G28H (MW720883.1)	:	.....	:	900		
Africa DENV-3 ORF	:	.....g.....a.....	:	900		
Asia DENV-3 ORF	:	.....t.....c.....a.....c.....	:	900		
Europe DENV-3 ORF	:	.....c.....a.....c.....a.....	:	900		
North America DENV-3 ORF	:	.....a.....a.....	:	900		
The Oceania DENV-3 ORF	:	.....c.....a.....c.....a.....	:	900		
South America DENV-3 ORF	:	.....a.....a.....	:	900		

		920	*	960	*	1000
D191267	:	gttgacgtggtgctagagcacgggggtgtgtgaccacatggctaagaacaagccacactggatagagcttcagaagaccgagggccaccaactgg	:	1000		
DV3syn ORF	:	.....t.....gt.....	:	1000		
G28H (MW720883.1)	:	.....	:	1000		
Africa DENV-3 ORF	:	.....g.....	:	1000		

Asia DENV-3 ORF : .....C.....t.....g....C..... : 1000
Europe DENV-3 ORF : .....C.....t.....t.....gt.....C..... : 1000
North America DENV-3 ORF : .....C.....t.....a.....gt.....t..... : 1000
The Oceania DENV-3 ORF : .....C.....t.....t.....gt.....C..... : 1000
South America DENV-3 ORF : .....C.....t.....a.....gt.....t..... : 1000

* 1040 * 1080 *
D191267 : cgaccctaaggaagctatgcattgaggggaaaattactaacatacaactgactcaagatgtcctaccacagggggaagcgggttttgcctgaggaacagga : 1100
DV3syn ORF : .....C.....g.....a.....g..... : 1100
G28H (MW720883.1) : .....C..... : 1100
Africa DENV-3 ORF : .....C.....C.....g..... : 1100
Asia DENV-3 ORF : .....t.....a.....g.....a.....a.....g..... : 1100
Europe DENV-3 ORF : .....n.....t.....a.....C.....ng.....C.....g.....C.....a.....a.....g..... : 1100
North America DENV-3 ORF : .....a.....t.....a.....C.....g.....g.....C.....g.....C.....a.....a.....g..... : 1100
The Oceania DENV-3 ORF : .....a.....t.....a.....C.....g.....g.....C.....g.....C.....a.....a.....g..... : 1100
South America DENV-3 ORF : .....C..... : 1100

1120 * 1160 * 1200
D191267 : ccagaactacgtgtgtaagcacacatacgttagatagaggctgggggaacggttggtgtgtttgttggcaagggaagccttgtaaacatgtgcgaaatttcaa : 1200
DV3syn ORF : .....t.....g.....C.....g..... : 1200
G28H (MW720883.1) : ..... : 1200
Africa DENV-3 ORF : .....t.....C..... : 1200
Asia DENV-3 ORF : .....a.....g.....t.....a.....a.....g.....C..... : 1200
Europe DENV-3 ORF : .....g.....C.....a.....C.....C..... : 1200
North America DENV-3 ORF : .....t.....a.....a..... : 1200
The Oceania DENV-3 ORF : .....g.....C.....a..... : 1200
South America DENV-3 ORF : .....t.....C.....a..... : 1200

* 1240 * 1280 *
D191267 : tgcctggaaccaatagagggaaaagttagtgcaatatgagaacctcaaatcacacgctcatcattacagtgcacacaggagaccaacaccaggtgggaaatg : 1300
DV3syn ORF : .....g.....C.....t..... : 1300
G28H (MW720883.1) : ..... : 1300
Africa DENV-3 ORF : .....g.....g.....t..... : 1300
Asia DENV-3 ORF : .....t.....a.....g.....C..... : 1300
Europe DENV-3 ORF : .....tt.....t.....ng.....gc.....C.....t..... : 1300
North America DENV-3 ORF : ..... : 1300
The Oceania DENV-3 ORF : .....tt.....t.....g.....gc.....C.....t..... : 1300
South America DENV-3 ORF : ..... : 1300

1320 * 1360 * 1400
D191267 : aaacgcagggaattacggctgagataacgcctcaggcatcaactactgaagccatcttgacctgaatatggaacccttgggctagaatgctcaccacggac : 1400
DV3syn ORF : .....g.....C.....a.....cgt..... : 1400
G28H (MW720883.1) : ..... : 1400
Africa DENV-3 ORF : .....g.....C..... : 1400
Asia DENV-3 ORF : .....g.....C.....a.....C.....cgt..... : 1400
Europe DENV-3 ORF : .....g.....C.....n.....C.....cgt..... : 1400
North America DENV-3 ORF : .....g.....C.....a.....C..... : 1400
The Oceania DENV-3 ORF : .....g.....C.....a.....C.....cgtc..... : 1400
South America DENV-3 ORF : .....g.....C.....a.....C..... : 1400

* 1440 * 1480 *
D191267 : aggtttggacttcaatgaaatgatcttactaacatgaagaacaaagcatggatggtacacagacaatggttttttgacctacctctaccatggacatca : 1500
DV3syn ORF : .....t.....t.....g.....t..... : 1500
G28H (MW720883.1) : ..... : 1500
Africa DENV-3 ORF : ..... : 1500
Asia DENV-3 ORF : .....t.....t.....g.....t.....C..... : 1500
Europe DENV-3 ORF : .....c.....a.....t.....t.....gt.....g.....t..... : 1500
North America DENV-3 ORF : .....t..... : 1500
The Oceania DENV-3 ORF : .....t.....t.....gt.....g.....t..... : 1500
South America DENV-3 ORF : .....t.....g.....t..... : 1500

1520 * 1560 * 1600
D191267 : ggagctacaacagaaacaccaacttggaaacaggaagagctccttgtgacattcaaaaaatgcacatgcgaaaaaacaagaagttagttgtccttggatcgc : 1600
DV3syn ORF : .....C.....t..... : 1600
G28H (MW720883.1) : ..... : 1600
Africa DENV-3 ORF : ..... : 1600
Asia DENV-3 ORF : .....g.....a.....t.....a.....g..... : 1600
Europe DENV-3 ORF : .....C.....t.....a.....t.....C.....a.....g.....a..... : 1600
North America DENV-3 ORF : .....g..... : 1600
The Oceania DENV-3 ORF : .....g.....C.....t.....a.....t.....C.....a.....g.....a..... : 1600
South America DENV-3 ORF : .....g.....C.....a.....t.....t.....C.....g..... : 1600

* 1640 * 1680 *
D191267 : aagagggagcaatgcatactgcaactgacaggagccacagaaatccaaaactcaggaggcacaagcatttttgcggggcacttaaaagtgtagacttaagat : 1700
DV3syn ORF : .....C.....g.....t.....C..... : 1700
G28H (MW720883.1) : ..... : 1700
Africa DENV-3 ORF : .....C..... : 1700
Asia DENV-3 ORF : .....C.....a.....g.....t.....g.....C..... : 1700
Europe DENV-3 ORF : .....C.....a.....t.....g.....C.....t..... : 1700
North America DENV-3 ORF : .....C.....t..... : 1700
The Oceania DENV-3 ORF : .....C.....a.....t.....g.....C..... : 1700
South America DENV-3 ORF : .....C.....t..... : 1700

1720 * 1760 * 1800
D191267 : ggataaactggaactcaaggggatgagctatgcaatgtgcacgaacacctttgtgttgaaagaagaagtctcagaaacgcagcatgggacaataactcatt : 1800
DV3syn ORF : .....C.....tt..... : 1800
G28H (MW720883.1) : ..... : 1800
Africa DENV-3 ORF : .....C.....t.....t.....g..... : 1800
Asia DENV-3 ORF : .....C.....t.....tt.....t..... : 1800
Europe DENV-3 ORF : .....C.....t.....tt.....tg.....C.....t.....a.....g..... : 1800
North America DENV-3 ORF : .....C.....t..... : 1800
The Oceania DENV-3 ORF : .....C.....t.....tt.....tg..... : 1800
South America DENV-3 ORF : .....C.....t..... : 1800

* 1840 * 1880 *
D191267 : aaggtcgagtacaaaggggaagatgcaccttgcaagattcctttctccacagaggatggacaagggaaagctcacaaatggcagactgatcacagccaacc : 1900
DV3syn ORF : .....t..... : 1900
G28H (MW720883.1) : ..... : 1900
Africa DENV-3 ORF : ..... : 1900
Asia DENV-3 ORF : .....t..... : 1900
Europe DENV-3 ORF : .....t.....n.....g.....C.....t..... : 1900
North America DENV-3 ORF : ..... : 1900
The Oceania DENV-3 ORF : .....t.....g.....C.....t..... : 1900
South America DENV-3 ORF : ..... : 1900

	1920	*	1960	*	2000
D191267	: cagtgggtgactaagaaggaggagcccgctcaacattgaggctgaacctccttttggggaaagtaacatagtaattggaattggagacaatgccttgaaaat	:	2000	:	2000
DV3syn ORF	:	:	2000	:	2000
G28H (MW720883.1)	:	:	2000	:	2000
Africa DENV-3 ORF	:	:	2000	:	2000
Asia DENV-3 ORF	:	:	2000	:	2000
Europe DENV-3 ORF	:	:	2000	:	2000
North America DENV-3 ORF	:	:	2000	:	2000
The Oceania DENV-3 ORF	:	:	2000	:	2000
South America DENV-3 ORF	:	:	2000	:	2000

	2040	*	2080	*
D191267	: caattggtacaagaagggaagctctattgggaagatgttcgaggccactgccagaggtgcaagacgcatggccatcttgggagacacagcttgggacttt	:	2100	:
DV3syn ORF	:	:	2100	:
G28H (MW720883.1)	:	:	2100	:
Africa DENV-3 ORF	:	:	2100	:
Asia DENV-3 ORF	:	:	2100	:
Europe DENV-3 ORF	:	:	2100	:
North America DENV-3 ORF	:	:	2100	:
The Oceania DENV-3 ORF	:	:	2100	:
South America DENV-3 ORF	:	:	2100	:

	2120	*	2160	*	2200
D191267	: ggatcagtggtgtgtctgaactcattagggcaaaatggtgcaccaaatattcggaagtgtctatacagccctattcagtgaggtctcttgggtgatga	:	2200	:	2200
DV3syn ORF	:	:	2200	:	2200
G28H (MW720883.1)	:	:	2200	:	2200
Africa DENV-3 ORF	:	:	2200	:	2200
Asia DENV-3 ORF	:	:	2200	:	2200
Europe DENV-3 ORF	:	:	2200	:	2200
North America DENV-3 ORF	:	:	2200	:	2200
The Oceania DENV-3 ORF	:	:	2200	:	2200
South America DENV-3 ORF	:	:	2200	:	2200

	2240	*	2280	*
D191267	: aaattggaataggtgttctcttaacttggataggggtgaattcaaaaaatcacatccatgtcattttcatgcattgcaataggaatcatcacactctattt	:	2300	:
DV3syn ORF	:	:	2300	:
G28H (MW720883.1)	:	:	2300	:
Africa DENV-3 ORF	:	:	2300	:
Asia DENV-3 ORF	:	:	2300	:
Europe DENV-3 ORF	:	:	2300	:
North America DENV-3 ORF	:	:	2300	:
The Oceania DENV-3 ORF	:	:	2300	:
South America DENV-3 ORF	:	:	2300	:

	2320	*	2360	*	2400
D191267	: gggagctgtgtgtacaagctgacatgggtgtgtcataaaatggaaaggcaagaacttaaatgcggaagtgaattttcgtcaccaacgaggtccatacc	:	2400	:	2400
DV3syn ORF	:	:	2400	:	2400
G28H (MW720883.1)	:	:	2400	:	2400
Africa DENV-3 ORF	:	:	2400	:	2400
Asia DENV-3 ORF	:	:	2400	:	2400
Europe DENV-3 ORF	:	:	2400	:	2400
North America DENV-3 ORF	:	:	2400	:	2400
The Oceania DENV-3 ORF	:	:	2400	:	2400
South America DENV-3 ORF	:	:	2400	:	2400

	2440	*	2480	*
D191267	: tggacagagcagtacaaattccaagcggattcccccataaagattggcgacagctattgcaggcgcttgggaaatggagtggtggaattaggtcaacaa	:	2500	:
DV3syn ORF	:	:	2500	:
G28H (MW720883.1)	:	:	2500	:
Africa DENV-3 ORF	:	:	2500	:
Asia DENV-3 ORF	:	:	2500	:
Europe DENV-3 ORF	:	:	2500	:
North America DENV-3 ORF	:	:	2500	:
The Oceania DENV-3 ORF	:	:	2500	:
South America DENV-3 ORF	:	:	2500	:

	2520	*	2560	*	2600
D191267	: ccagaatggagaatctcttgggaagcaaatagccaatgaactgaactacatattatgggaaaaacaacatcaaatcaacggtagtcgtggcgacacaat	:	2600	:	2600
DV3syn ORF	:	:	2600	:	2600
G28H (MW720883.1)	:	:	2600	:	2600
Africa DENV-3 ORF	:	:	2600	:	2600
Asia DENV-3 ORF	:	:	2600	:	2600
Europe DENV-3 ORF	:	:	2600	:	2600
North America DENV-3 ORF	:	:	2600	:	2600
The Oceania DENV-3 ORF	:	:	2600	:	2600
South America DENV-3 ORF	:	:	2600	:	2600

	2640	*	2680	*
D191267	: cggggttttagaacaaggaaaaagaacattaacaccacaacccatggagctaaaatactcatggaaaacgtggggaaggcaaaaaatagtgacagcagaa	:	2700	:
DV3syn ORF	:	:	2700	:
G28H (MW720883.1)	:	:	2700	:
Africa DENV-3 ORF	:	:	2700	:
Asia DENV-3 ORF	:	:	2700	:
Europe DENV-3 ORF	:	:	2700	:
North America DENV-3 ORF	:	:	2700	:
The Oceania DENV-3 ORF	:	:	2700	:
South America DENV-3 ORF	:	:	2700	:

	2720	*	2760	*	2800
D191267	: acacaaaaatcctctttcataatagacgggccaacacacccgagtgccaagtgcctcaagagcatggaatgtgtgggaggtggaagattacgggttcg	:	2800	:	2800
DV3syn ORF	:	:	2800	:	2800
G28H (MW720883.1)	:	:	2800	:	2800
Africa DENV-3 ORF	:	:	2800	:	2800
Asia DENV-3 ORF	:	:	2800	:	2800
Europe DENV-3 ORF	:	:	2800	:	2800
North America DENV-3 ORF	:	:	2800	:	2800
The Oceania DENV-3 ORF	:	:	2800	:	2800
South America DENV-3 ORF	:	:	2800	:	2800

	2840	*	2880	*
D191267	: gagtcttcacaaccaacatattgctgaaactccgagaggtgtacaccaaatgtgtgaccataggctaatgtcggcagccgtcaaggatgagagggccgt	:	2900	:
DV3syn ORF	:	:	2900	:
G28H (MW720883.1)	:	:	2900	:
Africa DENV-3 ORF	:	:	2900	:

Asia DENV-3 ORF : .....t.a.....a..... : 2900
Europe DENV-3 ORF : .....t.....a.....c.....n..... : 2900
North America DENV-3 ORF : .....c.a..... : 2900
The Oceania DENV-3 ORF : .....t.....c..... : 2900
South America DENV-3 ORF : .....c.a..... : 2900

2920 * 2960 * 3000
D191267 : gcatgccgacatgggctactggatagaaagccaaaagaatggaagtggagctagaaaaagcatccctcatagaggtgaaaacctgcacatggccaaaa : 3000
DV3syn ORF : ..... : 3000
G28H (MW720883.1) : ..... : 3000
Africa DENV-3 ORF : .....t..... : 3000
Asia DENV-3 ORF : .....t..... : 3000
Europe DENV-3 ORF : a..c.....t.....g.....a..... : 3000
North America DENV-3 ORF : a.....t..... : 3000
The Oceania DENV-3 ORF : a..c.....t.....g.....t.....a..... : 3000
South America DENV-3 ORF : a.....t..... : 3000

* 3040 * 3080 *
D191267 : tcacacactctttggagcaacgggtgtgctagagagtgacatgatcattccaaagagtctagctggctctatttcgcaacacaactacaggcccggtacc : 3100
DV3syn ORF : ..... : 3100
G28H (MW720883.1) : ..... : 3100
Africa DENV-3 ORF : ..... : 3100
Asia DENV-3 ORF : .....t.....a.....c.....c..... : 3100
Europe DENV-3 ORF : .....t.....t.....c.....g.....c.....a..... : 3100
North America DENV-3 ORF : .....t.....c.....t.....t.....c.....c..... : 3100
The Oceania DENV-3 ORF : .....t.....c.....t.....t.....g.....c.....g.....a..... : 3100
South America DENV-3 ORF : .....t.....c.....t.....t.....c.....c..... : 3100

3120 * 3160 * 3200
D191267 : acacccaaacggcaggaccctggcacttaggaaaattggagctggacttcaactatttgtgaaggaacaacagttgtcatcacagaaaattgtgggacaag : 3200
DV3syn ORF : ..... : 3200
G28H (MW720883.1) : ..... : 3200
Africa DENV-3 ORF : ..... : 3200
Asia DENV-3 ORF : .....g..... : 3200
Europe DENV-3 ORF : ..... : 3200
North America DENV-3 ORF : .....g.....g.....t.....a.....c..... : 3200
The Oceania DENV-3 ORF : ..... : 3200
South America DENV-3 ORF : .....g.....g.....t.....a.....c..... : 3200

* 3240 * 3280 *
D191267 : aggccccatttgagaacaacaacagtgctcaggaaaagtgatacatgaatggtgtgtgccgtcgtgcacacttcctccctgcgatacatgggagaagac : 3300
DV3syn ORF : ..... : 3300
G28H (MW720883.1) : ..... : 3300
Africa DENV-3 ORF : .....g.....c.....t..... : 3300
Asia DENV-3 ORF : .....g.....c.....t..... : 3300
Europe DENV-3 ORF : .....c.....g.....c.....t.....g..... : 3300
North America DENV-3 ORF : .....g.....a.....c.....t..... : 3300
The Oceania DENV-3 ORF : .....c.....g.....c.....t.....g.....t..... : 3300
South America DENV-3 ORF : .....g.....a.....c.....t..... : 3300

3320 * 3360 * 3400
D191267 : ggctgttggtatggcatggaatcagacccatcagtgagagaagaagagaacatggttaaagtctttagtctcagcgggaagtggaaaggtagacaacttta : 3400
DV3syn ORF : ..... : 3400
G28H (MW720883.1) : ..... : 3400
Africa DENV-3 ORF : .....t.....c..... : 3400
Asia DENV-3 ORF : .....c.....t.....a.....a.....g.....g..... : 3400
Europe DENV-3 ORF : .....t.....t.....a.....a.....g.....c.....g.....t.....c..... : 3400
North America DENV-3 ORF : .....t.....c.....a.....g.....g..... : 3400
The Oceania DENV-3 ORF : .....c.....t.....t.....a.....a.....g.....g.....t.....c..... : 3400
South America DENV-3 ORF : .....t.....c.....t.....a.....g.....g.....c..... : 3400

* 3440 * 3480 *
D191267 : caatgggtgtcttgtgtttggcaattctctttgaagaggtgatgagaggaaaatttgggaagaacacatgattgcagggtttttcttcacgtttgtgct : 3500
DV3syn ORF : ..... : 3500
G28H (MW720883.1) : ..... : 3500
Africa DENV-3 ORF : .....c..... : 3500
Asia DENV-3 ORF : .....n.....c.....c.....a.....c..... : 3500
Europe DENV-3 ORF : .....c.....a.....n.....c.....c..... : 3500
North America DENV-3 ORF : .....c.....g..... : 3500
The Oceania DENV-3 ORF : .....t.....c.....a.....g.....c..... : 3500
South America DENV-3 ORF : .....c.....g..... : 3500

3520 * 3560 * 3600
D191267 : ccttctctcagggcaaaataacatggagagacatggcgacacactaataatgattgggtccaacgcctctgacaggatgggaatgggcgtcacctatcta : 3600
DV3syn ORF : ..... : 3600
G28H (MW720883.1) : ..... : 3600
Africa DENV-3 ORF : ..... : 3600
Asia DENV-3 ORF : .....t.....c.....t.....c..... : 3600
Europe DENV-3 ORF : .....c.....c.....a.....t.....t.....c..... : 3600
North America DENV-3 ORF : .....a..... : 3600
The Oceania DENV-3 ORF : .....g.....c.....a.....a.....t.....t.....c..... : 3600
South America DENV-3 ORF : ..... : 3600

* 3640 * 3680 *
D191267 : gctttaattgcaacatttaaaatccagccattctctggcatttttcttaagaaaactgacatctagagaaaaattactgttaggagttggactgg : 3700
DV3syn ORF : ..... : 3700
G28H (MW720883.1) : ..... : 3700
Africa DENV-3 ORF : .....g..... : 3700
Asia DENV-3 ORF : .....t.....c.....g.....g.....n.....g.....gt..... : 3700
Europe DENV-3 ORF : .....a.....g.....n.....t.....c.....g.....g.....c.....n.....t.....c.....g.....gt..... : 3700
North America DENV-3 ORF : .....t.....t.....c.....g.....g.....c.....t.....t.....g..... : 3700
The Oceania DENV-3 ORF : .....a.....g.....t.....c.....g.....g.....t.....g.....gt..... : 3700
South America DENV-3 ORF : .....t.....c.....t.....c.....t.....g..... : 3700

3720 * 3760 * 3800
D191267 : ctatggcaacaacgttacaattgccagaggacattgaacaaatggcaaatggcctggggctcatggctcttaattgataacgcaatttgaaat : 3800
DV3syn ORF : ..... : 3800
G28H (MW720883.1) : ..... : 3800
Africa DENV-3 ORF : .....a.....c.....c.....a.....a..... : 3800
Asia DENV-3 ORF : .....c.....c.....g.....t.....t.....c.....c.....a.....g.....c..... : 3800
Europe DENV-3 ORF : .....c.....c.....c.....g.....a.....tt.....a.....a.....a.....c..... : 3800
North America DENV-3 ORF : .....c.....c.....c.....c.....a.....a.....a.....c..... : 3800
The Oceania DENV-3 ORF : .....c.....g.....c.....g.....a.....tt.....a.....a.....c..... : 3800
South America DENV-3 ORF : .....c.....c.....t.....c.....a.....a.....c..... : 3800

	5720	*	5760	*	5800	
D191267	: agatgtctaaaaccagtgatcctgacagatggaccagagcgggtgatcctggctggaccaatgccagtcaccgcggcgagtgctgcgcaaaggagagga :					5800
DV3syn ORF	:					5800
G28H (MW720883.1)	:					5800
Africa DENV-3 ORF	:					5800
Asia DENV-3 ORF	: .....c.g.....tt.....c.....a.....c..... :					5800
Europe DENV-3 ORF	: .....c.g.....tt.....c.....c.....g.....ta.....c.....a..... :					5800
North America DENV-3 ORF	: .....c.g.....tt.....c.....c.....c.....g.....ta.....c.....a..... :					5800
The Oceania DENV-3 ORF	: .....c.g.....tt.....c.....c.....c.....g.....ta.....c.....a..... :					5800
South America DENV-3 ORF	: .....c.....c.....c.....c.....c.....c.....c.....c.....c.....c..... :					5800

	5840	*	5880	*	
D191267	: gagggtggcaggaacccacaaaaagaaaatgaccagtacatattcacgggccagcctcttaataatgatgaagaccacgctcactggacagaagcaaaat :				5900
DV3syn ORF	:				5900
G28H (MW720883.1)	:				5900
Africa DENV-3 ORF	: .....c.c.....t.....c.....c.....c.....t..... :				5900
Asia DENV-3 ORF	: .....c.c.....c.....c.....c.....t.....t..... :				5900
Europe DENV-3 ORF	: .....g.....a.....c.....c.....c.....t.....t..... :				5900
North America DENV-3 ORF	: .....c.c.....c.....c.....c.....t.....t..... :				5900
The Oceania DENV-3 ORF	: .....g.....a.....c.....c.....c.....t.....t.....g..... :				5900
South America DENV-3 ORF	: .....c.c.....c.....c.....c.....t.....t.....t..... :				5900

	5920	*	5960	*	6000
D191267	: gctgctggacaacatcaacactccagaaggaatcataccagctctctttgaaccagaaaaggagaaagtcagccgcatagacgggtaatatcgctgaag :				6000
DV3syn ORF	:				6000
G28H (MW720883.1)	:				6000
Africa DENV-3 ORF	: .....t.....t.....t.....t.....t.....t.....t.....t.....t.....t..... :				6000
Asia DENV-3 ORF	: .....a.....a.....g.....g.....g.....g.....g.....g.....g.....g..... :				6000
Europe DENV-3 ORF	: .....n.a.....a.....g.....g.....g.....g.....g.....g.....g.....g..... :				6000
North America DENV-3 ORF	: .....t.....t.....t.....t.....t.....t.....t.....t.....t.....t.....a..... :				6000
The Oceania DENV-3 ORF	: .....t.a.....a.....g.....g.....g.....g.....g.....g.....g.....g..... :				6000
South America DENV-3 ORF	: .....t.....t.....t.....t.....t.....t.....t.....t.....t.....t.....g.....a..... :				6000

	6040	*	6080	*
D191267	: ggtgaatccaggaagactttcgtggaactcatgaggaggggtgacctccagtttggttagccacaaagtagcatcagaaggaatcaaatatacagata :			6100
DV3syn ORF	:			6100
G28H (MW720883.1)	:			6100
Africa DENV-3 ORF	: .....t.....t.....t.....t.....t.....t.....t.....t.....t.....t..... :			6100
Asia DENV-3 ORF	: .....g.....c.....c.....c.....c.....c.....c.....c.....c.....c.....c..... :			6100
Europe DENV-3 ORF	: .....g.....c.....c.....c.....c.....c.....c.....c.....c.....c.....c..... :			6100
North America DENV-3 ORF	: .....g.....t.....c.....c.....c.....c.....c.....c.....c.....c.....c..... :			6100
The Oceania DENV-3 ORF	: .....g.....c.....c.....c.....c.....c.....c.....c.....c.....c.....c..... :			6100
South America DENV-3 ORF	: .....g.....t.....c.....c.....c.....c.....c.....c.....c.....c.....c..... :			6100

	6120	*	6160	*	6200
D191267	: ggaaatggtgctttgatggacagcgcaacaatcaaattttagagaaaacatggcgtggaatctggacaaaggaaggagaaaaaataagacc :				6200
DV3syn ORF	:				6200
G28H (MW720883.1)	:				6200
Africa DENV-3 ORF	: .....a.....a.....a.....a.....a.....a.....a.....a.....a.....a..... :				6200
Asia DENV-3 ORF	: .....a.....g.....a.....t.....t.....g.....t.....t.....t.....t.....t..... :				6200
Europe DENV-3 ORF	: .....a.....g.....t.....g.....a.....t.....n.....g.....t.....t.....g.....g..... :				6200
North America DENV-3 ORF	: .....a.....g.....t.....g.....a.....t.....t.....g.....t.....t.....t.....g..... :				6200
The Oceania DENV-3 ORF	: .....a.....g.....t.....g.....t.....t.....g.....t.....t.....t.....g.....g..... :				6200
South America DENV-3 ORF	: .....a.....t.....a.....t.....t.....g.....t.....t.....t.....t.....t.....g..... :				6200

	6240	*	6280	*
D191267	: taggtggttgatgcccgcacttattcagatcccttagcactcaaggaatttaaggactttgcggctggcagaaaagtcacatcgccctagatcttgtgaca :			6300
DV3syn ORF	:			6300
G28H (MW720883.1)	:			6300
Africa DENV-3 ORF	: .....c.....c.....c.....c.....c.....c.....c.....c.....c.....c..... :			6300
Asia DENV-3 ORF	: .....c.....t.....a.....t.....t.....t.....t.....t.....t.....t.....t..... :			6300
Europe DENV-3 ORF	: .....a.....g.....c.....c.....c.....c.....c.....c.....c.....c.....c..... :			6300
North America DENV-3 ORF	: .....c.....c.....c.....c.....c.....c.....c.....c.....c.....c.....t..... :			6300
The Oceania DENV-3 ORF	: .....g.....c.....c.....c.....c.....c.....c.....c.....c.....c.....c..... :			6300
South America DENV-3 ORF	: .....c.....c.....c.....c.....c.....c.....c.....c.....c.....c.....t..... :			6300

	6320	*	6360	*	6400
D191267	: gaaataggaagagtgccttcacacctgacccacagaaacgctctggacaaatctggtgatgctgcacacgtcagaacatggcggtagggcctaca :				6400
DV3syn ORF	:				6400
G28H (MW720883.1)	:				6400
Africa DENV-3 ORF	: .....t.....t.....t.....t.....t.....t.....t.....t.....t.....t..... :				6400
Asia DENV-3 ORF	: .....t.....t.....t.....t.....t.....t.....t.....t.....t.....t.....g..... :				6400
Europe DENV-3 ORF	: .....t.....t.....t.....t.....t.....t.....t.....t.....t.....t.....g..... :				6400
North America DENV-3 ORF	: .....t.....t.....t.....t.....t.....t.....t.....t.....t.....t.....g..... :				6400
The Oceania DENV-3 ORF	: .....tt.....t.....t.....t.....t.....t.....t.....t.....t.....t.....g..... :				6400
South America DENV-3 ORF	: .....t.....t.....t.....t.....t.....t.....t.....t.....t.....t.....t..... :				6400

	6440	*	6480	*
D191267	: ggcacggtggaggaactaccagaacaatggaacactcctactcttggactcatgatcttactgacaggtggagcaatgctttcttgatcacagg :			6500
DV3syn ORF	:			6500
G28H (MW720883.1)	:			6500
Africa DENV-3 ORF	: .....a.....a.....a.....a.....a.....a.....a.....a.....a.....a..... :			6500
Asia DENV-3 ORF	: .....a.....c.....c.....c.....c.....c.....c.....c.....c.....c.....g..... :			6500
Europe DENV-3 ORF	: .....a.....g.....c.....c.....c.....c.....c.....c.....c.....c.....c.....g..... :			6500
North America DENV-3 ORF	: .....g.....c.....c.....c.....c.....c.....c.....c.....c.....c.....a..... :			6500
The Oceania DENV-3 ORF	: .....a.....t.....t.....t.....t.....t.....t.....t.....t.....t.....g..... :			6500
South America DENV-3 ORF	: .....g.....g.....g.....g.....g.....g.....g.....g.....g.....a..... :			6500

	6520	*	6560	*	6600
D191267	: taaagggattggaagacttcaataggactcatttgtgtgattgctccagcggcatgctgtggatggccgaattccactccaatggatcgctcggt :				6600
DV3syn ORF	:				6600
G28H (MW720883.1)	:				6600
Africa DENV-3 ORF	: .....c.....c.....c.....c.....c.....c.....c.....c.....c.....c..... :				6600
Asia DENV-3 ORF	: .....n.....t.....t.....t.....t.....t.....t.....t.....t.....t.....t..... :				6600
Europe DENV-3 ORF	: .....a.....g.....t.....t.....t.....t.....t.....t.....t.....t.....t.....c..... :				6600
North America DENV-3 ORF	: .....a.....t.....t.....t.....t.....t.....t.....t.....t.....t.....c..... :				6600
The Oceania DENV-3 ORF	: .....g.....a.....g.....t.....t.....t.....t.....t.....t.....t.....t.....c..... :				6600
South America DENV-3 ORF	: .....a.....a.....a.....a.....a.....a.....a.....a.....a.....a..... :				6600

	6640	*	6680	*
D191267	: atagtctcgaggttttttatgatggtgtgtgcttataccagaaccagaaaagcagagaaaccccaagacaaccaactgcgatgtcgtaataggcatac :			6700
DV3syn ORF	:			6700
G28H (MW720883.1)	:			6700
Africa DENV-3 ORF	: .....t.....t.....t.....t.....t.....t.....t.....t.....t.....t..... :			6700
Asia DENV-3 ORF	: .....t.....t.....t.....t.....t.....t.....t.....t.....t.....t..... :			6700

Asia DENV-3 ORF	: . . . . . a . . . . . c . . . . . t . . . . . g	: 6700
Europe DENV-3 ORF	: . . . . . n . . . . . c . . . . . a . . . . . t . . . . . g	: 6700
North America DENV-3 ORF	: . . . . c . . . . g	: 6700
The Oceania DENV-3 ORF	: . . . . c . . . . t . . . . . g	: 6700
South America DENV-3 ORF	: . . . . g	: 6700

	6720	*	6760	*	6800	
D191267	: ttacaatggctgcaataatagcggccaatgaaatgggactattggaaaccacaaagagagatctaggaatgtctaaggaaccagggtgtgtttctccaac	:	:	:	:	: 6800
DV3syn ORF	:	:	:	:	:	: 6800
G28H (MW720883.1)	:	:	:	:	:	: 6800
Africa DENV-3 ORF	: . . . . . t . . . . g . . . . . t . . . . g	:	:	:	:	: 6800
Asia DENV-3 ORF	: . . . . . c . . . . . . g . . . . . a . . . . . g . . . . . t . . . . a	:	:	:	:	: 6800
Europe DENV-3 ORF	: . . . . . gt . . . . . . g . . . . . a . . . . t . . . . . . c . . . . . a	:	:	:	:	: 6800
North America DENV-3 ORF	: . . . . . t . . . . a . . . . . t . . . . . gc . . . . . t . . . . g . . . . . c	:	:	:	:	: 6800
The Oceania DENV-3 ORF	: . . . . . gt . . . . g . . . . . a . . . . t . . . . . g . . . . . c . . . . . a	:	:	:	:	: 6800
South America DENV-3 ORF	: . . . . . t . . . . a . . . . t . . . . . g . . . . t . . . . . g . . . . . c	:	:	:	:	: 6800

	*	6840	*	6880	*	
D191267	: tagctatttggatgtggatttgcaccagcatcagcctggacattgtacgctgtggccactacagtaataacaccaatgttaagacataccatagagaat	:	:	:	:	: 6900
DV3syn ORF	:	:	:	:	:	: 6900
G28H (MW720883.1)	:	:	:	:	:	: 6900
Africa DENV-3 ORF	: . . . . c . . . . c . . . . c	:	:	:	:	: 6900
Asia DENV-3 ORF	: c . . . . . a . . . . . a . . . . . c . . . . c . . . . . a . . . . g	:	:	:	:	: 6900
Europe DENV-3 ORF	: c . . . . c . . . . g . . . . a . . . . c . . . . . g	:	:	:	:	: 6900
North America DENV-3 ORF	: c . . . . c . . . . c . . . . c	:	:	:	:	: 6900
The Oceania DENV-3 ORF	: c . . . . c . . . . g . . . . a . . . . g	:	:	:	:	: 6900
South America DENV-3 ORF	: c . . . . c . . . . c . . . . c	:	:	:	:	: 6900

	6920	*	6960	*	7000	
D191267	: tctacagcaaatgtgtccctggcagctatagccaaccaggcagtggtcctgatgggtttggacaaaggatggccaatatcaaaaatggacttaggcgtac	:	:	:	:	: 7000
DV3syn ORF	:	:	:	:	:	: 7000
G28H (MW720883.1)	:	:	:	:	:	: 7000
Africa DENV-3 ORF	:	:	:	:	:	: 7000
Asia DENV-3 ORF	: . . c . . . . c . . . . a . . . . g	:	:	:	:	: 7000
Europe DENV-3 ORF	: . . c . . . . a	:	:	:	:	: 7000
North America DENV-3 ORF	: . . . . t	:	:	:	:	: 7000
The Oceania DENV-3 ORF	: . . c . . . . a . . . . a . . . . g . . . . . g	:	:	:	:	: 7000
South America DENV-3 ORF	: . . . . t	:	:	:	:	: 7000

	*	7040	*	7080	*	
D191267	: cgctactggcattgggttgcctattcacaagtgaaccactgactctaacagcggcagtgacttttgcctagttacacattatgctattataggtccaggact	:	:	:	:	: 7100
DV3syn ORF	:	:	:	:	:	: 7100
G28H (MW720883.1)	: . a	:	:	:	:	: 7100
Africa DENV-3 ORF	: . a	:	:	:	:	: 7100
Asia DENV-3 ORF	: . a . . . . . . c . . . . . c . . . . c . . . . c . . . . t	:	:	:	:	: 7100
Europe DENV-3 ORF	: . a . . . . . t . . . . . c . . . . t . . . . . a . . . . cc . . . . . c . . . . . g . . . . . c . . . . . n . . . . . t	:	:	:	:	: 7100
North America DENV-3 ORF	: . c	:	:	:	:	: 7100
The Oceania DENV-3 ORF	: . a . . . . . t . . . . . c . . . . t . . . . . a . . . . . c . . . . . cc . . . . . c . . . . . g	:	:	:	:	: 7100
South America DENV-3 ORF	: . c	:	:	:	:	: 7100

	7120	*	7160	*	7200	
D191267	: gcaggcaaaagccactcgtgaagctcaaaaaggacagctgctggaataatgaagaatccaacgggtggatgggataataacaatagacctagacctgtga	:	:	:	:	: 7200
DV3syn ORF	:	:	:	:	:	: 7200
G28H (MW720883.1)	:	:	:	:	:	: 7200
Africa DENV-3 ORF	:	:	:	:	:	: 7200
Asia DENV-3 ORF	:	:	:	:	:	: 7200
Europe DENV-3 ORF	:	:	:	:	:	: 7200
North America DENV-3 ORF	:	:	:	:	:	: 7200
The Oceania DENV-3 ORF	:	:	:	:	:	: 7200
South America DENV-3 ORF	:	:	:	:	:	: 7200

	*	7240	*	7280	*	
D191267	: atatattgattcaaaaattgaaaagcaactgggacaggtcatgctcctggtttgtgtgcaggttcaactgttgttaatgagaacatcatgggccttgtgtg	:	:	:	:	: 7300
DV3syn ORF	:	:	:	:	:	: 7300
G28H (MW720883.1)	:	:	:	:	:	: 7300
Africa DENV-3 ORF	:	:	:	:	:	: 7300
Asia DENV-3 ORF	: . . . . . c . . . . c . . . . a . . . . . t . . . . . c . . . . t	:	:	:	:	: 7300
Europe DENV-3 ORF	: . . . . . c	:	:	:	:	: 7300
North America DENV-3 ORF	:	:	:	:	:	: 7300
The Oceania DENV-3 ORF	: . . . . . c	:	:	:	:	: 7300
South America DENV-3 ORF	:	:	:	:	:	: 7300

	7320	*	7360	*	7400	
D191267	: aagctttaacttttagctacaggaccaataacaacactctgggaaggatcacctgggaaattttggaataccacgatagctgttccatggcgaaacatttt	:	:	:	:	: 7400
DV3syn ORF	:	:	:	:	:	: 7400
G28H (MW720883.1)	:	:	:	:	:	: 7400
Africa DENV-3 ORF	: . . . . . c	:	:	:	:	: 7400
Asia DENV-3 ORF	: . . . . . c . . . . . cc . . . . . c . . . . a . . . . . g . . . . . c . . . . n . . . . . c	:	:	:	:	: 7400
Europe DENV-3 ORF	: . . . . . c . . . . . . nc . . . . . c	:	:	:	:	: 7400
North America DENV-3 ORF	: . . . . c	:	:	:	:	: 7400
The Oceania DENV-3 ORF	: . . . . . c . . . . . cc . . . . . c	:	:	:	:	: 7400
South America DENV-3 ORF	: . . . . c	:	:	:	:	: 7400

	*	7440	*	7480	*	
D191267	: tagagggagctattttggcaggagctgggcttgccttttctattatgaaatcagttggaacaggaaaaagaggaaacaggctctcaagggtgaaacttttagga	:	:	:	:	: 7500
DV3syn ORF	:	:	:	:	:	: 7500
G28H (MW720883.1)	:	:	:	:	:	: 7500
Africa DENV-3 ORF	: . . . . a	:	:	:	:	: 7500
Asia DENV-3 ORF	: . . . . a . . . . t . . . . . c . . . . g . . . . . g . . . . . g . . . . . a . . . . . c	:	:	:	:	: 7500
Europe DENV-3 ORF	:	:	:	:	:	: 7500
North America DENV-3 ORF	: . . . . a . . . . t . . . . a	:	:	:	:	: 7500
The Oceania DENV-3 ORF	:	:	:	:	:	: 7500
South America DENV-3 ORF	: . . . . a . . . . t	:	:	:	:	: 7500

	7520	*	7560	*	7600	
D191267	: gaaaaatggaaaaagaattggaatcaattatcccgagagaggtttgacctttacaagaagtctggaatcactgaagttggatagacagaagccaaagaag	:	:	:	:	: 7600
DV3syn ORF	:	:	:	:	:	: 7600
G28H (MW720883.1)	:	:	:	:	:	: 7600
Africa DENV-3 ORF	: . . . . c . . . . a	:	:	:	:	: 7600
Asia DENV-3 ORF	: . . . . . g . . . . a . . . . g . . . . a	:	:	:	:	: 7600
Europe DENV-3 ORF	: . . . . . g	:	:	:	:	: 7600
North America DENV-3 ORF	: . . . . g . . . . . a . . . . a	:	:	:	:	: 7600
The Oceania DENV-3 ORF	: . . . . . g	:	:	:	:	: 7600
South America DENV-3 ORF	: . . . . a . . . . a . . . . a	:	:	:	:	: 7600

			*	7640	*	7680	*	
D191267	:	ggttgaaaagagagagaaacaacaccatgccgtgtccagggttagtgcaaaacttcaatggttgtggaaaggaacatgggtcattcccgagggaagagt	:	7700				
DV3syn ORF	:		:	7700				
G28H (MW720883.1)	:		:	7700				
Africa DENV-3 ORF	:		:	7700				
Asia DENV-3 ORF	:		:	7700				
Europe DENV-3 ORF	:		:	7700				
North America DENV-3 ORF	:		:	7700				
The Oceania DENV-3 ORF	:		:	7700				
South America DENV-3 ORF	:		:	7700				

				7720	*	7760	*	7800
D191267	:	catggacctgggctgtggaagaggaggtgtgtcatattactgtgcaggactgaaaaaagtcacagaagtgcaggatacaciaaaggcggtccaggacac	:	7800				
DV3syn ORF	:		:	7800				
G28H (MW720883.1)	:		:	7800				
Africa DENV-3 ORF	:		:	7800				
Asia DENV-3 ORF	:		:	7800				
Europe DENV-3 ORF	:		:	7800				
North America DENV-3 ORF	:		:	7800				
The Oceania DENV-3 ORF	:		:	7800				
South America DENV-3 ORF	:		:	7800				

			*	7840	*	7880	*	
D191267	:	gaagaaccagtgcctatgtcaacatatggatggaacatagtcacaaattaatgagtggaaaggatgtgttttatcttccgctgaaaagtgtgacactctgt	:	7900				
DV3syn ORF	:		:	7900				
G28H (MW720883.1)	:		:	7900				
Africa DENV-3 ORF	:		:	7900				
Asia DENV-3 ORF	:		:	7900				
Europe DENV-3 ORF	:		:	7900				
North America DENV-3 ORF	:		:	7900				
The Oceania DENV-3 ORF	:		:	7900				
South America DENV-3 ORF	:		:	7900				

				7920	*	7960	*	8000
D191267	:	tgtgtgacattggagaatcttcaccaagcccaacagtggaaagagcagaactataagagttttgaagatggttgaaccatggctgaaaaacaaccagtt	:	8000				
DV3syn ORF	:		:	8000				
G28H (MW720883.1)	:		:	8000				
Africa DENV-3 ORF	:		:	8000				
Asia DENV-3 ORF	:		:	8000				
Europe DENV-3 ORF	:		:	8000				
North America DENV-3 ORF	:		:	8000				
The Oceania DENV-3 ORF	:		:	8000				
South America DENV-3 ORF	:		:	8000				

			*	8040	*	8080	*	
D191267	:	ttgcattaaagtattgaacccttacatgccagccgtgattgagcacctagaaaagactgcaaaggaaacacggagggaatgcttgtgagaaatccactttca	:	8100				
DV3syn ORF	:		:	8100				
G28H (MW720883.1)	:		:	8100				
Africa DENV-3 ORF	:		:	8100				
Asia DENV-3 ORF	:		:	8100				
Europe DENV-3 ORF	:		:	8100				
North America DENV-3 ORF	:		:	8100				
The Oceania DENV-3 ORF	:		:	8100				
South America DENV-3 ORF	:		:	8100				

				8120	*	8160	*	8200
D191267	:	cggaaactccacgcacgaaatgtactggatatctaattggcacaggtaacattgtctcttcagttaacatggtatccagattgttactgaacaggttcacga	:	8200				
DV3syn ORF	:		:	8200				
G28H (MW720883.1)	:		:	8200				
Africa DENV-3 ORF	:		:	8200				
Asia DENV-3 ORF	:		:	8200				
Europe DENV-3 ORF	:		:	8200				
North America DENV-3 ORF	:		:	8200				
The Oceania DENV-3 ORF	:		:	8200				
South America DENV-3 ORF	:		:	8200				

			*	8240	*	8280	*	
D191267	:	tgacacacaggagacctaccatagagaaagatgtggatttaggagcaggaaactcgacatgttaatgcggaaccagaaacacccaacatggatgtcattgg	:	8300				
DV3syn ORF	:		:	8300				
G28H (MW720883.1)	:		:	8300				
Africa DENV-3 ORF	:		:	8300				
Asia DENV-3 ORF	:		:	8300				
Europe DENV-3 ORF	:		:	8300				
North America DENV-3 ORF	:		:	8300				
The Oceania DENV-3 ORF	:		:	8300				
South America DENV-3 ORF	:		:	8300				

				8320	*	8360	*	8400
D191267	:	ggaaagaataaaaaggatcaaggaggagcataattcaacatggccacatgatgacgaaaaacccctacaaaacgtgggcctaccacggatcttatgaagtc	:	8400				
DV3syn ORF	:		:	8400				
G28H (MW720883.1)	:		:	8400				
Africa DENV-3 ORF	:		:	8400				
Asia DENV-3 ORF	:		:	8400				
Europe DENV-3 ORF	:		:	8400				
North America DENV-3 ORF	:		:	8400				
The Oceania DENV-3 ORF	:		:	8400				
South America DENV-3 ORF	:		:	8400				

			*	8440	*	8480	*	
D191267	:	aaggccacaggctcagctcctccatgataaacggaggtcgtaaacttctcactaaacctgggatgtagtccccatgggtgacacagatggctatgacag	:	8500				
DV3syn ORF	:		:	8500				
G28H (MW720883.1)	:		:	8500				
Africa DENV-3 ORF	:		:	8500				
Asia DENV-3 ORF	:		:	8500				
Europe DENV-3 ORF	:		:	8500				
North America DENV-3 ORF	:		:	8500				
The Oceania DENV-3 ORF	:		:	8500				
South America DENV-3 ORF	:		:	8500				

				8520	*	8560	*	8600
D191267	:	atacaactccatttggccagcagagagtctttaagagaaggtggacaccaggacacccaggcctatgccagggacagaaggttatggagatcacagc	:	8600				
DV3syn ORF	:		:	8600				
G28H (MW720883.1)	:		:	8600				
Africa DENV-3 ORF	:		:	8600				
Asia DENV-3 ORF	:		:	8600				

```

Europe DENV-3 ORF : .....c.....c.....a.g.t.....a.....a..c.....a..... : 8600
North America DENV-3 ORF : .....c.....c.....a.....a.....a.....t.c.....a.....g..c...g..... : 8600
The Oceania DENV-3 ORF : .....c.t.....a.g.t.....a.....a.....a..c.....a..... : 8600
South America DENV-3 ORF : .....c.....c.....a.....a.....a.....t.c.....a.....g..c...g..... : 8600

D191267 : agagtggctctggagaacctgggaaggaacaaaagaccaggttatgcacaagggaagagttcacaaaaaggtcagaaccaacgcagcaatggcgcc : 8700
DV3syn ORF : .....c.....c.....a.....a.....a.....t.c.....a.....g..c...g..... : 8700
G28H (MW720883.1) : .....c.....c.....a.....a.....a.....t.c.....a.....g..c...g..... : 8700
Africa DENV-3 ORF : g.....t.....t.....a.g.....t.....c.....t..... : 8700
Asia DENV-3 ORF : g..a....t.....a.g.....g.....g..t..... : 8700
Europe DENV-3 ORF : g..a....t.....a.n..t.g.a.g.....tn...n.....t..... : 8700
North America DENV-3 ORF : g.....a.....t.....g.....t.....t..... : 8700
The Oceania DENV-3 ORF : g..a....t.....a.....t.g.a.g.....t.....t..... : 8700
South America DENV-3 ORF : g.....t.....a.....t.....t.....c..... : 8700

D191267 : gttttcacagagagaaccaatgggacagtgcgaagccgctgttgaggatgaagagttttggaaacttggtgacagagaaactgaactccacaaattgg : 8800
DV3syn ORF : .....c.....c.....a.....a.....a.....t.c.....a.....g..c...g..... : 8800
G28H (MW720883.1) : .....c.....c.....a.....a.....a.....t.c.....a.....g..c...g..... : 8800
Africa DENV-3 ORF : .....c.....c.....a.....a.....a.....t.c.....a.....g..c...g..... : 8800
Asia DENV-3 ORF : .....t.....g..t.....c.g.a.c..... : 8800
Europe DENV-3 ORF : .....t.....t.g..t.....n.....c.....c.....t..... : 8800
North America DENV-3 ORF : .....c.....t.....g..t..... : 8800
The Oceania DENV-3 ORF : .....t.....t.g..t.....a.c.....c.....t..... : 8800
South America DENV-3 ORF : .....c.....t.....g..t..... : 8800

D191267 : gcaagtgtggaagctgtgtttacaacatgatgggcaagagagagaagaaacttgagagtttgcaaagcaaaaggcagtagagctatatggtacatgtg : 8900
DV3syn ORF : .....c.....c.....a.....a.....a.....t.c.....a.....g..c...g..... : 8900
G28H (MW720883.1) : .....c.....c.....a.....a.....a.....t.c.....a.....g..c...g..... : 8900
Africa DENV-3 ORF : .....c.....c.....a.....a.....a.....t.c.....a.....g..c...g..... : 8900
Asia DENV-3 ORF : .....c.....c.....a.....a.....a.....t.c.....a.....g..c...g..... : 8900
Europe DENV-3 ORF : .....c.....c.....a.....a.....a.....t.c.....a.....g..c...g..... : 8900
North America DENV-3 ORF : .....c.....c.....a.....a.....a.....t.c.....a.....g..c...g..... : 8900
The Oceania DENV-3 ORF : .....c.....c.....a.....a.....a.....t.c.....a.....g..c...g..... : 8900
South America DENV-3 ORF : .....c.....c.....a.....a.....a.....t.c.....a.....g..c...g..... : 8900

D191267 : gttgggagccaggtaccttgagttcgaagccctggattcttaaatgaggaccactggttctcgcgtgaaaactcttatagtgagtagaaggagaagga : 9000
DV3syn ORF : .....c.....c.....a.....a.....a.....t.c.....a.....g..c...g..... : 9000
G28H (MW720883.1) : .....c.....c.....a.....a.....a.....t.c.....a.....g..c...g..... : 9000
Africa DENV-3 ORF : .....c.....c.....a.....a.....a.....t.c.....a.....g..c...g..... : 9000
Asia DENV-3 ORF : .....a..c.....a.....a.....a.....c..... : 9000
Europe DENV-3 ORF : .....a..c.....a.....a.....a.....c..... : 9000
North America DENV-3 ORF : .....a.....a.....a.....a.....g.....c.....g..... : 9000
The Oceania DENV-3 ORF : .....g..a..c.....a.....t.....c.....g..... : 9000
South America DENV-3 ORF : .....a.....a.....a.....g.....c.....g..... : 9000

D191267 : ctgcacaagctaggctacatattaaggacatttccaagatacccgaggagccatgtatgctgatgacacagctggttgggacacaagaataacagaag : 9100
DV3syn ORF : .....c.....c.....a.....a.....a.....t.c.....a.....g..c...g..... : 9100
G28H (MW720883.1) : .....c.....c.....a.....a.....a.....t.c.....a.....g..c...g..... : 9100
Africa DENV-3 ORF : .....g..a....t.....a.....a..... : 9100
Asia DENV-3 ORF : .....t.a.g..a....c.....a.n.....t..... : 9100
Europe DENV-3 ORF : .....t.....t..... : 9100
North America DENV-3 ORF : .....t.....g..a....c.g.a.t.....t..... : 9100
The Oceania DENV-3 ORF : .....t.....g..a....c.g.a.t.....t..... : 9100
South America DENV-3 ORF : .....t.....t.....t..... : 9100

D191267 : atgacctgcacaatgaggagaagatcacacagcagatggaccctgaacacaggctgttagcgaatgctatatttaagctcacataccaaaacaaagtgg : 9200
DV3syn ORF : .....c.....c.....a.....a.....a.....t.c.....a.....g..c...g..... : 9200
G28H (MW720883.1) : .....c.....c.....a.....a.....a.....t.c.....a.....g..c...g..... : 9200
Africa DENV-3 ORF : .....a.....a.....a.....c..... : 9200
Asia DENV-3 ORF : .....a.a.....a.....a.a.....c.c..... : 9200
Europe DENV-3 ORF : .....a.....a.....a.....a.....a.....c.....c..... : 9200
North America DENV-3 ORF : .....a.....a.....a.....a.c.....c..... : 9200
The Oceania DENV-3 ORF : .....a.....a.....a.....a.....a.....c..... : 9200
South America DENV-3 ORF : .....a.....a.....a.....a.....c..... : 9200

D191267 : caaagtccaacgaccgactccaacgggcacggttaatggacatcatatctaggaagaccaaagaggcagtggaacagtggaacttatggtctgaacaca : 9300
DV3syn ORF : .....c.....c.....a.....a.....a.....t.c.....a.....g..c...g..... : 9300
G28H (MW720883.1) : .....c.....c.....a.....a.....a.....t.c.....a.....g..c...g..... : 9300
Africa DENV-3 ORF : .....a.....aa.....g..... : 9300
Asia DENV-3 ORF : .....a.....a..... : 9300
Europe DENV-3 ORF : .....a.....a..... : 9300
North America DENV-3 ORF : .....a.....a..... : 9300
The Oceania DENV-3 ORF : .....a.....a..... : 9300
South America DENV-3 ORF : .....a.....a..... : 9300

D191267 : tttaaccaatggaagccagctagtgcagacaaatggaaggagaaggtgtgctgtcaaaaggcagacctcgagaacctcatctgccagaaaagaaat : 9400
DV3syn ORF : .....c.....c.....a.....a.....a.....t.c.....a.....g..c...g..... : 9400
G28H (MW720883.1) : .....c.....c.....a.....a.....a.....t.c.....a.....g..c...g..... : 9400
Africa DENV-3 ORF : .....t.....g.....c..t..g.....t.....g.....c..... : 9400
Asia DENV-3 ORF : .....c.....t.....t.....t.....g.n.....c...t..n...g... : 9400
Europe DENV-3 ORF : .....c.....t.a.....t.a..... : 9400
North America DENV-3 ORF : .....c.....t.ga.....t.....g.....t.....g..... : 9400
The Oceania DENV-3 ORF : .....c.....t.a.....t.....g..... : 9400
South America DENV-3 ORF : .....c.....t.a.....t.....g..... : 9400

D191267 : cacaatggctggaacccaaggagtgagagattaaaaagaatggccattagcggggatgattgcgtagtgaaccaatcgatgacaggttcgctaaccgc : 9500
DV3syn ORF : .....c.....c.....a.....a.....a.....t.c.....a.....g..c...g..... : 9500
G28H (MW720883.1) : .....c.....c.....a.....a.....a.....t.c.....a.....g..c...g..... : 9500
Africa DENV-3 ORF : .....t.....g.....c.....g.....g.....g.....t.c.t..... : 9500
Asia DENV-3 ORF : .....t.....a.....g.....c.....g.....g.....n.....c.t..... : 9500
Europe DENV-3 ORF : .....t.....a.....g.....c.....g.....g.....n.....c.t..... : 9500
North America DENV-3 ORF : .....t.....a.....g.....c.....g.....g.....n.....c.t..... : 9500
The Oceania DENV-3 ORF : .....t.....g.....c.....g.....g.....c.....t..... : 9500
South America DENV-3 ORF : .....t.....g.....c.....g.....g.....c.....t..... : 9500

```

	9520	*	9560	*	9600	
D191267	: cctgcttgctttgaacgatatgggaaaggttcggaagacatacctcaatggcagccatcaaaaggatggcatgactggcagcaggttcctttctgctcc	:		:	9600	
DV3syn ORF	:			:	9600	
G28H (MW720883.1)	:			:	9600	
Africa DENV-3 ORF	:			:	9600	
Asia DENV-3 ORF	: .....C.....g.....t.....a.....C.....	:		:	9600	
Europe DENV-3 ORF	: .....CC...t..C.....a...g.....t.....a.....C.....	:		:	9600	
North America DENV-3 ORF	: .....C...C.....g.....t.....a.....C.....	:		:	9600	
The Oceania DENV-3 ORF	: .....CC...t..C.....a...g.....g.....t.....a.....C.....	:		:	9600	
South America DENV-3 ORF	: .....C...C.....g.....t.....a.....C.....	:		:	9600	

	9640	*	9680	*	
D191267	: caccactttcatgaattgatcatgaaagatggaagaaagtgtggtggtccctgcagacccaggacgaactaataggaagagcaaggatctctcaaggag	:		:	9700
DV3syn ORF	:			:	9700
G28H (MW720883.1)	:			:	9700
Africa DENV-3 ORF	:			:	9700
Asia DENV-3 ORF	: .....C.....g.....a.....	:		:	9700
Europe DENV-3 ORF	: .....C.....t...t...C..g...n..a.....	:		:	9700
North America DENV-3 ORF	: .....C.....a.....	:		:	9700
The Oceania DENV-3 ORF	: .....t.....a.....t...t...t...C..g...t..a.....	:		:	9700
South America DENV-3 ORF	: .....C.....a.....	:		:	9700

	9720	*	9760	*	9800	
D191267	: cgggatggagccttagagaaaccgatgcctggggaaagcctatgcccaaatgtggagtctcatgtattttcacagaagagatctcagattagcgtccaa	:		:	9800	
DV3syn ORF	:			:	9800	
G28H (MW720883.1)	:			:	9800	
Africa DENV-3 ORF	: .....tt.....C.....	:		:	9800	
Asia DENV-3 ORF	: .a.....a.....t...t..a.....C..t.....t.....t...C...a.....	:		:	9800	
Europe DENV-3 ORF	: .a.....C.....t.....a.....C..n.....gc.....C.....t...C...a.....	:		:	9800	
North America DENV-3 ORF	: .....t.....t.....C..t.....C.....C.....C...a.....	:		:	9800	
The Oceania DENV-3 ORF	: .a.....t.....a.....C.....gc.....t...C...a.....	:		:	9800	
South America DENV-3 ORF	: .....t.....C..t.....C.....C...a.....	:		:	9800	

	9840	*	9880	*	
D191267	: tgccatatgttcagcagttaccagtccactgggtccctactagtagaacgacatggtctattcatgctcaccatcagtggtgaccacagaagacatgctt	:		:	9900
DV3syn ORF	:			:	9900
G28H (MW720883.1)	:			:	9900
Africa DENV-3 ORF	: .....a.....t.....	:		:	9900
Asia DENV-3 ORF	: C.....t.....t..a..C.....g.....t.....t.....	:		:	9900
Europe DENV-3 ORF	: C.....t.....t..a..C.....g.....t.....t.....	:		:	9900
North America DENV-3 ORF	: C.....t.....t..a..C.....g.....t.....t.....	:		:	9900
The Oceania DENV-3 ORF	: C.....t.....t..a..C.....g.....t.....t.....	:		:	9900
South America DENV-3 ORF	: C.....t.....t..a..C.....g.....t.....t.....	:		:	9900

	9920	*	9960	*	10000	
D191267	: actgtctggaacaggggtgtggatcgaggacaatccatggatggaagacaaaactccaattacaacctgggaaaaactccatcctaggggaagaggaag	:		:	10000	
DV3syn ORF	:			:	10000	
G28H (MW720883.1)	:			:	10000	
Africa DENV-3 ORF	: .....g.....t.....a.....	:		:	10000	
Asia DENV-3 ORF	: .....a.....g..C...t...g..t.....a.....	:		:	10000	
Europe DENV-3 ORF	: .....t.....a.....g.....g..Cn...g..t.....t.....a.....	:		:	10000	
North America DENV-3 ORF	: .....g..C...t...g..t.....t.....a.....	:		:	10000	
The Oceania DENV-3 ORF	: .....t.....a.....g.....g..C...t...g..t.....t.....a.....	:		:	10000	
South America DENV-3 ORF	: .....g..C...t...g..t.....t.....a.....	:		:	10000	

	10040	*	10080	*	
D191267	: accaatgggtgtggatcacttattgggtctcacttccagagcaacctggggccagacaacataccacagcaattcaacaggtgagaagccttataggcaatga	:		:	10100
DV3syn ORF	:			:	10100
G28H (MW720883.1)	:			:	10100
Africa DENV-3 ORF	: .....C.....	:		:	10100
Asia DENV-3 ORF	: .....C.....C.....t...g...C.....	:		:	10100
Europe DENV-3 ORF	: .....C.....C.....tt..g...C.....	:		:	10100
North America DENV-3 ORF	: .....C.....	:		:	10100
The Oceania DENV-3 ORF	: .....C.....C.....tt..g...C.....	:		:	10100
South America DENV-3 ORF	: .....C.....	:		:	10100

	10120	*	10160	
D191267	: agagtgttctggactacatgccttcaatgaagagattcaggaaggaggaggagtcggaggaggagccatttggttaa	:	10173	
DV3syn ORF	:	:	10173	
G28H (MW720883.1)	:	:	10173	
Africa DENV-3 ORF	:	:	10173	
Asia DENV-3 ORF	: .....g.....a.....	:	10173	
Europe DENV-3 ORF	: .....a..n.....	:	10173	
North America DENV-3 ORF	: .....t.....	:	10173	
The Oceania DENV-3 ORF	: .....a.....	:	10173	
South America DENV-3 ORF	: .....t.....	:	10173	

Supplementary Figure 3. Phylogenetic analysis of D191267 and DV3syn with the consensus DENV-3 ORF from different continents.

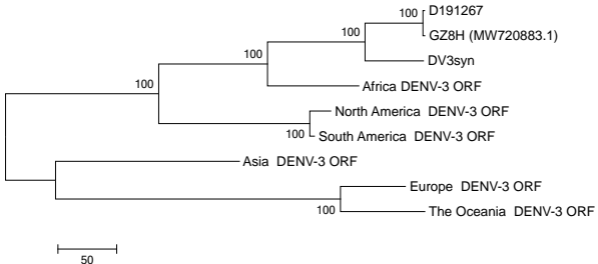

Supplementary Figure 4. Comparison of prM-E-NS1 sequences for D191267, consensus sequence, and the ECP-reduced sequence.

Figure 1 displays a comparison of the D191267 sequence (top) and the ECP-reduced sequence (bottom) across 12 panels, each showing a 20-nucleotide window. The Consensus sequence is shown in the middle. Asterisks (*) indicate positions where the D191267 sequence differs from the Consensus. Grey vertical bars highlight regions of high similarity between the D191267 and ECP-reduced sequences.

Panel 1 (Nucleotides 1-20): D191267: TTCCACTTGAC...TCACGAGATGGAGAGCCGCGCATGATTGTGGGGAAGAATGAAAGAGG...AAATCCCTACTTTTAAAGACAGCCTCTGGAATCAACATGTGCACACTCATAGCCATGGAC; Consensus: ...; ECP-reduced: ...C...G...

Panel 2 (Nucleotides 21-40): D191267: TTGGGAGAAATGTGTGATGATACGGTCACTTACAAATGCCCCACATTACCGAAGTGGAACCTGAAGACATTGACTGCTGGTGCAACCTTACATCAACATGGGTGACTTATGGAACGTGC; Consensus: ...; ECP-reduced: ...

Panel 3 (Nucleotides 41-60): D191267: AATCAAGCTGGAGAGCATAGACGCGACAAGAGATCAGTGGCGTTAGCTCCCCATGTCGGCATGGGACTGGACACACGCACCCAAACCTGGATGTCGGCTGAAGGAGCTTGGAGACAAGTC; Consensus: ...; ECP-reduced: ...

Panel 4 (Nucleotides 61-80): D191267: GAGAAGGTAGAGACATGGGCCCTTAGGCATCCAGGGTTCAACCATACTAGCCCTATTCTTGCCCATACATAGGCACCTCC...TGACCCAGAAGGTGGTTATTTTATACTACTAATGTGTG; Consensus: ...; ECP-reduced: ...C...

Panel 5 (Nucleotides 81-100): D191267: GTCACCCCATCCATGACAATGAGATGTGTGGGAGTGGGAAACAGAGATTTTGTGGAAGGCTCTGTGAGGAGCTACGTGGGTGACGTGGTGCCTAGAGACACGGGGGGTGTGTACCACCATG; Consensus: ...; ECP-reduced: ...

Panel 6 (Nucleotides 101-120): D191267: GCTAAGAACAAGCCACACTGGATATAGAGCTTCAGAAGACCGAGGCCACCAACTGGCGACCCCTAAGGAAGCTATGCATTGAGGGGAAAATTACTAACAATAACAACTGACTCAAGATGT; Consensus: ...GT...GT...; ECP-reduced: ...GT...GT...

Panel 7 (Nucleotides 121-140): D191267: CCTACCCAAGGGGAAGCGGTTTTCGCTGAGGAACAGGACCAGAACTACGTGTGTAAGCACACATACGTAGATAGAGGCTGGGGGAACGGTTGTGGTTTGTGGCAAGGGAAGCTTGGTA; Consensus: ...; ECP-reduced: ...A...G

Panel 8 (Nucleotides 141-160): D191267: ACATGTGCGAAATTTCAATGCCTGGAACCAATAGAGGGAAAAAGTAGTGC AATATGAGAACCTCAAATACACCGTCATCATTACAGTGCACACAGGAGACCAACACCAGGTGGGAAATGAA; Consensus: ...; ECP-reduced: ...

Panel 9 (Nucleotides 161-180): D191267: ACGCAGGGAATTACGGCTGAGATAACGCCTCAGGCATCAACTACTGAAGCCATCTT...CCTGAATATGGAACCTTGGGCTAGAATGCTCACCACGGACAGGTTTGGACTTCAATGAAATG; Consensus: ...C...C...; ECP-reduced: ...C...C...

Panel 10 (Nucleotides 181-200): D191267: ATCTTA...TACAATGAAGAACAAGCATGGATGGTACACAGACAATGGTTTTTGACCTACCTCTACCATGGACATCAGGAGCTACAACAGAAACCAACCTTGAACAGGAAGGAGCTC; Consensus: ...T...G...; ECP-reduced: ...G...G...

Panel 11 (Nucleotides 201-220): D191267: AGCAT...TTTGGCGGGCACTTAAAGTGTAGACTTAAGATGGATAAA...TGGAACTCAAGGGGATGAGCTATGCAATGTGCACGAACACCTTTGTGTTGAAGAAAGAAGTCTCAGAAACGCAG; Consensus: ...T...T...; ECP-reduced: ...C...T...

Panel 12 (Nucleotides 221-240): D191267: CTTGTGACATTCAAAAATGCACATGCGAAAAACAAGAAGTAGTTGTGCTTGGATCGCAAGAGGGAGCAATGCATACTGCACTGACAGGAGCCACAGAAATCCAAAACTCAGGAGGCACA; Consensus: ...; ECP-reduced: ...

Supplementary Table 1. Primers used for RT-PCR of DENV-3 isolate, D191267

Primers	Position	Sequence (5'-3')	Product length (nt)
F54	54-76	AACGTAGTGCTAACAGTTTTTTA	3002
R3055	3031-3055	TTTTTCTAGCTTCCAAC TTCCATTC	
F2836	2836-2859	GGAGTGTCCAAGTGCCTCAAGAGC	3117
R5952*	5929-5952	AGAGGCTGGCCCGTGAATATGTAC	
F5812	5812-5832	GATCCTGACAGATGGACCAGA	3043
R8854	8834-8854	AAACTCTTCATCCTCAACAGC	
F8740	8740-8759	ATGCACAAGGGAAGAGTTCA	1884
R10623*	10604-10623	CTCTCCCAGCGTCAATATGC	

*, these primers were used for reverse transcription.

Supplementary Table 2. Modification of ECP sites in the DENV-3 consensus prM-E-NS1 sequence.

Predicted<br>ECP	Start<br>position	End<br>position	Original<br>score	Modified<br>position	Substitution, codon<br>(aa)	Final ECP<br>score
1	440	485	0.96	448	ACT-ACC (Thr-Thr)	< 0.8
2	466	511	0.84	496	GGA-GGG (Gly-Gly)	< 0.8
3	872	917	0.94	878	TTG-CTG (Leu-Leu)	< 0.8
4	1246	1291	0.93	1276	GTA-GTG (Val-Val)	< 0.8
5	1447	1492	0.98	1453	TTG-TTA (Leu-Leu)	0.83
6	1496	1541	0.97	1523-1525	TTG-CTA (Leu-Leu)	< 0.8
7	1517	1562	0.91			
8	1760	1805	0.95	1762	ATT-ATC (Ile-Ile)	< 0.8
9	1769	1814	0.82	1802	TTG-CTG (Leu-Leu)	< 0.8
10	1796	1841	0.89			
11	1995	2040	0.89	2005	ACT-ACC (Thr-Thr)	< 0.8
12	2022	2067	0.91	2056	AGT-AGC (Ser-Ser)	< 0.8
13	2065	2110	0.98	2065-2068	GTA-GTG (Val-Val)	< 0.8
					ATT-ATC (Ile-Ile)	
14	2296	2341	0.97	2299	ATT-ATC (Ile-Ile)	< 0.8
15	2354	2399	0.87	2383	ATT-ATC (Ile-Ile)	< 0.8
16	2535	2580	0.97	2537	TTG-CTG (Leu-Leu)	< 0.8
17	2608	2653	0.82	2612	TTG-CTG (Leu-Leu)	< 0.8
18	2750	2795	0.95	2752	TAT-TAC (Tyr-Tyr)	0.85
19	2783	2828	0.86	2791	GCT-GCA (Ala-Ala)	< 0.8

Supplementary Table 3. The divergence table of D191267 and DV3syn ORFs in alignment with the consensus ORF of DENV-3 isolates from different continents.

	Africa DENV-3 ORF	Asia DENV-3 ORF	D191267	DV3syn WT ORF	Europe DENV-3 ORF	GZ8H (MW720883.1)	North America DENV-3 ORF	The Oceania DENV-3 ORF	South America DENV-3 ORF
Africa DENV-3 ORF		95	98	98	93	98	97	93	97
Asia DENV-3 ORF			94	95	95	94	95	95	95
D191267				99	92	99	96	93	96
DV3syn WT ORF					93	99	96	93	96
Europe DENV-3 ORF						92	94	98	94
GZ8H (MW720883.1)							96	93	96
North America DENV-3 ORF								94	99
The Oceania DENV-3 ORF									94
South America DENV-3 ORF									

The consensus DENV-3 ORF was deduced using the DENV-3 sequences isolated from the corresponding continent and all were retrieved from GenBank. The GenBank accession numbers were showed in Supplementary Figure 1, where prM-E-NS1 consensus sequences were generated.

Supplementary Table 4. Differences of nucleotide and amino acid sequences of D191267, consensus, and ECP-reduced prM-E-NS1 sequences.

Nucleotide position	Amino acid position	D191267 wild-type, codon (aa)	Consensus made from 180 DENV-3 isolates, codon (aa)	ECP-reduced Sequence, codon (aa)	Modified ECP site
448	118	ACT (Thr)	ACT (Thr)	ACC (Thr)	Yes
496	134	GGA (Gly)	GGA (Gly)	GGG (Gly)	Yes
565	157	GAA (Glu)	GAG (Glu)	GAG (Glu)	No
577	161	GAT (Asp)	GAC (Asp)	GAC (Asp)	No
600	169	CAC (His)	CTC (Leu)	CTC (Leu)	No
652	186	TCA (Ser)	TCG (Ser)	TCG (Ser)	No
745	217	CTG (Leu)	CTA (Leu)	CTA (Leu)	No
826	244	CAT (His)	CAC (His)	CAC (His)	No
874	260	ACC (Thr)	ACT (Thr)	ACT (Thr)	No
878	262	TTG (Leu)	TTG (Leu)	CTG (Leu)	Yes
952	286	GTG (Val)	GTA (Val)	GTA (Val)	No
979	295	CTG (Leu)	CTA (Leu)	CTA (Leu)	No
1009	305	CTA (Leu)	CTC (Leu)	CTC (Leu)	No
1018	308	GGG (Gly)	GGT (Gly)	GGT (Gly)	No
1030	312	ACC (Thr)	ACT (Thr)	ACT (Thr)	No
1054	320	ACA (Thr)	ACG (Thr)	ACG (Thr)	No
1055	321	CTG (Leu)	TTG (Leu)	TTG (Leu)	No
1132	346	ACT (Thr)	ACC (Thr)	ACC (Thr)	No
1153	353	AGA (Arg)	AGG (Arg)	AGG (Arg)	No
1175	361	GTT (Val)	ATT (Ile)	ATT (Ile)	No
1189	365	GAA (Glu)	GAG (Glu)	GAG (Glu)	No
1216	374	CAC (His)	CAT (His)	CAT (His)	No
1225	377	GTA (Val)	GTG (Val)	GTG (Val)	No
1228	378	GAT (Asp)	GAC (Asp)	GAC (Asp)	No
1276	394	GTA (Val)	GTA (Val)	GAG (Val)	Yes
1297	401	TGC (Cys)	TGT (Cys)	TGT (Cys)	No
1321	409	GTA (Val)	GTG (Val)	GTG (Val)	No
1357	421	ATT (Ile)	ATC (Ile)	ATC (Ile)	No
1375	427	GAC (Asp)	GAT (Asp)	GAT (Asp)	No
1406-1408	438	ATT (Ile)	GTC (Val)	GTC (Val)	No
1423	443	ACG (Thr)	ACA (Thr)	ACA (Thr)	No
1438	448	ACT (Thr)	ACC (Thr)	ACC (Thr)	No
1439-1440	449	ACT (Thr)	GTT (Val)	GTT (Val)	No
1453	453	TTG (Leu)	TTG (Leu)	TTA (Leu)	Yes
1504	470	GAC (Asp)	GAT (Asp)	GAT (Asp)	No
1519	475	ATC (Ile)	ATT (Ile)	ATT (Ile)	No
1522	476	TTA (Leu)	TTG (Leu)	TTG (Leu)	No
1523-1525	477	CTA (Leu)	TTG (Leu)	CTA (Leu)	Yes

1555	487	CAC (His)	CAT (His)	CAT (His)	No
1618	508	ACT (Thr)	ACC (Thr)	ACC (Thr)	No
1636	514	CTC (Leu)	CTT (Leu)	CTT (Leu)	No
1654	520	AAT (Asn)	AAC (Asn)	AAC (Asn)	No
1714	540	ACT (Thr)	ACC (Thr)	ACC (Thr)	No
1729	545	GCC (Ala)	GCT (Ala)	GCT (Ala)	No
1735	547	GAA (Glu)	GAG (Glu)	GAG (Glu)	No
1759	555	AGC (Ser)	AGT (Ser)	AGT (Ser)	No
1762	556	ATT (Ile)	ATT (Ile)	ATC (Ile)	Yes
1780	562	AAG (Lys)	AAA (Lys)	AAA (Lys)	No
1798	568	GAT (Asp)	GAC (Asp)	GAC (Asp)	No
1802	570	CTG (Leu)	TTG (Leu)	CTG (Leu)	Yes
1835-1836	581	ACG (Thr)	TTG (Leu)	TTG (Leu)	No
1840	582	AAC (Asn)	AAT (Asn)	AAT (Asn)	No
1900	602	GTC (Val)	GTT (Val)	GTT (Val)	No
2005	637	ACT (Thr)	ACT (Thr)	ACC (Thr)	Yes
2020	642	CCC (Pro)	CCT (Pro)	CCT (Pro)	No
2026	644	AAC (Asn)	AAT (Asn)	AAT (Asn)	No
2056	654	AGT (Ser)	AGT (Ser)	AGC (Ser)	Yes
2059	655	AAC (Asn)	AAT (Asn)	AAT (Asn)	No
2065	657	GTA (Val)	GTA (Val)	GTG (Val)	Yes
2068	658	ATT (Ile)	ATT (Ile)	ATC (Ile)	
2083	663	AAT (Asn)	AAA (Lys)	AAA (Lys)	No
2098	668	AAT (Asn)	AAC (Asn)	AAC (Asn)	No
2158	688	AGA (Arg)	AGG (Arg)	AGG (Arg)	No
2218	708	AAC (Asn)	AAT (Asn)	AAT (Asn)	No
2260	722	TAT (Tyr)	TAC (Tyr)	TAC (Tyr)	No
2272	726	TTC (Phe)	TTT (Phe)	TTT (Phe)	No
2284	730	TCT (Ser)	TCC (Ser)	TCC (Ser)	No
2290	732	GTG (Val)	GTA (Val)	GTA (Val)	No
2299	735	ATT (Ile)	ATT (Ile)	ATC (Ile)	Yes
2317	741	TTA (Leu)	TTG (Leu)	TTG (Leu)	No
2344	750	AAT (Asn)	AAC (Asn)	AAC (Asn)	No
2347	751	ACA (Thr)	ACT (Thr)	ACT (Thr)	No
2370-2371	759	GCA (Ala)	GTG (Val)	GTG (Val)	No
2383	763	ATC (Ile)	ATT (Ile)	ATC (Ile)	Yes
2393	767	TTG (Leu)	CTG (Leu)	CTG (Leu)	No
2401	769	GCT (Ala)	GCC (Ala)	GCC (Ala)	No
2407	771	GTA (Val)	GTG (Val)	GTG (Val)	No
2452	786	CTT (Leu)	CTC (Leu)	CTC (Leu)	No
2458	788	TGC (Cys)	TGT (Cys)	TGT (Cys)	No
2479	795	ACC (Thr)	ACT (Thr)	ACT (Thr)	No
2482	796	AAC (Asn)	AAT (Asn)	AAT (Asn)	No
2506	804	CAG (Gln)	CAA (Gln)	CAA (Gln)	No

2515	807	TTC (Phe)	TTT (Phe)	TTT (Phe)	No
2524	810	GAT (Asp)	GAC (Asp)	GAC (Asp)	No
2537	815	TTG (Leu)	TTG (Leu)	CTG (Leu)	Yes
2548	818	GCT (Ala)	GCC (Ala)	GCC (Ala)	No
2566	824	GAA (Glu)	GAG (Glu)	GAG (Glu)	No
2578	828	TGT (Cys)	TGC (Cys)	TGC (Cys)	No
2612	840	TTG (Leu)	TTG (Leu)	CTG (Leu)	Yes
2662	856	AAC (Asn)	AAT (Asn)	AAT (Asn)	No
2680	862	GTC (Val)	GTT (Val)	GTT (Val)	No
2686	864	GGC (Gly)	GGT (Gly)	GGT (Gly)	No
2689	865	GAC (Asp)	GAT (Asp)	GAT (Asp)	No
2691	866	ACA (Thr)	ATA (Thr)	ATA (Thr)	No
2695	867	ATC (Ile)	ATT (Ile)	ATT (Ile)	No
2701	869	GTT (Val)	GTC (Val)	GTC (Val)	No
2707	871	GAA (Glu)	GAG (Glu)	GAG (Glu)	No
2713	873	GGA (Gly)	GGG (Gly)	GGG (Gly)	No
2723	877	TTA (Leu)	CTA (Leu)	CTA (Leu)	No
2752	886	TAC (Tyr)	TAT (Tyr)	TAT (Tyr)	Yes
2791	899	GCA (Ala)	GCT (Ala)	GCA (Ala)	Yes

Supplementary Table 5. Concentration of compounds used for the first-round treatment of DENV-3 replicon cell line, 3-D9

Compound	Concentration 1 (μM)	Concentration 2 (μM)	Compound	Concentration 1 (μM)	Concentration 2 (μM)
A2-P1	2	0.2	C77-P1	2	0.2
A3-P1	2	0.2	C78-P1	2	0.2
A5-P1	2	0.2	C81-P2	0.25	0.025
A7-P2	2	0.2	C88-P1	2	0.2
A8-P1	2	0.2	C100-P1	1	0.1
C04-P1	1	0.1	C100-P2	1	0.1
C05-P1	1	0.1	C102-P1	1	0.1
C06-P1	0.5	0.05	C103-P1	1	0.1
C06-P2	1	0.1	C103-P2	1	0.1
C06-P3	1	0.1	C104-P1	1	0.1
C06-P4	1	0.1	C105-P1	1	0.1
C06-P6	1.5	0.15	C139-P1	2	0.2
C22-P1	0.3	0.03	C140-P1	0.5	0.05
C23-P1	0.25	0.025	C142-P1	2	0.2
C25-P1	0.25	0.025	C143-P2	0.5	0.05
C27-P1	0.25	0.025	C144-P1	5	0.5
C27-P2	0.25	0.025	C146-P1	1	0.1
C27-P3	0.25	0.025	C147-P1	5	0.5
C37-P1	1	0.1	C152-P2	0.75	0.075
C38-P1	2	0.2	C153-P2	2	0.2
C39-P1	2	0.2	C154-P2	2	0.2
C40-P1	0.75	0.075	C156-P1	2	0.2
C40-P2	1	0.1	C158-P1	2	0.2
C40-P3	1	0.1	C169-P1	0.75	0.075
C40-P4	1	0.1	C176-P1	0.45	0.045
			C180A-P1	0.3	0.03
C40-P5	1	0.1	C187-P1	1	0.1
C40-P6	1	0.1	C190-P1	2	0.2
C42-P2	0.25	0.025	C191-P1	1	0.1
C43-P1	0.25	0.025	C192-P1	5	0.5
C45-P1	0.25	0.025	C193-P1	0.75	0.075
C52-P1	0.5	0.05	C194N-P1	2	0.2
C53-P1	0.25	0.025	C195-P1	1	0.1
C54-P1	1	0.1	C197-P1	1.5	0.15
C58-P1	1	0.1	C200-P1	5	0.5
C59-P1	1	0.1	C201-P1	1.5	0.15
C62-P1	0.25	0.025	C204-P1	2	0.2
C62-P2	0.25	0.025	D6-P1	0.2	0.02
C63-P1	0.25	0.025			

C67-P2	1	0.1	2.15	2	0.2
C67-P3	0.25	0.025	2.14-Me	2	0.2
C70-P1	0.25	0.025	2.14-Et	2	0.2
C71-P1	0.25	0.025			
